# Serum APOC1 levels are decreased in young autoantibody positive children who rapidly progress to type 1 diabetes

**DOI:** 10.1038/s41598-023-43039-4

**Published:** 2023-09-24

**Authors:** M. Karoliina Hirvonen, Niina Lietzén, Robert Moulder, Santosh D. Bhosale, Jaakko Koskenniemi, Mari Vähä-Mäkilä, Mirja Nurmio, Matej Orešič, Jorma Ilonen, Jorma Toppari, Riitta Veijola, Heikki Hyöty, Harri Lähdesmäki, Mikael Knip, Lu Cheng, Riitta Lahesmaa

**Affiliations:** 1 Turku Bioscience Centre, University of Turku and Åbo Akademi University, Turku, Finland; 2 InFLAMES Research Flagship Center, University of Turku, Turku, Finland; 3 Department of Biochemistry and Molecular Biology, University of Southern Denmark, Odense, Denmark; 4 Department of Pediatrics, University of Turku and Turku University Hospital, Turku, Finland; 5 Institute of Biomedicine, Research Centre for Integrative Physiology and Pharmacology, and Centre for Population Health Research, University of Turku, Turku, Finland; 6 School of Medical Sciences, Örebro University, Örebro, Sweden; 7 Immunogenetics Laboratory, University of Turku, Turku, Finland; 8 Department of Pediatrics, Research Unit of Clinical Medicine, Medical Research Center, University of Oulu, Oulu, Finland; 9 Department for Children and Adolescents, Medical Research Center, Oulu University Hospital, Oulu, Finland; 10 Faculty of Medicine and Health Technology, Tampere University, Tampere, Finland; 11 Fimlab Laboratories, Tampere, Finland; 12 Department of Computer Science, Aalto University School of Science, Aalto, Finland; 13 Pediatric Research Center, New Children’s Hospital, Helsinki University Hospital, Helsinki, Finland; 14 Research Program for Clinical and Molecular Metabolism, Faculty of Medicine, University of Helsinki, Helsinki, Finland; 15 Department of Pediatrics, Tampere University Hospital, Tampere, Finland; 16 Institute of Biomedicine, University of Turku, Turku, Finland

**Keywords:** Predictive markers, Autoimmunity

## Abstract

Better understanding of the early events in the development of type 1 diabetes is needed to improve prediction and monitoring of the disease progression during the substantially heterogeneous presymptomatic period of the beta cell damaging process. To address this concern, we used mass spectrometry-based proteomics to analyse longitudinal pre-onset plasma sample series from children positive for multiple islet autoantibodies who had rapidly progressed to type 1 diabetes before 4 years of age (n = 10) and compared these with similar measurements from matched children who were either positive for a single autoantibody (n = 10) or autoantibody negative (n = 10). Following statistical analysis of the longitudinal data, targeted serum proteomics was used to verify 11 proteins putatively associated with the disease development in a similar yet independent and larger cohort of children who progressed to the disease within 5 years of age (n = 31) and matched autoantibody negative children (n = 31). These data reiterated extensive age-related trends for protein levels in young children. Further*,* these analyses demonstrated that the serum levels of two peptides unique for apolipoprotein C1 (APOC1) were decreased after the appearance of the first islet autoantibody and remained relatively less abundant in children who progressed to type 1 diabetes, in comparison to autoantibody negative children.

## Introduction

Type 1 diabetes is an immune-mediated disease that results from destruction of the insulin-secreting beta cells in the pancreas, necessitating lifelong treatment with exogenous insulin. The disease is characterized by a presymptomatic period of variable length that precedes the clinical manifestation of the disease at the late stage of beta cell destruction^1^. A better understanding of the events that are associated with the development of type 1 diabetes is needed for the improvement of early prediction and monitoring of the disease and recognition of at risk individuals for clinical interventions and therapy. Currently, the presence of circulating type 1 diabetes-associated autoantibodies is the primary sign of the ongoing disease process and an increased risk for type 1 diabetes^2^. In particular, subjects who develop two or more autoantibodies carry a high probability of developing the disease, whereas those who remain positive for a single autoantibody have a considerably lower risk^3,4^. However, even for multiple autoantibody positive subjects, the interval between the first autoantibody detection that is confirmed (i.e. seroconversion) and the clinical manifestation of the disease is unpredictable, varying from months to decades^5,6^, and the time to diagnosis can only be predicted by following glucose metabolism that starts to deteriorate 1–2 years before the diagnosis^7,8^.

The discovery of early signs of type 1 diabetes has been challenged by the heterogeneity in the rate of disease development. As a source for such signals serum/plasma has been of particular interest because blood is easily accessible and changes in its composition can be indicative of the disease. Accordingly, mass spectrometry (MS)-based proteomics has been used for the detection of circulating type 1 diabetes-associated proteins^9–14^. Recently, technological improvements in throughput, data acquisition and speed, together with the possibility to analyse serum proteins in an unbiased and hypothesis-free manner, have added to the utility of proteomics for the discovery of disease related patterns^15^. Previous type 1 diabetes plasma and serum proteomics analyses have ranged from post-diagnosis cross-sectional comparisons^9–11^ to pre-onset sample series obtained from prospective sample collections^12–14^. Studies of diagnosed patients have used pooled samples to determine type 1 diabetes-associated proteins in newly diagnosed patients^9,11^ and in patients with longer duration of the disease^10^. To establish changes occurring prior to diagnosis, temporal studies of both heterogeneous^12,14^ and more homogeneous populations, although with very few samples per individual^13^, have been reported. Taking into consideration the disease heterogeneity together with the effects of age in children^16–19^ and large inter-individual variability in serum/plasma protein levels^18,20^, these earlier type 1 diabetes serum/plasma proteomics studies have been limited by the number of sampling points or lack of homogeneity in the selection of participants.

In the current study, MS-based proteomics was used in the search of early signs of type 1 diabetes. Focusing on young children who progressed rapidly to type 1 diabetes within months from seroconversion, longitudinal plasma and serum samples collected in the Finnish Type 1 Diabetes Prediction and Prevention (DIPP) study were analysed. Using longitudinal data modelling, plasma protein profiles of multiple autoantibody positive children who developed type 1 diabetes before 4 years of age (progressors) were compared to similar measurements from matched single autoantibody positive (1AAb+) and autoantibody negative (AAb−) children. Statistically significant differences (defined in LonGP analysis in “Methods”) in the plasma protein profiles were observed for several proteins. Out of these, 11 candidates were selected for verification measurements using a targeted proteomics approach and a larger independent yet similar cohort of DIPP children with more frequent sampling points.

## Results

### Detection of type 1 diabetes-associated proteins by discovery proteomics

**Figure 1:**
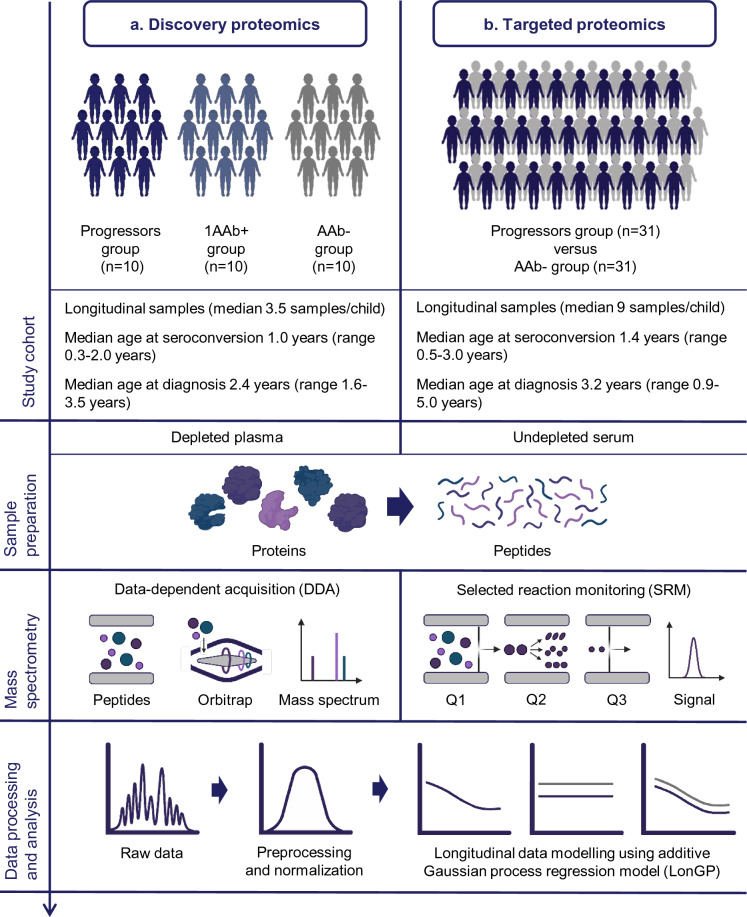
Schematic presentation of the study design. (**a**) First, discovery proteomics was used for the identification of disease-associated proteins after which (**b**) the selected candidates were verified using targeted proteomics. Progressors group = children who progressed to type 1 diabetes. In the discovery proteomics study, all progressors were also positive for ≥ 2 autoantibodies. 1AAb+ group = children positive for a single autoantibody without progression to type 1 diabetes. AAb− group = autoantibody negative children.

To investigate early signs of type 1 diabetes in young children who rapidly progress to the disease after seroconversion, longitudinal sample series were analysed from 10 multiple autoantibody positive DIPP children who were diagnosed with type 1 diabetes under 4 years of age and their matched 1AAb + (n = 10) and AAb- children (n = 10) (Fig. 1a, Supplementary Table S1, Supplementary Fig. S1). The children were matched on the basis of human leukocyte antigen (HLA) risk group, sex, region and date of birth. MS-analysis of these depleted plasma samples resulted in the identification and quantification of 269 protein groups (hereafter referred as proteins), including proteins identified with uniquely associated peptides and proteins that could not be distinguished by the identified peptides and were therefore collapsed to protein groups (Supplementary Table S2), with an average of 254 (± 17) proteins per sample. An additive Gaussian process regression model (LonGP) was used to deconvolute the longitudinal protein patterns into various effects associated with seroconversion, the onset of type 1 diabetes, study groups, age and sex. These effects could separate the study groups, as well as depict the normal variation in plasma proteomes including the effects of age and sex.

### Age is one of the strongest factors affecting plasma proteome in early childhood

**Figure 2:**
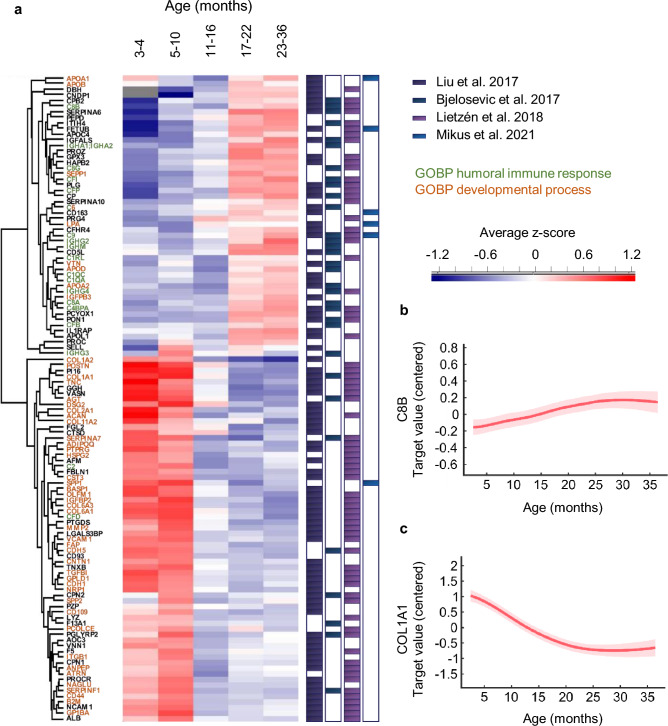
Proteins with age-associated changes in the discovery proteomics cohort in children up to 36 months of age. (**a**) Hierarchical clustering of the 115 proteins that followed statistically significant age-associated changes in LonGP analysis. The proteins clustered into two distinct groups with either increasing or decreasing age trends. Child-specific z-scores were calculated for each protein at all time points, each time point was then assigned to a specific age group and the z-scores were averaged across all children within the age group. Proteins reported to have age-associated changes in previous pediatric publications are indicated in the separate color columns (regardless of the reported trend). The proteins color coded with green and orange indicate the most significantly enriched Gene Ontology classifications with increasing and decreasing age trends, respectively. Examples of the proteins with (**b**) increasing and (**c**) decreasing age trends are included.

In these measurements, 115 out of the total 269 quantified proteins showed statistically significant age-associated trends (Fig. 2a, Supplementary Table S2), the majority of which were concordant with previously published proteomics studies on paediatric serum/plasma samples^16–19^ (Fig. 2a, Supplementary Table S3). The age-associated protein patterns clustered into two distinct groups with either increasing or decreasing age trend (Fig. 2a–c). Sex-associated differences were observed for three immunoglobulins (Supplementary Table S2), which remained lower in boys in the first 18 months of life. Overall, a wide degree of variability in the plasma proteomes was observed both at the protein level and in longitudinal trends.

### Type 1 diabetes-associated changes in protein levels observed in the discovery data

With the alignment of the longitudinal protein profiles of the progressors, 1AAb+ and AAb− children on the basis of seroconversion dates and age at sampling, 14 proteins with seroconversion-associated changes were subsequently identified by LonGP analysis (Table 1, Supplementary Table S2). The largest changes were observed in prolow-density lipoprotein receptor-related protein 1 (LRP1), immunoglobulin J chain (IGJ) and apolipoprotein A4 (APOA4). Among the seroconversion-associated proteins, apolipoprotein(a) (LPA) and insulin-like growth factor binding protein 2 (IGFBP2) also showed age-associated changes.

**Table 1:** Type 1 diabetes-associated protein level changes in the discovery cohort using LonGP. LonGP terms refers to changes associated to the time of seroconversion (“sero”), changes associated to the time of type 1 diabetes diagnosis (“t1d”) or protein level differences observed between the three study groups (“group” and “group × age”). To consider the effect as statistically significant, the explained variation of the effect was required to be > 1%. Selected candidates for the SRM analysis are indicated in the last column. ^a^CD5L was selected for SRM analysis on the basis of its known association with IGHM and the similarity between the individual specific protein profiles of CD5L and IGHM.

Protein ID	Gene name	Protein name	Explained variation of "age" (%)	Disease-associated effect (LonGP term)	Explained variation of the disease-associated effect (%)	Selected for SRM analysis
Q07954	LRP1	Prolow-density lipoprotein receptor-related protein 1	–	"Sero"	21.4	–
P01591	IGJ	Immunoglobulin J chain	–	"Sero"	16.1	x
P06727	APOA4	Apolipoprotein A-IV	–	"Sero"	15.2	x
P20851	C4BPB	C4b-binding protein beta chain	–	"Sero"	6.0	–
P35443	THBS4	Thrombospondin-4	–	"Sero"	4.9	–
P02774	GC	Vitamin D-binding protein	–	"Sero"	4.4	x
P08519	LPA	Apolipoprotein(a)	8.7	"Sero"	4.3	x
P04075	ALDOA	Fructose-bisphosphate aldolase A	–	"Sero"	3.4	–
P43121	MCAM	Cell surface glycoprotein MUC18	–	"Sero"	2.9	–
P02765	AHSG	Alpha-2-HS-glycoprotein	–	"Sero"	2.3	–
P49747	COMP	Cartilage oligomeric matrix protein	–	"Sero"	2.0	–
P18065	IGFBP2	Insulin-like growth factor-binding protein 2	47.7	"Sero"	1.7	x
P02787	TF	Serotransferrin	–	"Sero"	1.4	x
P00748	F12	Coagulation factor XII	–	"Sero"	1.2	x
P54108	CRISP3	Cysteine-rich secretory protein 3	–	"t1d"	3.9	x
P02654	APOC1	Apolipoprotein C-I	–	"t1d"	2.1	x
P02746	C1QB	Complement C1q subcomponent subunit B	1.0	"t1d"	2.1	–
P01871	IGHM	Immunoglobulin heavy constant mu	24.2	"Group" and "Age × group"	9.8 and 4.2	x
P01023	A2M	Alpha-2-macroglobulin	–	"Group"	7.8	–
O43866	CD5L^a^	CD5 antigen-like	26.5	–	–	x

LonGP analysis identified three proteins that exhibited changes associated with the clinical manifestation of type 1 diabetes (Table 1, Supplementary Table S2). These were revealed through the alignment of the longitudinal protein profiles of the progressors according to the date of diagnosis, and 1AAb+ and AAb− children with the diagnosis age of the matched progressors. Statistically significant differences were observed in the longitudinal profiles of cysteine-rich secretory protein 3 (CRISP3), apolipoprotein C1 (APOC1) and complement C1q subcomponent subunit B (C1QB).

The three study groups were distinguished from each other by protein level differences in immunoglobulin heavy constant mu (IGHM) and alpha-2-macroglobulin (A2M), which were both depletion targets (Table 1, Supplementary Table S2). The lowest IGHM plasma levels were detected in the group of progressors, with higher levels in the 1AAb + group, and the highest levels in the AAb- group. One of the functions of the IGHM pentamer is to act as a carrier for CD5 antigen like (CD5L) in the blood^21^. Individual specific longitudinal protein patterns of CD5L were strikingly similar with the IGHM patterns and therefore CD5L was selected as an additional target for verification, although its expression level differences remained below the significance threshold.

### Targeted proteomics analysis of the selected candidates

Based on the discovery study results, verification measurements were made to further explore the protein levels of 11 candidates (Table 1, Supplementary Fig. S2) in an independent set of longitudinal serum samples from 31 DIPP children who were diagnosed with type 1 diabetes before 5 years of age and matched AAb− children (n = 31) (Fig. 1b, Supplementary Table S4, Supplementary Fig. S3). The selection of the candidates was based on LonGP results, a visual inspection of the data and data completeness. An SRM assay was designed for the analysis of 24 peptides representing the 11 candidates and an internal standard. Analysis of the normalised SRM data was carried out at the peptide level using the LonGP model.

Out of the 11 candidates, the age-associated changes observed for four proteins in the discovery study were verified in the targeted proteomics analysis (Supplementary Table S5, Supplementary Fig. S4). Moreover, the targeted proteomics analysis with a larger cohort of children and more frequent sampling points, revealed age-associated trends for peptides representing APOA4, APOC1, IGJ and serotransferrin (TF), as well as one of the measured peptides representing vitamin D-binding protein (GC) (Supplementary Table S5, Supplementary Fig. S4).

**Figure 3:**
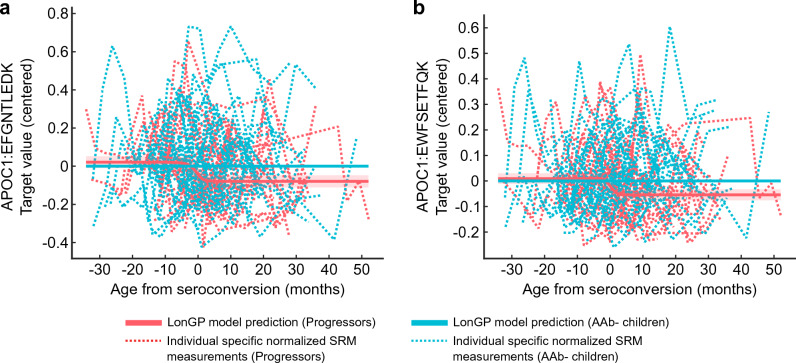
Targeted proteomics verification of APOC1 peptides. LonGP moldels of the APOC1 peptides (**a**) EFGNTLEDK and (**b**) EWFSETFQK showing normalized longitudinal SRM measurements of each individual on the background and on the top the statistically significant seroconversion-associated changes between progressors (red) and AAb- children (blue) (“sero” term in LonGP). The shaded areas are 95% confidence intervals. Progressors = children who progressed to type 1 diabetes. AAb− children = autoantibody negative children.

In terms of verification of the disease-associated changes, the levels of both peptides representing APOC1 were consistently decreased after seroconversion (Fig. 3a,b, Supplementary Table S5).

## Discussion

Better understanding of the early events in the development of type 1 diabetes is needed to help identify increased risk and to monitor the presymptomatic period of the disease that varies conspicuously among individuals. Currently, the detection of autoantibodies provides prognostic markers, which indicate the overall probability of developing the disease. However, heterogeneity in the rate of disease progression has challenged the characterization of the early stages of disease development. To address this challenge and establish protein patterns that would distinguish children who rapidly progress to type 1 diabetes from matched AAb− children, we studied longitudinal plasma and serum proteomes of more homogeneous cohorts of genetically susceptible children, in which the progressors developed the disease before 5 years of age. From these discovery proteomics analysis, 11 proteins showing disease-associated changes were selected for verification using targeted proteomics and a larger independent cohort of children. The measurements revealed how the levels of two peptides representing APOC1 decreased after seroconversion and remained lower in children who developed type 1 diabetes.

In keeping with earlier studies, these data reiterate that age is one of the most dominating factors affecting plasma/serum proteome in early childhood^16–19^. Targeted proteomics analysis of the 11 candidates in a larger cohort with more frequent sampling points, verified age-associated protein level changes for four proteins and identified another five age-associated proteins. Of those five proteins, for APOC1, APOA4 and IGJ, the most prominent effect of age was observed during the first year of life. The identification of these additional trends was likely enabled by the more frequent sampling during the first year of life in the targeted proteomics dataset (up to 4 samples per child in the verification cohort before the age of 13 months, compared with up to 2 samples per child in the discovery cohort). Taken together, longitudinal study designs with frequent sampling points and well-matched controls are key considerations to minimize possible sources of error in serum/plasma discovery proteomics studies in young children. Moreover, quantification differences between plasma and serum have been previously noted, particularly with coagulants and platelet proteins^22^. To avoid such bias in the current study, plasma and serum were not mixed within the study cohorts, and the targets selected for SRM analysis were not among the most significantly altered proteins between plasma and serum^22^.

In the discovery study, lower APOA4 levels were observed after seroconversion to autoantibody positivity both in the progressors and in the 1AAb+ children compared to AAb− children. Similarly, in earlier proteomics studies lower APOA4 levels have been observed in longitudinal serum profiles of multiple autoantibody positive children who later progressed to type 1 diabetes at the median age of 4.1 years^12^, and in seroconverted children with a median age of 3.2 years^13^. In the current verification cohort, however, the disease-associated changes were not observed. Instead, age-associated changes were detected in peptides representing APOA4 (Supplementary Fig. S4). For the discovery measurements, the limited number of samples collected under one year of age, might have caused some bias, as discussed in the previous section. This emphasizes the importance of data completeness in longitudinal studies.

Decreasing levels of IGFBP2 were observed in the discovery cohort in the progressors and in the 1AAb+ children compared to the AAb− children, which is in line with the previous proteomics studies that focused on children during the presymptomatic period of the disease^12,14^. In contrast, higher levels of IGFPB2 were reported in patients with type 1 diabetes, although with longer disease duration^10^. Coxsackievirus B1 (CVB1) infections have been associated with the risk of developing type 1 diabetes-associated autoimmunity and in particular with the appearance of insulin autoantibodies (IAA) as the first autoantibody^23^. In our recent study, persistent CVB1 infection suppressed the secretion of IGFBP2 from human pancreatic ductal-like cell line PANC1^24^. Similarly, IGFBP2 expression was suppressed in a CVB1-infected human lung cancer cell line, and the suppression was reversed after treatment with an antiviral drug^25^. Moreover, the expression of IGFBP2 is the highest in the pancreas and the liver^26^, indicating that the changes in IGFBP2 levels in plasma might reflect changes in its expression in these organs. In the current study, the majority of seroconverted children in the discovery cohort had IAA as the first autoantibody, whereas in the targeted proteomics cohort the proportion was for only one third. Distinct autoantibody profiles have been associated with different disease trajectories^27^, and therefore such differences between the discovery and verification cohorts might influence the ability to verify the disease-associated difference in IGFBP2 levels. Accordingly, it would be important to study IGFBP2 expression specifically in subjects with IAA as their first autoantibody.

Although targeted for immunodepletion in the discovery analyses, plasma IGHM levels separated the three study groups from each other, with levels ranging from the lowest in the group of progressors, intermediate levels in the 1AAb+ group and the highest levels in the AAb− group. However, in the targeted proteomics analysis, the levels of IGHM did not distinguish the progressors from the AAb− children. Nevertheless, reduced total plasma IgM has recently been reported in school aged children with type 1 diabetes^28^. Certain variants of the IGHM gene locus have been associated with an increased risk to type 1 diabetes^29,30^. Interestingly, in a plasma proteomic study of twins, both IGHM and its binding partner CD5L were reported among the top proteins, that were mostly affected by heritability^20^. Further investigation of the risk variants in the IGHM region and their association with serum/plasma IGHM levels remains to be made.

Targeted proteomics analysis of the selected candidates revealed that the levels of two peptides from APOC1 distinguished the children who progressed to type 1 diabetes from AAb− children. A discrepancy was observed between the APOC1 LonGP results in the discovery and the validation dataset, by which the changes were associated with the type 1 diabetes diagnosis and seroconversion, respectively (Supplementary Fig. S2 and Fig. 3a,b). For the discovery dataset, LonGP regression indicated a consecutive peak and trough in APOC1 levels prior to T1D diagnosis (Supplementary Fig. S2), whereas a general decrease in APOC1 after seroconversion was observed in the validation dataset (Fig. 3a,b). In keeping with the coverage and consistency of the validation samples relative to the smaller sample size per individual in the discovery data, we conclude that the data from the progressor group are consistent with a decrease in the APOC1 level after seroconversion. This is described in further detail in the Supplementary Discussion and Supplementary Figs. S5–S7. Closer inspection of data from our earlier longitudinal serum proteomics study of children also demonstrated lower serum APOC1 levels in children who progressed to type 1 diabetes compared to AAb− controls^12^ (see Supplementary Fig. S8). Furthermore, lower plasma and serum abundance of APOC1 was reported in patients with recently diagnosed type 1 diabetes compared to healthy controls although the difference was not statistically significant^9^. In contrast, increased APOC1 levels have been reported in adult patients affected by type 1 diabetes^31^. APOC1 plays important roles in lipid homeostasis, and lipid disorders are commonly diagnosed in patients with type 1 diabetes^32,33^. Several enzymes that participate in lipoprotein metabolism are modulated by APOC1, including cholesterol ester transfer protein (CETP), the activity of which is potentially inhibited by APOC1^31,34^. Increased CETP-activity has been observed in patients with type 1 diabetes and plasma APOC1 concentrations correlated inversely with CETP-activity in normoglycemic-normolipidemic controls but not in individuals with type 1 diabetes, indicating that APOC1 activity could be compromised in this population^31^. The latter study also suggests that the glycation of APOC1 as a result of hyperglycemia in diabetes may impair its inhibitory function. Differences in plasma APOC1 levels have also been previously reported in the context of type 2 diabetes and in relation to nutrition^35,36^. Recently, lower plasma APOC1 levels were reported in adults after caloric restriction and increasing levels in response to glucose consumption^36^. Interestingly, in the latter study, decreased plasma APOC1 was also observed to be associated with undiagnosed type 2 diabetes and prediabetes^36^. To conclude, lower APOC1 levels are detected during the presymptomatic period of type 1 diabetes and in recently diagnosed patients whereas higher APOC1 levels are observed in adult patients. The reasons behind these contrary observations, as well as the possible role of structurally modified or otherwise dysfunctional APOC1, remain to be addressed.

In summary, our data further explored the strength of moderately abundant serum proteins for the characterization of the emerging risk of type diabetes development among HLA-conferred and autoantibody positive children. These analyses reiterated that age is among the most dominating factors affecting plasma/serum proteome in early childhood and should therefore be carefully considered in study designs. Using LonGP modelling, we were able to take into account the nonlinear effect of age while extracting disease-associated changes from the data. From the initial discovery proteomics measurements, several proteins with prior literature associations were noted. The magnitude of changes of the discussed proteins (APOA4, IGFBP2, IGHM, and APOC1) were at a similar level in the discovery data. However, despite replicating the age-associated changes in these well-matched cohorts, the disease-associated signals were challenging to reproduce. This could be partly explained by the subtle levels of disease-associated changes, the relatively low number of participants, dissimilarities in their sample series and autoantibody profiles, in addition to differences in sample storage, preparation and analysis. Nevertheless, we were able to verify that decreased serum levels of two peptides representing APOC1, were associated with rapid progression to type 1 diabetes. In future follow-up studies, APOC1 measurements could be combined with other relevant targets, which might assist with the monitoring of disease progression and stratification for early interventions, especially in young at risk individuals. The causal connection between low APOC1 levels and type 1 diabetes, and the mechanisms involved, remain to be explored.

## Methods

### Study participants

Blood plasma or serum samples, collected in the DIPP study from children (n = 92) carrying HLA-conferred genetic risk for type 1 diabetes^37^, were used for these analyses. The selected participants regularly attended their DIPP clinical study centre (1996–2015), in the cities of Tampere or Turku. At each visit a non-fasting plasma or serum sample was collected. The samples were stored at − 80 °C until analysis. The parents gave their written informed consent for participation. The study followed the guidelines of the Declaration of Helsinki and the Regional Ethics Committee of the Joint Municipal Authority of Northern Ostrobothnia Hospital District, Oulu, Finland, approved the study protocol.

### Discovery proteomics

A subset of ten children who had developed multiple (≥ 2) autoantibodies and who all had later developed type 1 diabetes before 4 years of age were defined as progressors. Each progressor was matched, on the basis of HLA risk group, sex, region and date of birth (± 1.4 years), with one child testing positive for a single autoantibody (1AAb+) and another autoantibody negative child (AAb−). For each child, a longitudinal series, with up to four follow-up plasma samples was analysed (Supplementary Table S1, Supplementary Fig. S1). The samples were collected between 3 and 36 months of age, 101 samples in total. Neither the 1AAb+ nor AAb− children developed diabetes, and the AAb- remained autoantibody negative during the follow-up period.

The sample series from each matched triplet (formed by progressor, 1AAb+ and AAb− child) were prepared and analysed batch-wise in a blinded fashion. The sample preparation, including immunodepletion of the 12 most abundant plasma proteins (Pierce Top 12 Abundant Protein Depletion Spin Columns, Thermo Scientific, USA), was carried out as described in Lietzén et al.^18^ with modified double desalting. Retention time standard peptides (iRT, Biognosys, Switzerland) were spiked into each sample for quality control of the liquid chromatography tandem mass spectrometry (LC–MS/MS) analyses.

Each sample was analysed in triplicate using Q Exactive™ Hybrid Quadrupole-Orbitrap™ mass spectrometer (Thermo Fisher Scientific, Bremen, Germany) that was interfaced with an EASY-nLC 1000 liquid chromatograph (Thermo Fisher Scientific) with a nano-electrospray ion source (Thermo Fisher Scientific). See Supplementary Methods online for further details of the LC–MS/MS method.

### Discovery proteomics data processing

The LC–MS/MS data was processed using MaxQuant software version 1.5.5.1^38^ with the built-in Andromeda search engine^39^. A combined SwissProt human and TrEMBL enterovirus protein sequence database (April 2014, 63,470 entries) with added iRT peptide sequences and common contaminants was used. Label-free quantification (LFQ) was selected with trypsin digestion (maximum two missed cleavages), methionine oxidation as a variable modification and cysteine carbamidomethylation as a fixed modification. A false discovery rate of 1% for protein and peptide levels was applied, determined by searching the reversed database. Otherwise, MaxQuant default settings were used with selection of “match between runs”.

The MaxQuant output file was pre-processed with Perseus software^40^ using normalised LFQ intensities. Contaminants and proteins detected with less than two unique peptides, or present in less than 50% of the samples were filtered out. The median intensities were calculated from the technical replicates and log2 transformed for statistical analyses. See Supplementary Methods online for further details.

### Targeted proteomics verification

For the verification of observations from the discovery analyses, a separate series of 524 serum samples from 62 DIPP children were analysed. These were collected between 2 and 60 months of age from children diagnosed with type 1 diabetes before 5 years of age (n = 31) and their matched AAb− children (n = 31) (difference in dates of birth of the matched pairs ± 3.4 months), with a median of nine samples per child (Supplementary Table S4, Supplementary Fig. S3). The AAb− children remained autoantibody negative and did not develop diabetes during the follow-up period.

The verification samples were prepared without immunodepletion of the abundant proteins, as described elsewhere^41^. Heavy isotope-labeled synthetic peptide analogues (PEPotec, Thermo Fisher Scientific, USA) for the protein targets were spiked (~ 10 fmol/µl) into the digests together with retention time standards (MSRT1, Sigma-Aldrich, USA). The targets and additional details are provided in Supplementary Table S6 and Supplementary Methods online.

A TSQ Vantage Triple Quadrupole Mass Spectrometer (Thermo Scientific, Bremen, Germany) coupled with an Easy-nLC 1000 liquid chromatograph (Thermo Scientific) was used to perform the selected reaction monitoring (SRM). The analyses were conducted in three batches, using the same column configuration as described for the discovery measurements and slightly varying chromatographic conditions. Aliquots of a reference sample were periodically analysed to monitor the performance and reproducibility of the assay (see Supplementary Table S7). Skyline software^42^ was used to develop the data acquisition method and inspect the data. The peptide peak areas were normalised by dividing the total peak area of each peptide with the sum of the peak areas of endogenous alpha-1B-glycoprotein (A1BG) peptides (SGLSTGWTQLSK and ATWSGAVLAGR) in the same sample. A1BG was selected as a global standard based on our previous studies, in which it was among the most stable proteins in serum and plasma in children^12,18^. The exported peptide level data was then analysed with LonGP.

### LonGP analysis

LonGP was used to model the longitudinal changes in both datasets^43^. Selected continuous covariates included age (days from birth to sampling date), sero (days from seroconversion date to sampling date) and t1d (days from type 1 diabetes diagnosis date to sampling date), which were converted into the unit of months. Discrete covariates included sex (male = 1, female = 0), group (progressors = 1, 1AAb+ = 2, AAb− = 3), pair (pair id) and id (individual id). Interaction flags for sero and t1d were set to false. Non-stationary kernels were used for “sero” and “t1d”, where we used the kernel parameters (a = 0.5, b = 0, c = 40) such that the main variations occur between − 12 month to + 12 month. For the data from targeted analysis, an additional discrete covariate “batch” was used to represent different MS batches for the same peptide. Default LonGP parameters were used. The preprocessed data files and parameter specification files for both discovery and validation experiments are provided in a supplementary file (see Supplementary Data online)**.** To be considered statistically significant, the effect was required to be included in the final cross-validated model and the explained variation of the effect to be > 1%. Although the variation threshold of 1% corresponds to a small effect size, the size of some effects was notably larger than 1%, and for consistency the explained variation for each protein is reported.

### Supplementary Information

Supplementary Information 1.

Supplementary Information 2.

## Data Availability

The mass spectrometry discovery proteomics data have been deposited to the ProteomeXchange Consortium via the PRIDE^44^ partner repository with the dataset identifier PXD033744. The raw SRM data and Skyline documents are available through Panorama Public^45^ (https://panoramaweb.org/APOC1_rapidT1D.url) with the dataset identifier PXD033946.

## References

[1] Insel RA (2015). Staging presymptomatic type 1 diabetes: A scientific statement of JDRF, the Endocrine Society, and the American Diabetes Association. Diabetes Care.

[2] Knip M (2005). Environmental triggers and determinants of type 1 diabetes. Diabetes.

[3] Ziegler AG (2013). Seroconversion to multiple islet autoantibodies and risk of progression to diabetes in children. JAMA.

[4] Anand V (2021). Islet autoimmunity and HLA markers of presymptomatic and clinical type 1 diabetes: Joint analyses of prospective cohort studies in Finland, Germany, Sweden, and the U.S. Diabetes Care.

[5] Knip M (2002). Natural course of preclinical type 1 diabetes. Horm. Res..

[6] Bauer W (2019). Age at seroconversion, HLA genotype, and specificity of autoantibodies in progression of islet autoimmunity in childhood. J. Clin. Endocrinol. Metab..

[7] Helminen O (2015). OGTT and random plasma glucose in the prediction of type 1 diabetes and time to diagnosis. Diabetologia.

[8] Helminen O (2015). HbA1c predicts time to diagnosis of type 1 diabetes in children at risk. Diabetes.

[9] Metz TO (2008). Application of proteomics in the discovery of candidate protein biomarkers in a diabetes autoantibody standardization program sample subset. J. Proteome Res..

[10] Zhi W (2011). Discovery and validation of serum protein changes in type 1 diabetes patients using high throughput two dimensional liquid chromatography-mass spectrometry and immunoassays. Mol. Cell. Proteomics MCP.

[11] Zhang Q (2013). Serum proteomics reveals systemic dysregulation of innate immunity in type 1 diabetes. J. Exp. Med..

[12] Moulder R (2015). Serum proteomes distinguish children developing type 1 diabetes in a cohort with HLA-conferred susceptibility. Diabetes.

[13] von Toerne C (2017). Peptide serum markers in islet autoantibody-positive children. Diabetologia.

[14] Liu C-W (2018). Temporal expression profiling of plasma proteins reveals oxidative stress in early stages of Type 1 Diabetes progression. J. Proteomics.

[15] Aebersold R, Mann M (2016). Mass-spectrometric exploration of proteome structure and function. Nature.

[16] Liu C-W (2017). Temporal profiles of plasma proteome during childhood development. J. Proteomics.

[17] Bjelosevic S (2017). Quantitative age-specific variability of plasma proteins in healthy neonates, children and adults. Mol. Cell. Proteomics MCP.

[18] Lietzén N (2018). Characterization and non-parametric modeling of the developing serum proteome during infancy and early childhood. Sci. Rep..

[19] Mikus M (2021). Protein profiles in plasma: Development from infancy to 5 years of age. Proteomics. Clin. Appl..

[20] Liu Y (2015). Quantitative variability of 342 plasma proteins in a human twin population. Mol. Syst. Biol..

[21] Hiramoto E (2018). The IgM pentamer is an asymmetric pentagon with an open groove that binds the AIM protein. Sci. Adv..

[22] Geyer PE (2019). Plasma Proteome Profiling to detect and avoid sample-related biases in biomarker studies. EMBO Mol. Med..

[23] Sioofy-Khojine A-B (2018). Coxsackievirus B1 infections are associated with the initiation of insulin-driven autoimmunity that progresses to type 1 diabetes. Diabetologia.

[24] Lietzén N (2019). Coxsackievirus B persistence modifies the proteome and the secretome of pancreatic ductal cells. iScience.

[25] Ianevski A (2020). Identification and tracking of antiviral drug combinations. Viruses.

[26] Uhlén M (2015). Proteomics. Tissue-based map of the human proteome. Science.

[27] Kwon BC (2022). Progression of type 1 diabetes from latency to symptomatic disease is predicted by distinct autoimmune trajectories. Nat. Commun..

[28] Harms RZ (2020). Confirmation and identification of biomarkers implicating environmental triggers in the pathogenesis of type 1 diabetes. Front. Immunol..

[29] Rolim I (2017). Immunoglobulin M gene association with autoantibody reactivity and type 1 diabetes. Immunogenetics.

[30] Ferjeni Z, Raouia F, Abida O, Penha-Gonçalves C, Masmoudi H (2022). Association of IGHM polymorphisms with susceptibility to type 1 diabetes. Immunol. Res..

[31] Bouillet B (2014). Glycation of apolipoprotein C1 impairs its CETP inhibitory property: Pathophysiological relevance in patients with type 1 and type 2 diabetes. Diabetes Care.

[32] Fuior EV, Gafencu AV (2019). Apolipoprotein C1: Its pleiotropic effects in lipid metabolism and beyond. Int. J. Mol. Sci..

[33] Vergès B (2020). Dyslipidemia in type 1 diabetes: AMaskedDanger. Trends Endocrinol. Metab..

[34] Gautier T (2000). Human apolipoprotein C-I accounts for the ability of plasma high density lipoproteins to inhibit the cholesteryl ester transfer protein activity. J. Biol. Chem..

[35] Rouland A (2022). Role of apolipoprotein C1 in lipoprotein metabolism, atherosclerosis and diabetes: A systematic review. Cardiovasc. Diabetol..

[36] Vernardis SI (2023). The impact of acute nutritional interventions on the plasma proteome. J. Clin. Endocrinol. Metab..

[37] Kupila A (2001). Feasibility of genetic and immunological prediction of type I diabetes in a population-based birth cohort. Diabetologia.

[38] Cox J, Mann M (2008). MaxQuant enables high peptide identification rates, individualized p.p.b.-range mass accuracies and proteome-wide protein quantification. Nat. Biotechnol..

[39] Cox J (2011). Andromeda: A peptide search engine integrated into the MaxQuant environment. J. Proteome Res..

[40] Tyanova S (2016). The Perseus computational platform for comprehensive analysis of (prote)omics data. Nat. Methods.

[41] Bhosale SD, Moulder R, Kouvonen P, Lahesmaa R, Goodlett DR (2017). Mass spectrometry-based serum proteomics for biomarker discovery and validation. Methods Mol. Biol..

[42] MacLean B (2010). Skyline: An open source document editor for creating and analyzing targeted proteomics experiments. Bioinformatics.

[43] Cheng L (2019). An additive Gaussian process regression model for interpretable non-parametric analysis of longitudinal data. Nat. Commun..

[44] Perez-Riverol Y (2022). The PRIDE database resources in 2022: A hub for mass spectrometry-based proteomics evidences. Nucleic Acids Res..

[45] Sharma V (2018). Panorama public: A public repository for quantitative data sets processed in skyline. Mol. Cell. Proteomics.

