# Supplementary material for: Serum APOC1 levels are decreased in young autoantibody positive children who rapidly progress to type 1 diabetes
Source: Sci Rep. 2023 Sep 24;13:15941. doi: 10.1038/s41598-023-43039-4 (PMC10518308; doi:10.1038/s41598-023-43039-4)
Supplement: Supplementary file 1 — Supplementary Information 1. [file 41598_2023_43039_MOESM1_ESM.pdf]

SUPPLEMENTARY INFORMATION

SUPPLEMENTARY METHODS

Discovery proteomics LC-MS/MS method

The peptides were first loaded into 0.1 x 20 mm pre-column in buffer A (0.1 % (v/v) formic acid, 2 % acetonitrile), followed by separation in a 75 µm x 150 mm analytical column. Both columns were packed with 5 µm C18 silica particles (Michrom Bioresources, USA). The peptides were separated using a 90 min binary gradient at a flow rate of 300 nl/min, starting with a linear 53-min increase from 2 % to 20 % of buffer B (0.1 % formic acid, 95 % acetonitrile), followed by a 22-min increase to 40 % of B and a 2-min increase to 100 % of B, before 13 min isocratic period with 100 % B. The full-scan resolution was set to 140,000 at m/z 200 and MS1 spectra were collected in the range of 300-2000 m/z. The AGC target value was set to 3 x 10^6 and the maximum injection time was 100 ms. Higher-energy C-trap dissociation was used for fragmentation of the 10 most intense precursor ions with the normalized collision energy of 27 eV and with an isolation window of 2.0 m/z. MS2 scans were collected at a resolution of 17 500 at m/z 200 with an AGC target value of 5 x 10^4 in the range of 200-2000 m/z and with a maximum fill time of 250 ms. To avoid repeated selection of identical peptide ions, the dynamic exclusion window was set to 20 s.

Discovery proteomics data processing

The MaxQuant output file was pre-processed with Perseus software using normalised LFQ intensities. Proteins that were only identified on the basis of one variable modification site and reverse hits were first removed. Subsequently, proteins marked as contaminants and not present in the intended protein sequence databases were filtered out manually, and only proteins that had two or more unique peptides were kept. The median LFQ value for each sample was calculated from the three technical replicates with the requirement of minimum two valid values per sample. Lastly, proteins that were present in at least 50 % of the samples were kept in the final data matrix. All the statistical analyses were made using log_2 intensity values.

Targeted proteomics verification

With the batch-wise analysis of the samples, aliquots of a pooled serum sample, spiked with the synthetic heavy-peptide mixture, were periodically measured to check signal intensity, retention time alignment and provide an indication of the reproducibility of the method. The %RSD of the raw peak intensities are summarized in Supplementary Table S7. This data is combined from Batch 1, Batch 2 and Batch 3 and displayed in the Panorama Skyline files. The data is shown in for the Light isotope (endogenous), Heavy isotope (synthetic) peptides and for the ratio of the areas of light to heavy.

SUPPLEMENTARY DISCUSSION

APOC1 verification results

A discrepancy was noted between the APOC1 LonGP results in the discovery and validation datasets. This can be seen in the representations shown in Supplementary Fig. S2b and Fig. 3a-b. These indicate a "t1d" associated (Supplementary Fig. S2b) and a "sero" associated effect, (Fig. 3a-b), and reflect differences in the sampling frequency and different aspects of the data. Further analysis and evaluation of this is presented as follows.

For each individual in the discovery and validation datasets, the protein/peptide baseline change after seroconversion was calculated. In the LonGP regression of the discovery dataset, a peak [-7, -5] and trough region [-4, -2], (i.e. 5-7 or 2-4 months prior to diagnosis, Supplementary Fig. S2b) was seen in the progressors group. From the LonGP regression of the validation dataset (Fig. 3a-b), a general baseline decrease after seroconversion was seen in the progressors group. As further illustrated in Supplementary Fig. S5, the baseline levels of APOC1 decreased more in the progressors group than those of the AAb- group. This change was similarly observed in the discovery dataset (Supplementary Fig. S6), although was not significant (t-test, p=0.87). Supplementary Fig. S7 further illustrates the baseline difference between the peak and trough region in the validation dataset, which is consistent with a greater difference between the peak and trough regions in the progressor group (t-test, p=0.58 and 0.63).

Due to the superior temporal coverage provided by the validation data, we concluded that the validation data verify that the APOC1 levels in progressors group decrease after seroconversion.

SUPPLEMENTARY TABLES

Supplementary Table S1. Summary and plasma sample collection of children selected for the discovery proteomics analysis.

Matched triplet	Status	Sex	HLA risk group	HLA haplotype 1	HLA haplotype 2	First autoantibody	Overall autoantibodies detected	Age at sample collection (days)	Seroconversion age (days)	Diagnosis age (days)	Discovery analysis code
1	Progressor	Female	Moderately increased risk	DRB1*0401 - DQA1*03 - DQB1*0302	(DR1/10) - DQB1*0501	GADA	GADA, ICA,IAA, IA2A	369	733	1291	15B
1	Progressor	Female	Moderately increased risk	DRB1*0401 - DQA1*03 - DQB1*0302	(DR1/10) - DQB1*0501	GADA	GADA, ICA,IAA, IA2A	733	733	1291	15I
1	1AAb+	Female	Moderately increased risk	DRB1*0401 - DQA1*03 - DQB1*0302	(DR9) - DQA1*03 - DQB1*0303	ICA	ICA, IAA	372	552	NA	15C
1	1AAb+	Female	Moderately increased risk	DRB1*0401 - DQA1*03 - DQB1*0302	(DR9) - DQA1*03 - DQB1*0303	ICA	ICA, IAA	742	552	NA	15F
1	1AAb+	Female	Moderately increased risk	DRB1*0401 - DQA1*03 - DQB1*0302	(DR9) - DQA1*03 - DQB1*0303	ICA	ICA, IAA	1108	552	NA	15H
1	Aab-	Female	Moderately increased risk	DRB1*0401 - DQA1*03 - DQB1*0302	(DR1/10) - DQB1*0501	NA	NA	185	NA	NA	15E
1	Aab-	Female	Moderately increased risk	DRB1*0401 - DQA1*03 - DQB1*0302	(DR1/10) - DQB1*0501	NA	NA	367	NA	NA	15G
1	Aab-	Female	Moderately increased risk	DRB1*0401 - DQA1*03 - DQB1*0302	(DR1/10) - DQB1*0501	NA	NA	734	NA	NA	15L
1	Aab-	Female	Moderately increased risk	DRB1*0401 - DQA1*03 - DQB1*0302	(DR1/10) - DQB1*0501	NA	NA	1101	NA	NA	15A
2	Progressor	Male	Slightly increased risk	DRB1*0404 - DQA1*03 - DQB1*0302	(DR1/10) - DQB1*0501	GADA, ICA,IAA, IA2A	GADA, ICA,IAA, IA2A	364	552	897	20J
2	Progressor	Male	Slightly increased risk	DRB1*0404 - DQA1*03 - DQB1*0302	(DR1/10) - DQB1*0501	GADA, ICA,IAA, IA2A	GADA, ICA,IAA, IA2A	643	552	897	20A
2	Progressor	Male	Slightly increased risk	DRB1*0404 - DQA1*03 - DQB1*0302	(DR1/10) - DQB1*0501	GADA, ICA,IAA, IA2A	GADA, ICA,IAA, IA2A	782	552	897	20B

2	1AAb+	Male	Slightly increased risk	DRB1*0401 - DQA1*03 - DQB1*0302	(DR13) - DQB1*0603	IAA	IAA	375	185	NA	20D
2	1AAb+	Male	Slightly increased risk	DRB1*0401 - DQA1*03 - DQB1*0302	(DR13) - DQB1*0603	IAA	IAA	659	185	NA	20G
2	1AAb+	Male	Slightly increased risk	DRB1*0401 - DQA1*03 - DQB1*0302	(DR13) - DQB1*0603	IAA	IAA	744	185	NA	20I
2	AAb-	Male	Moderately increased risk	DRB1*0401 - DQA1*03 - DQB1*0302	(DR13) - DQB1*0604	NA	NA	188	NA	NA	20E
2	AAb-	Male	Moderately increased risk	DRB1*0401 - DQA1*03 - DQB1*0302	(DR13) - DQB1*0604	NA	NA	395	NA	NA	20L
2	AAb-	Male	Moderately increased risk	DRB1*0401 - DQA1*03 - DQB1*0302	(DR13) - DQB1*0604	NA	NA	546	NA	NA	20K
2	AAb-	Male	Moderately increased risk	DRB1*0401 - DQA1*03 - DQB1*0302	(DR13) - DQB1*0604	NA	NA	733	NA	NA	20H
3	Progressor	Male	Neutral	DRB1*0401 - DQA1*03 - DQB1*0302	(DR7) - DQA1*0201 - DQB1*0303	ICA, IAA	ICA, IAA, GADA, IA2A	372	562	783	21A
3	Progressor	Male	Neutral	DRB1*0401 - DQA1*03 - DQB1*0302	(DR7) - DQA1*0201 - DQB1*0303	ICA, IAA	ICA, IAA, GADA, IA2A	652	562	783	21C
3	Progressor	Male	Neutral	DRB1*0401 - DQA1*03 - DQB1*0302	(DR7) - DQA1*0201 - DQB1*0303	ICA, IAA	ICA, IAA, GADA, IA2A	704	562	783	21G
3	1AAb+	Male				IAA	IAA	185	546	NA	21H
3	1AAb+	Male				IAA	IAA	375	546	NA	21D
3	1AAb+	Male				IAA	IAA	661	546	NA	21F
3	1AAb+	Male				IAA	IAA	754	546	NA	21K
3	AAb-	Male	Moderately increased risk	DRB1*0401 - DQA1*03 - DQB1*0302	(DR13) - DQB1*0604	NA	NA	164	NA	NA	21L
3	AAb-	Male	Moderately increased risk	DRB1*0401 - DQA1*03 - DQB1*0302	(DR13) - DQB1*0604	NA	NA	380	NA	NA	21B
3	AAb-	Male	Moderately increased risk	DRB1*0401 - DQA1*03 - DQB1*0302	(DR13) - DQB1*0604	NA	NA	553	NA	NA	21I

3	AAb-	Male	Moderately increased risk	DRB1*0401 - DQA1*03 - DQB1*0302	(DR13) - DQB1*0604	NA	NA	735	NA	NA	21J
4	Progressor	Female	Neutral	(DR3) - DQA1*05 - DQB1*02	DRB1*0403 - DQA1*03 - DQB1*0302	IAA	IAA, ICA, IA2A, GADA	178	373	893	22H
4	Progressor	Female	Neutral	(DR3) - DQA1*05 - DQB1*02	DRB1*0403 - DQA1*03 - DQB1*0302	IAA	IAA, ICA, IA2A, GADA	554	373	893	22D
4	Progressor	Female	Neutral	(DR3) - DQA1*05 - DQB1*02	DRB1*0403 - DQA1*03 - DQB1*0302	IAA	IAA, ICA, IA2A, GADA	736	373	893	22E
4	1AAb+	Female	Moderately increased risk	DRB1*0401 - DQA1*03 - DQB1*0302	(DR9) - DQA1*03 - DQB1*0303	IAA	IAA	184	745	NA	22B
4	1AAb+	Female	Moderately increased risk	DRB1*0401 - DQA1*03 - DQB1*0302	(DR9) - DQA1*03 - DQB1*0303	IAA	IAA	557	745	NA	22C
4	1AAb+	Female	Moderately increased risk	DRB1*0401 - DQA1*03 - DQB1*0302	(DR9) - DQA1*03 - DQB1*0303	IAA	IAA	836	745	NA	22K
4	AAb-	Female	Neutral	(DR9) - DQA1*03 - DQB1*0303	(DR1/10) - DQB1*0501	NA	NA	167	NA	NA	22F
4	AAb-	Female	Neutral	(DR9) - DQA1*03 - DQB1*0303	(DR1/10) - DQB1*0501	NA	NA	370	NA	NA	22J
4	AAb-	Female	Neutral	(DR9) - DQA1*03 - DQB1*0303	(DR1/10) - DQB1*0501	NA	NA	728	NA	NA	22A
5	Progressor	Female	Moderately increased risk	DRB1*0401 - DQA1*03 - DQB1*0302	(DR1/10) - DQB1*0501	IAA	IAA, ICA, GADA	98	370	884	24G
5	Progressor	Female	Moderately increased risk	DRB1*0401 - DQA1*03 - DQB1*0302	(DR1/10) - DQB1*0501	IAA	IAA, ICA, GADA	189	370	884	24A
5	Progressor	Female	Moderately increased risk	DRB1*0401 - DQA1*03 - DQB1*0302	(DR1/10) - DQB1*0501	IAA	IAA, ICA, GADA	462	370	884	24K
5	Progressor	Female	Moderately increased risk	DRB1*0401 - DQA1*03 - DQB1*0302	(DR1/10) - DQB1*0501	IAA	IAA, ICA, GADA	729	370	884	24C
5	1AAb+	Female	Slightly increased risk	DRB1*0401 - DQA1*03 - DQB1*0302	(DR13) - DQB1*0603	GADA	GADA	107	186	NA	24H

5	1AAb+	Female	Slightly increased risk	DRB1*0401 - DQA1*03 - DQB1*0302	(DR13) - DQB1*0603	GADA	GADA	186	186	NA	24E
5	1AAb+	Female	Slightly increased risk	DRB1*0401 - DQA1*03 - DQB1*0302	(DR13) - DQB1*0603	GADA	GADA	423	186	NA	24O
5	1AAb+	Female	Slightly increased risk	DRB1*0401 - DQA1*03 - DQB1*0302	(DR13) - DQB1*0603	GADA	GADA	710	186	NA	24N
5	AAb-	Female	Moderately increased risk	DRB1*0401 - DQA1*03 - DQB1*0302	(DR1/10) - DQB1*0501	NA	NA	88	NA	NA	24I
5	AAb-	Female	Moderately increased risk	DRB1*0401 - DQA1*03 - DQB1*0302	(DR1/10) - DQB1*0501	NA	NA	183	NA	NA	24L
5	AAb-	Female	Moderately increased risk	DRB1*0401 - DQA1*03 - DQB1*0302	(DR1/10) - DQB1*0501	NA	NA	550	NA	NA	24M
5	AAb-	Female	Moderately increased risk	DRB1*0401 - DQA1*03 - DQB1*0302	(DR1/10) - DQB1*0501	NA	NA	759	NA	NA	24B
6	Progressor	Female	Slightly increased risk	(DR3) - DQA1*05 - DQB1*02	(DR9) - DQA1*03 - DQB1*0303	ICA, IAA, GADA	ICA, IAA, GADA, IA2A	258	552	1094	26J
6	Progressor	Female	Slightly increased risk	(DR3) - DQA1*05 - DQB1*02	(DR9) - DQA1*03 - DQB1*0303	ICA, IAA, GADA	ICA, IAA, GADA, IA2A	384	552	1094	26H
6	Progressor	Female	Slightly increased risk	(DR3) - DQA1*05 - DQB1*02	(DR9) - DQA1*03 - DQB1*0303	ICA, IAA, GADA	ICA, IAA, GADA, IA2A	640	552	1094	26K
6	Progressor	Female	Slightly increased risk	(DR3) - DQA1*05 - DQB1*02	(DR9) - DQA1*03 - DQB1*0303	ICA, IAA, GADA	ICA, IAA, GADA, IA2A	927	552	1094	26C
6	1AAb+	Female	Slightly increased risk	(DR3) - DQA1*05 - DQB1*02	(DR9) - DQA1*03 - DQB1*0303	IAA	NA	295	184	NA	26O
6	1AAb+	Female	Slightly increased risk	(DR3) - DQA1*05 - DQB1*02	(DR9) - DQA1*03 - DQB1*0303	IAA	NA	379	184	NA	26B
6	1AAb+	Female	Slightly increased risk	(DR3) - DQA1*05 - DQB1*02	(DR9) - DQA1*03 - DQB1*0303	IAA	NA	750	184	NA	26L
6	1AAb+	Female	Slightly increased risk	(DR3) - DQA1*05 - DQB1*02	(DR9) - DQA1*03 - DQB1*0303	IAA	NA	941	184	NA	26N

6	AAb-	Female	Moderately increased risk	DRB1*0401 - DQA1*03 - DQB1*0302	(DR1/10) - DQB1*0501	NA	NA	184	NA	NA	26M
6	AAb-	Female	Moderately increased risk	DRB1*0401 - DQA1*03 - DQB1*0302	(DR1/10) - DQB1*0501	NA	NA	368	NA	NA	26E
6	AAb-	Female	Moderately increased risk	DRB1*0401 - DQA1*03 - DQB1*0302	(DR1/10) - DQB1*0501	NA	NA	731	NA	NA	26D
6	AAb-	Female	Moderately increased risk	DRB1*0401 - DQA1*03 - DQB1*0302	(DR1/10) - DQB1*0501	NA	NA	1096	NA	NA	26F
7	Progressor	Male	Moderately increased risk	DRB1*0401 - DQA1*03 - DQB1*0302	(DR1/10) - DQB1*0501	IAA	IAA, ICA, GADA,IA2A	76	364	1032	30M
7	Progressor	Male	Moderately increased risk	DRB1*0401 - DQA1*03 - DQB1*0302	(DR1/10) - DQB1*0501	IAA	IAA, ICA, GADA,IA2A	210	364	1032	30B
7	Progressor	Male	Moderately increased risk	DRB1*0401 - DQA1*03 - DQB1*0302	(DR1/10) - DQB1*0501	IAA	IAA, ICA, GADA,IA2A	467	364	1032	30N
7	Progressor	Male	Moderately increased risk	DRB1*0401 - DQA1*03 - DQB1*0302	(DR1/10) - DQB1*0501	IAA	IAA, ICA, GADA,IA2A	824	364	1032	30J
7	1AAb+	Male	Moderately increased risk	(DR7) - DQA1*0201 - DQB1*02	DRB1*0401 - DQA1*03 - DQB1*0302	GADA	NA	93	182	NA	30F
7	1AAb+	Male	Moderately increased risk	(DR7) - DQA1*0201 - DQB1*02	DRB1*0401 - DQA1*03 - DQB1*0302	GADA	NA	182	182	NA	30L
7	1AAb+	Male	Moderately increased risk	(DR7) - DQA1*0201 - DQB1*02	DRB1*0401 - DQA1*03 - DQB1*0302	GADA	NA	475	182	NA	30G
7	1AAb+	Male	Moderately increased risk	(DR7) - DQA1*0201 - DQB1*02	DRB1*0401 - DQA1*03 - DQB1*0302	GADA	NA	800	182	NA	30C
7	AAb-	Male	Moderately increased risk	DRB1*0401 - DQA1*03 - DQB1*0302	(DR9) - DQA1*03 - DQB1*0303	NA	NA	91	NA	NA	30K
7	AAb-	Male	Moderately increased risk	DRB1*0401 - DQA1*03 - DQB1*0302	(DR9) - DQA1*03 - DQB1*0303	NA	NA	190	NA	NA	30A
7	AAb-	Male	Moderately increased risk	DRB1*0401 - DQA1*03 - DQB1*0302	(DR9) - DQA1*03 - DQB1*0303	NA	NA	547	NA	NA	30E

7	AAb-	Male	Moderately increased risk	DRB1*0401 - DQA1*03 - DQB1*0302	(DR9) - DQA1*03 - DQB1*0303	NA	NA	730	NA	NA	30O
8	Progressor	Male	High risk	(DR3) - DQA1*05 - DQB1*02	DRB1*0401 - DQA1*03 - DQB1*0302	ICA, IAA	ICA, IAA, GADA, IA2A	197	371	676	32J
8	Progressor	Male	High risk	(DR3) - DQA1*05 - DQB1*02	DRB1*0401 - DQA1*03 - DQB1*0302	ICA, IAA	ICA, IAA, GADA, IA2A	488	371	676	32F
8	Progressor	Male	High risk	(DR3) - DQA1*05 - DQB1*02	DRB1*0401 - DQA1*03 - DQB1*0302	ICA, IAA	ICA, IAA, GADA, IA2A	553	371	676	32H
8	1AAb+	Male	High risk	(DR3) - DQA1*05 - DQB1*02	DRB1*0401 - DQA1*03 - DQB1*0302	IAA	NA	88	461	NA	32E
8	1AAb+	Male	High risk	(DR3) - DQA1*05 - DQB1*02	DRB1*0401 - DQA1*03 - DQB1*0302	IAA	NA	203	461	NA	32D
8	1AAb+	Male	High risk	(DR3) - DQA1*05 - DQB1*02	DRB1*0401 - DQA1*03 - DQB1*0302	IAA	NA	550	461	NA	32N
8	1AAb+	Male	High risk	(DR3) - DQA1*05 - DQB1*02	DRB1*0401 - DQA1*03 - DQB1*0302	IAA	NA	665	461	NA	32I
8	AAb-	Male	High risk	(DR3) - DQA1*05 - DQB1*02	DRB1*0404 - DQA1*03 - DQB1*0302	NA	NA	105	NA	NA	32A
8	AAb-	Male	High risk	(DR3) - DQA1*05 - DQB1*02	DRB1*0404 - DQA1*03 - DQB1*0302	NA	NA	212	NA	NA	32K
8	AAb-	Male	High risk	(DR3) - DQA1*05 - DQB1*02	DRB1*0404 - DQA1*03 - DQB1*0302	NA	NA	363	NA	NA	32B
8	AAb-	Male	High risk	(DR3) - DQA1*05 - DQB1*02	DRB1*0404 - DQA1*03 - DQB1*0302	NA	NA	597	NA	NA	32G
9	Progressor	Male	Moderately increased risk	DRB1*0401 - DQA1*03 - DQB1*0302	(DR1/10) - DQB1*0501	ICA, IAA, GADA	ICA, IAA, GADA, IA2A	188	398	770	35J
9	Progressor	Male	Moderately increased risk	DRB1*0401 - DQA1*03 - DQB1*0302	(DR1/10) - DQB1*0501	ICA, IAA, GADA	ICA, IAA, GADA, IA2A	574	398	770	35E
9	1AAb+	Male				GADA	NA	186	370	NA	35H
9	1AAb+	Male				GADA	NA	370	370	NA	35B
9	1AAb+	Male				GADA	NA	554	370	NA	35A

9	AAb-	Male		(DR13) - DQB1*0604		NA	NA	184	NA	NA	35D
9	AAb-	Male		(DR13) - DQB1*0604		NA	NA	380	NA	NA	35K
9	AAb-	Male		(DR13) - DQB1*0604		NA	NA	547	NA	NA	35C
10	Progressor	Male	Slightly increased risk	DRB1*0401 - DQA1*03 - DQB1*0302	(DR13) - DQB1*0603	ICA,IAA	ICA, IAA, GADA, IA2A	478	382	572	38E
10	1AAb+	Male	Moderately increased risk	DRB1*0401 - DQA1*03 - DQB1*0302	DRB1*0404 - DQA1*03 - DQB1*0302	GADA	NA	100	100	NA	38B
10	1AAb+	Male	Moderately increased risk	DRB1*0401 - DQA1*03 - DQB1*0302	DRB1*0404 - DQA1*03 - DQB1*0302	GADA	NA	202	100	NA	38I
10	1AAb+	Male	Moderately increased risk	DRB1*0401 - DQA1*03 - DQB1*0302	DRB1*0404 - DQA1*03 - DQB1*0302	GADA	NA	565	100	NA	38D
10	AAb-	Male	Moderately increased risk	DRB1*0401 - DQA1*03 - DQB1*0302	(DR1/10) - DQB1*0501	NA	NA	93	NA	NA	38H
10	AAb-	Male	Moderately increased risk	DRB1*0401 - DQA1*03 - DQB1*0302	(DR1/10) - DQB1*0501	NA	NA	212	NA	NA	38G
10	AAb-	Male	Moderately increased risk	DRB1*0401 - DQA1*03 - DQB1*0302	(DR1/10) - DQB1*0501	NA	NA	543	NA	NA	38A

Supplementary Table S2. LonGP results of the 270 targets identified and quantified with the discovery proteomics. These included 269 endogenous proteins and under the target name "iRT000" the retention time standard peptides.

Target name	Majority Protein ID	Model name	Converge flag	age	sero	t1d	gender	group	pair	id	age* gender	age* group	age*id	noise
IGKC	P01834	model 0 ~ age+gender+ id+age*gender+age*id	2	0.4%	0	0	0.4%	0	0	28.2%	41.1%	0	11.9%	18.1%
IGHG1	P01857	model 0 ~ age+gender+ id+age*gender+age*id	2	0.0%	0	0	3.1%	0	0	29.8%	35.2%	0	16.5%	15.3%
IGHG2	P01859	model 0 ~ age+gender+ id+age*gender+age*id	2	8.5%	0	0	9.7%	0	0	21.2%	10.1%	0	40.6%	9.9%
IGLC3_IGLC2_ IGLC6	P0CG06; P0CG05; P0CF74	model 0 ~ age+gender+ id+age*gender+age*id	2	0.1%	0	0	2.9%	0	0	25.3%	28.1%	0	21.2%	22.4%
IGHM	P01871	model 0 ~ age+group+ id+age*group+age*id	2	24.2%	0	0	0	9.8%	0	36.3%	0	4.2%	10.1%	15.4%
CD5L	O43866	model 0 ~ age+id+age*id	2	26.5%	0	0	0	0	0	29.7%	0	0	25.1%	18.6%
VNN1	O95497	model 0 ~ age+id+age*id	2	12.3%	0	0	0	0	0	67.7%	0	0	14.8%	5.2%
CFD	P00746	model 0 ~ age+id+age*id	2	53.8%	0	0	0	0	0	21.8%	0	0	9.4%	15.0%
P01617_P01614_ P06309_P06310	P01617; P01614 ;P06309; P06310	model 0 ~ age+id+age*id	2	1.0%	0	0	0	0	0	31.5%	0	0	64.9%	2.6%
IGHG4	P01861	model 0 ~ age+id+age*id	2	23.6%	0	0	0	0	0	40.6%	0	0	34.0%	1.7%
IGHA1_IGHA2	P01876;P01877	model 0 ~ age+id+age*id	2	34.1%	0	0	0	0	0	21.3%	0	0	38.8%	5.8%
APOA1	P02647	model 0 ~ age+id+age*id	2	8.1%	0	0	0	0	0	7.5%	0	0	83.1%	1.3%
C1QC	P02747	model 0 ~ age+id+age*id	2	2.4%	0	0	0	0	0	44.5%	0	0	52.3%	0.8%
C9	P02748	model 0 ~ age+id+age*id	2	15.6%	0	0	0	0	0	28.2%	0	0	51.4%	4.8%
PROC	P04070	model 0 ~ age+id+age*id	2	16.9%	0	0	0	0	0	32.2%	0	0	37.0%	13.9%
APOB	P04114	model 0 ~ age+id+age*id	2	7.4%	0	0	0	0	0	14.7%	0	0	64.5%	13.3%
APOD	P05090	model 0 ~ age+id+age*id	2	18.1%	0	0	0	0	0	33.4%	0	0	34.1%	14.4%
HLA_A	P05534; P30447; P13746; P04439 ;P30455 ;P30443; P16188	model 0 ~ age+id+age*id	2	0.4%	0	0	0	0	0	41.1%	0	0	54.2%	4.2%
CTSD	P07339	model 0 ~ age+id+age*id	2	20.0%	0	0	0	0	0	9.3%	0	0	56.1%	14.6%

GP1BA	P07359	model 0 ~ age+id+age*id	2	31.9%	0	0	0	0	0	31.2%	0	0	21.2%	15.7%
C8G	P07360	model 0 ~ age+id+age*id	2	15.0%	0	0	0	0	0	64.3%	0	0	6.1%	14.7%
COL1A2	P08123	model 0 ~ age+id+age*id	2	40.2%	0	0	0	0	0	11.2%	0	0	46.1%	2.5%
MMP2	P08253	model 0 ~ age+id+age*id	2	36.4%	0	0	0	0	0	17.8%	0	0	18.9%	26.9%
DBH	P09172	model 0 ~ age+id+age*id	2	29.1%	0	0	0	0	0	64.4%	0	0	4.7%	1.8%
COL6A1	P12109	model 0 ~ age+id+age*id	2	51.0%	0	0	0	0	0	15.0%	0	0	21.7%	12.4%
COL6A3	P12111	model 0 ~ age+id+age*id	2	50.7%	0	0	0	0	0	20.3%	0	0	16.8%	12.1%
F5	P12259	model 0 ~ age+id+age*id	2	4.8%	0	0	0	0	0	61.1%	0	0	15.6%	18.5%
CDH1	P12830	model 0 ~ age+id+age*id	2	14.3%	0	0	0	0	0	25.4%	0	0	43.5%	16.8%
COL11A2	P13942	model 0 ~ age+id+age*id	2	32.1%	0	0	0	0	0	14.4%	0	0	32.1%	21.4%
CD44	P16070	model 0 ~ age+id+age*id	2	18.0%	0	0	0	0	0	36.7%	0	0	29.4%	15.9%
ACAN	P16112	model 0 ~ age+id+age*id	2	49.0%	0	0	0	0	0	20.2%	0	0	21.1%	9.6%
VCAM1	P19320	model 0 ~ age+id+age*id	2	20.7%	0	0	0	0	0	52.1%	0	0	22.5%	4.7%
PZP	P20742	model 0 ~ age+id+age*id	2	6.0%	0	0	0	0	0	61.5%	0	0	19.0%	13.5%
TNXB	P22105	model 0 ~ age+id+age*id	2	15.7%	0	0	0	0	0	57.2%	0	0	13.6%	13.4%
PROZ	P22891	model 0 ~ age+id+age*id	2	20.0%	0	0	0	0	0	56.1%	0	0	21.7%	2.2%
FBLN1	P23142	model 0 ~ age+id+age*id	2	21.4%	0	0	0	0	0	34.8%	0	0	27.3%	16.4%
PTPRG	P23470	model 0 ~ age+id+age*id	2	32.7%	0	0	0	0	0	30.0%	0	0	15.3%	21.9%
TNC	P24821	model 0 ~ age+id+age*id	2	47.8%	0	0	0	0	0	25.2%	0	0	17.5%	9.5%
SERPINF1	P36955	model 0 ~ age+id+age*id	2	14.8%	0	0	0	0	0	63.7%	0	0	10.0%	11.6%
AFM	P43652	model 0 ~ age+id+age*id	2	13.1%	0	0	0	0	0	62.7%	0	0	10.8%	13.4%
NAGLU	P54802	model 0 ~ age+id+age*id	2	18.7%	0	0	0	0	0	61.7%	0	0	6.7%	12.9%
APOC4	P55056	model 0 ~ age+id+age*id	2	32.6%	0	0	0	0	0	20.5%	0	0	36.5%	10.4%
LYZ	P61626	model 0 ~ age+id+age*id	2	5.8%	0	0	0	0	0	66.0%	0	0	19.3%	8.9%
HSPG2	P98160	model 0 ~ age+id+age*id	2	8.6%	0	0	0	0	0	52.7%	0	0	21.3%	17.4%
ITIH3	Q06033	model 0 ~ age+id+age*id	2	0.8%	0	0	0	0	0	33.0%	0	0	65.3%	0.8%
LGALS3BP	Q08380	model 0 ~ age+id+age*id	2	29.1%	0	0	0	0	0	29.4%	0	0	34.2%	7.3%
SPP2	Q13103	model 0 ~ age+id+age*id	2	15.8%	0	0	0	0	0	41.9%	0	0	31.6%	10.8%
DSG2	Q14126	model 0 ~ age+id+age*id	2	60.6%	0	0	0	0	0	9.9%	0	0	14.4%	15.0%
FGL2	Q14314	model 0 ~ age+id+age*id	2	11.6%	0	0	0	0	0	54.9%	0	0	18.5%	15.0%
POSTN	Q15063	model 0 ~ age+id+age*id	2	57.3%	0	0	0	0	0	22.9%	0	0	13.3%	6.4%

TGFBI	Q15582	model 0 ~ age+id+age*id	2	26.0%	0	0	0	0	0	56.8%	0	0	12.9%	4.4%
ADIPOQ	Q15848	model 0 ~ age+id+age*id	2	29.1%	0	0	0	0	0	59.9%	0	0	5.0%	6.0%
AOC3	Q16853	model 0 ~ age+id+age*id	2	9.0%	0	0	0	0	0	46.8%	0	0	33.5%	10.7%
VASN	Q6EMK4	model 0 ~ age+id+age*id	2	52.4%	0	0	0	0	0	21.5%	0	0	9.9%	16.3%
PI16	Q6UXB8	model 0 ~ age+id+age*id	2	66.1%	0	0	0	0	0	24.2%	0	0	5.7%	3.9%
CD109	Q6YHK3	model 0 ~ age+id+age*id	2	9.8%	0	0	0	0	0	67.5%	0	0	8.5%	14.3%
CD163	Q86VB7	model 0 ~ age+id+age*id	2	1.9%	0	0	0	0	0	74.4%	0	0	18.5%	5.2%
CFHR4	Q92496	model 0 ~ age+id+age*id	2	10.9%	0	0	0	0	0	83.7%	0	0	5.2%	0.2%
GGH	Q92820	model 0 ~ age+id+age*id	2	48.6%	0	0	0	0	0	34.5%	0	0	13.7%	3.2%
CPB2	Q96IY4	model 0 ~ age+id+age*id	2	13.5%	0	0	0	0	0	50.1%	0	0	26.8%	9.6%
CNDP1	Q96KN2	model 0 ~ age+id+age*id	2	46.4%	0	0	0	0	0	49.2%	0	0	3.6%	0.7%
IL1RAP	Q9NPH3	model 0 ~ age+id+age*id	2	17.3%	0	0	0	0	0	70.1%	0	0	6.1%	6.4%
CD93	Q9NPY3	model 0 ~ age+id+age*id	2	30.5%	0	0	0	0	0	44.0%	0	0	18.8%	6.7%
C1RL	Q9NZP8	model 0 ~ age+id+age*id	2	4.0%	0	0	0	0	0	4.3%	0	0	90.5%	1.2%
PCYOX1	Q9UHG3	model 0 ~ age+id+age*id	2	27.1%	0	0	0	0	0	3.3%	0	0	61.4%	8.2%
SERPINA10	Q9UK55	model 0 ~ age+id+age*id	2	3.6%	0	0	0	0	0	86.7%	0	0	6.7%	3.0%
PROCR	Q9UNN8	model 0 ~ age+id+age*id	2	14.7%	0	0	0	0	0	76.7%	0	0	2.2%	6.5%
ALB	P02768-1; P02768	model 0 ~ age+pair+id+age*id	2	16.9%	0	0	0	0	29.2%	7.0%	0	0	16.8%	30.2%
NRP1	O14786	model 0 ~ age+pair+id+age*id	2	10.5%	0	0	0	0	52.2%	4.5%	0	0	16.9%	16.0%
APOL1	O14791	model 0 ~ age+pair+id+age*id	2	11.0%	0	0	0	0	26.9%	4.6%	0	0	40.8%	16.7%
ATRN	O75882	model 0 ~ age+pair+id+age*id	2	8.4%	0	0	0	0	40.8%	7.0%	0	0	32.1%	11.7%
CP	P00450	model 0 ~ age+pair+id+age*id	2	26.1%	0	0	0	0	44.2%	1.7%	0	0	22.1%	5.9%
F13A1	P00488	model 0 ~ age+pair+id+age*id	2	3.7%	0	0	0	0	26.2%	1.7%	0	0	45.4%	23.0%
PLG	P00747	model 0 ~ age+pair+id+age*id	2	16.9%	0	0	0	0	61.5%	1.5%	0	0	18.3%	1.9%
CFB	P00751	model 0 ~ age+pair+id+age*id	2	4.5%	0	0	0	0	49.4%	2.3%	0	0	38.6%	5.3%
AGT	P01019	model 0 ~ age+pair+id+age*id	2	38.5%	0	0	0	0	47.2%	3.4%	0	0	7.0%	3.9%
CST3	P01034	model 0 ~ age+pair+id+age*id	2	25.4%	0	0	0	0	54.1%	4.1%	0	0	7.3%	9.1%
IGHG3	P01860	model 0 ~ age+pair+id+age*id	2	5.8%	0	0	0	0	32.4%	24.1%	0	0	15.8%	21.9%
COL1A1	P02452	model 0 ~ age+pair+id+age*id	2	70.9%	0	0	0	0	11.1%	4.7%	0	0	7.0%	6.3%
COL2A1	P02458	model 0 ~ age+pair+id+age*id	2	40.0%	0	0	0	0	26.4%	3.5%	0	0	28.6%	1.5%
APOA2	P02652	model 0 ~ age+pair+id+age*id	2	18.8%	0	0	0	0	10.7%	1.2%	0	0	66.8%	2.5%

C1QA	P02745	model 0 ~ age+pair+id+age*id	2	3.4%	0	0	0	0	32.4%	17.9%	0	0	44.5%	1.8%
C4BPA	P04003	model 0 ~ age+pair+id+age*id	2	15.9%	0	0	0	0	26.5%	27.3%	0	0	19.6%	10.8%
VTN	P04004	model 0 ~ age+pair+id+age*id	2	9.7%	0	0	0	0	34.1%	2.6%	0	0	37.8%	15.7%
CFI	P05156	model 0 ~ age+pair+id+age*id	2	17.9%	0	0	0	0	56.6%	0.2%	0	0	20.2%	5.1%
SERPINA7	P05543	model 0 ~ age+pair+id+age*id	2	31.0%	0	0	0	0	45.9%	7.0%	0	0	12.2%	3.9%
ITGB1	P05556	model 0 ~ age+pair+id+age*id	2	14.7%	0	0	0	0	38.0%	1.3%	0	0	27.0%	18.9%
C2	P06681	model 0 ~ age+pair+id+age*id	2	22.1%	0	0	0	0	52.9%	3.6%	0	0	17.3%	4.1%
C8A	P07357	model 0 ~ age+pair+id+age*id	2	10.1%	0	0	0	0	61.5%	2.0%	0	0	23.2%	3.2%
C8B	P07358	model 0 ~ age+pair+id+age*id	2	16.6%	0	0	0	0	43.2%	9.4%	0	0	20.4%	10.4%
SERPINA6	P08185	model 0 ~ age+pair+id+age*id	2	47.4%	0	0	0	0	22.7%	9.6%	0	0	6.7%	13.6%
C4B	P0C0L5	model 0 ~ age+pair+id+age*id	2	0.2%	0	0	0	0	42.5%	15.8%	0	0	40.4%	1.3%
SPP1	P10451	model 0 ~ age+pair+id+age*id	2	43.7%	0	0	0	0	31.3%	0.9%	0	0	20.0%	4.1%
PEPD	P12955	model 0 ~ age+pair+id+age*id	2	27.6%	0	0	0	0	28.2%	9.6%	0	0	16.3%	18.4%
NCAM1	P13591	model 0 ~ age+pair+id+age*id	2	11.9%	0	0	0	0	46.3%	15.6%	0	0	13.6%	12.6%
C6	P13671	model 0 ~ age+pair+id+age*id	2	3.5%	0	0	0	0	48.0%	5.9%	0	0	40.8%	1.9%
SELL	P14151	model 0 ~ age+pair+id+age*id	2	11.0%	0	0	0	0	44.1%	16.6%	0	0	21.9%	6.4%
ANPEP	P15144	model 0 ~ age+pair+id+age*id	2	6.4%	0	0	0	0	55.6%	5.8%	0	0	17.4%	14.9%
CPN1	P15169	model 0 ~ age+pair+id+age*id	2	6.7%	0	0	0	0	36.6%	42.8%	0	0	9.8%	4.2%
IGFBP3	P17936	model 0 ~ age+pair+id+age*id	2	11.7%	0	0	0	0	36.1%	11.7%	0	0	21.0%	19.5%
GPX3	P22352	model 0 ~ age+pair+id+age*id	2	40.3%	0	0	0	0	22.5%	13.7%	0	0	11.7%	11.7%
PON1	P27169	model 0 ~ age+pair+id+age*id	2	13.0%	0	0	0	0	38.0%	25.3%	0	0	8.9%	14.9%
CFP	P27918	model 0 ~ age+pair+id+age*id	2	17.6%	0	0	0	0	58.2%	0.3%	0	0	11.6%	12.3%
CDH5	P33151	model 0 ~ age+pair+id+age*id	2	24.0%	0	0	0	0	31.7%	21.0%	0	0	15.2%	8.1%
IGFALS	P35858	model 0 ~ age+pair+id+age*id	2	28.7%	0	0	0	0	32.5%	20.3%	0	0	11.4%	7.0%
PTGDS	P41222	model 0 ~ age+pair+id+age*id	2	11.0%	0	0	0	0	68.6%	6.8%	0	0	4.9%	8.7%
SEPP1	P49908	model 0 ~ age+pair+id+age*id	2	26.4%	0	0	0	0	34.1%	0.7%	0	0	36.2%	2.6%
B2M	P61769	model 0 ~ age+pair+id+age*id	2	11.9%	0	0	0	0	38.5%	17.0%	0	0	15.7%	16.9%
GPLD1	P80108	model 0 ~ age+pair+id+age*id	2	18.9%	0	0	0	0	47.7%	6.6%	0	0	15.0%	11.8%
BASP1	P80723	model 0 ~ age+pair+id+age*id	2	21.9%	0	0	0	0	45.9%	8.1%	0	0	12.2%	11.9%
CNTN1	Q12860	model 0 ~ age+pair+id+age*id	2	13.0%	0	0	0	0	20.2%	3.7%	0	0	55.8%	7.3%
FAP	Q12884	model 0 ~ age+pair+id+age*id	2	35.7%	0	0	0	0	12.3%	6.8%	0	0	36.9%	8.3%

HABP2	Q14520	model 0 ~ age+pair+id+age*id	2	11.4%	0	0	0	0	57.4%	3.1%	0	0	23.6%	4.6%
ITIH4	Q14624	model 0 ~ age+pair+id+age*id	2	46.3%	0	0	0	0	20.4%	6.6%	0	0	21.7%	5.0%
PCOLCE	Q15113	model 0 ~ age+pair+id+age*id	2	7.7%	0	0	0	0	33.5%	30.1%	0	0	15.6%	13.2%
PRG4	Q92954	model 0 ~ age+pair+id+age*id	2	15.7%	0	0	0	0	38.7%	17.5%	0	0	19.8%	8.3%
PGLYRP2	Q96PD5	model 0 ~ age+pair+id+age*id	2	17.0%	0	0	0	0	26.8%	7.9%	0	0	25.6%	22.8%
OLFM1	Q99784	model 0 ~ age+pair+id+age*id	2	48.3%	0	0	0	0	18.3%	8.3%	0	0	15.3%	9.8%
FETUB	Q9UGM5	model 0 ~ age+pair+id+age*id	2	33.3%	0	0	0	0	39.4%	7.2%	0	0	17.0%	3.0%
LPA	P08519	model 0 ~ age+sero+id+age*id	2	8.7%	4.3%	0	0	0	0	82.2%	0	0	3.5%	1.2%
IGFBP2	P18065	model 0 ~ age+sero+t1d+id+age*id	2	47.7%	1.7%	0.9%	0	0	0	11.9%	0	0	22.5%	15.4%
CPN2	P22792	model 0 ~ age+t1d+id+age*id	2	13.7%	0	0.1%	0	0	0	42.4%	0	0	34.2%	9.5%
C1QB	P02746	model 0 ~ age+t1d+pair+id+age*id	2	1.0%	0	2.1%	0	0	31.5%	22.0%	0	0	42.1%	1.4%
A2M	P01023	model 0 ~ group+pair+id	2	0	0	0	0	7.8%	45.9%	6.6%	0	0	0	39.7%
IGLL5_IGLC1	B9A064;P0CG04	model 0 ~ id	2	0	0	0	0	0	0	12.4%	0	0	0	87.6%
QSOX1	O00391	model 0 ~ id	2	0	0	0	0	0	0	56.2%	0	0	0	43.8%
CHL1	O00533	model 0 ~ id	2	0	0	0	0	0	0	46.2%	0	0	0	53.8%
APOM	O95445	model 0 ~ id	2	0	0	0	0	0	0	2.2%	0	0	0	97.8%
LDHA	P00338	model 0 ~ id	2	0	0	0	0	0	0	49.2%	0	0	0	50.8%
HP	P00738	model 0 ~ id	2	0	0	0	0	0	0	3.5%	0	0	0	96.5%
HPR	P00739	model 0 ~ id	2	0	0	0	0	0	0	53.9%	0	0	0	46.1%
SERPINA3	P01011	model 0 ~ id	2	0	0	0	0	0	0	29.8%	0	0	0	70.2%
IGF2	P01344	model 0 ~ id	2	0	0	0	0	0	0	28.1%	0	0	0	71.9%
APOE	P02649	model 0 ~ id	2	0	0	0	0	0	0	45.5%	0	0	0	54.5%
APOC2	P02655	model 0 ~ id	2	0	0	0	0	0	0	18.5%	0	0	0	81.5%
FGA	P02671	model 0 ~ id	2	0	0	0	0	0	0	4.3%	0	0	0	95.7%
APOH	P02749	model 0 ~ id	2	0	0	0	0	0	0	49.1%	0	0	0	50.9%
FN1	P02751	model 0 ~ id	2	0	0	0	0	0	0	20.1%	0	0	0	79.9%
ORM1	P02763	model 0 ~ id	2	0	0	0	0	0	0	8.6%	0	0	0	91.4%
PPBP	P02775	model 0 ~ id	2	0	0	0	0	0	0	1.1%	0	0	0	98.9%
HRG	P04196	model 0 ~ id	2	0	0	0	0	0	0	42.2%	0	0	0	57.8%
VWF	P04275	model 0 ~ id	2	0	0	0	0	0	0	29.3%	0	0	0	70.7%
SHBG	P04278	model 0 ~ id	2	0	0	0	0	0	0	45.8%	0	0	0	54.2%

ALDOB	P05062	model 0 ~ id	2	0	0	0	0	0	0	4.1%	0	0	0	95.9%
SERPINA5	P05154	model 0 ~ id	2	0	0	0	0	0	0	13.3%	0	0	0	86.7%
ICAM1	P05362	model 0 ~ id	2	0	0	0	0	0	0	45.1%	0	0	0	54.9%
CLEC3B	P05452	model 0 ~ id	2	0	0	0	0	0	0	23.6%	0	0	0	76.4%
BCHE	P06276	model 0 ~ id	2	0	0	0	0	0	0	72.0%	0	0	0	28.0%
GSN	P06396	model 0 ~ id	2	0	0	0	0	0	0	49.7%	0	0	0	50.3%
CSF1R	P07333	model 0 ~ id	2	0	0	0	0	0	0	29.3%	0	0	0	70.7%
THBS1	P07996	model 0 ~ id	2	0	0	0	0	0	0	18.0%	0	0	0	82.0%
SOD3	P08294	model 0 ~ id	2	0	0	0	0	0	0	69.4%	0	0	0	30.6%
SERPINF2	P08697	model 0 ~ id	2	0	0	0	0	0	0	12.7%	0	0	0	87.3%
F7	P08709	model 0 ~ id	2	0	0	0	0	0	0	19.4%	0	0	0	80.6%
SAA1	P0DJI8	model 0 ~ id	2	0	0	0	0	0	0	1.4%	0	0	0	98.6%
MBL2	P11226	model 0 ~ id	2	0	0	0	0	0	0	96.3%	0	0	0	3.7%
FGFR1	P11362	model 0 ~ id	2	0	0	0	0	0	0	6.3%	0	0	0	93.7%
CETP	P11597	model 0 ~ id	2	0	0	0	0	0	0	19.6%	0	0	0	80.4%
ACTN1_ ACTN4	P12814;O43707	model 0 ~ id	2	0	0	0	0	0	0	1.6%	0	0	0	98.4%
PRG2	P13727	model 0 ~ id	2	0	0	0	0	0	0	19.2%	0	0	0	80.8%
LCP1	P13796	model 0 ~ id	2	0	0	0	0	0	0	49.7%	0	0	0	50.3%
PVR	P15151	model 0 ~ id	2	0	0	0	0	0	0	48.9%	0	0	0	51.1%
VCL	P18206	model 0 ~ id	2	0	0	0	0	0	0	14.6%	0	0	0	85.4%
ORM2	P19652	model 0 ~ id	2	0	0	0	0	0	0	15.3%	0	0	0	84.7%
FLNA	P21333	model 0 ~ id	2	0	0	0	0	0	0	2.8%	0	0	0	97.2%
ATP5A1	P25705	model 0 ~ id	2	0	0	0	0	0	0	44.8%	0	0	0	55.2%
MST1	P26927	model 0 ~ id	2	0	0	0	0	0	0	87.4%	0	0	0	12.6%
DPP4	P27487	model 0 ~ id	2	0	0	0	0	0	0	27.5%	0	0	0	72.5%
SERPINA4	P29622	model 0 ~ id	2	0	0	0	0	0	0	61.3%	0	0	0	38.7%
PRDX2	P32119	model 0 ~ id	2	0	0	0	0	0	0	2.5%	0	0	0	97.5%
SAA4	P35542	model 0 ~ id	2	0	0	0	0	0	0	12.2%	0	0	0	87.8%
KRT2	P35908	model 0 ~ id	2	0	0	0	0	0	0	5.9%	0	0	0	94.1%
CFHR2	P36980	model 0 ~ id	2	0	0	0	0	0	0	88.1%	0	0	0	11.9%
MASP1	P48740	model 0 ~ id	2	0	0	0	0	0	0	43.6%	0	0	0	56.4%

PLTP	P55058	model 0 ~ id	2	0	0	0	0	0	0	20.8%	0	0	0	79.2%
CDH13	P55290	model 0 ~ id	2	0	0	0	0	0	0	62.8%	0	0	0	37.2%
ACTG1_ ACTB	P63261;P60709	model 0 ~ id	2	0	0	0	0	0	0	7.9%	0	0	0	92.1%
TMSB4X	P62328	model 0 ~ id	2	0	0	0	0	0	0	4.8%	0	0	0	95.2%
YWHAZ	P63104	model 0 ~ id	2	0	0	0	0	0	0	23.0%	0	0	0	77.0%
TPM4	P67936	model 0 ~ id	2	0	0	0	0	0	0	8.4%	0	0	0	91.6%
HBB	P68871	model 0 ~ id	2	0	0	0	0	0	0	5.2%	0	0	0	94.8%
HBA1	P69905	model 0 ~ id	2	0	0	0	0	0	0	4.2%	0	0	0	95.8%
CTBS	Q01459	model 0 ~ id	2	0	0	0	0	0	0	53.7%	0	0	0	46.3%
CFHR1	Q03591	model 0 ~ id	2	0	0	0	0	0	0	84.2%	0	0	0	15.8%
HGFAC	Q04756	model 0 ~ id	2	0	0	0	0	0	0	60.1%	0	0	0	39.9%
BST1	Q10588	model 0 ~ id	2	0	0	0	0	0	0	78.7%	0	0	0	21.3%
PTPRJ	Q12913	model 0 ~ id	2	0	0	0	0	0	0	54.6%	0	0	0	45.4%
ENPP2	Q13822	model 0 ~ id	2	0	0	0	0	0	0	23.8%	0	0	0	76.2%
SPARCL1	Q14515	model 0 ~ id	2	0	0	0	0	0	0	39.7%	0	0	0	60.3%
FCN2	Q15485	model 0 ~ id	2	0	0	0	0	0	0	71.4%	0	0	0	28.6%
ECM1	Q16610	model 0 ~ id	2	0	0	0	0	0	0	35.9%	0	0	0	64.1%
ADAMTS13	Q76LX8	model 0 ~ id	2	0	0	0	0	0	0	25.4%	0	0	0	74.6%
SERPINA11	Q86U17	model 0 ~ id	2	0	0	0	0	0	0	19.7%	0	0	0	80.3%
CILP2	Q8IUL8	model 0 ~ id	2	0	0	0	0	0	0	17.4%	0	0	0	82.6%
CFHR5	Q9BXR6	model 0 ~ id	2	0	0	0	0	0	0	5.4%	0	0	0	94.6%
DPEP2	Q9H4A9	model 0 ~ id	2	0	0	0	0	0	0	25.7%	0	0	0	74.3%
APMAP	Q9HDC9	model 0 ~ id	2	0	0	0	0	0	0	7.2%	0	0	0	92.8%
CRTAC1	Q9NQ79	model 0 ~ id	2	0	0	0	0	0	0	36.8%	0	0	0	63.2%
TLN1	Q9Y490	model 0 ~ id	2	0	0	0	0	0	0	5.5%	0	0	0	94.5%
LYVE1	Q9Y5Y7	model 0 ~ id	2	0	0	0	0	0	0	50.2%	0	0	0	49.8%
KRT10	P13645	model 0 ~ pair+id	2	0	0	0	0	0	19.9%	3.2%	0	0	0	76.9%
KRT9	P35527	model 0 ~ pair+id	2	0	0	0	0	0	10.2%	1.8%	0	0	0	88.0%
iRT000	iRT000	model 0 ~ pair+id	2	0	0	0	0	0	63.8%	0.5%	0	0	0	35.7%
MASP2	O00187	model 0 ~ pair+id	2	0	0	0	0	0	25.0%	20.1%	0	0	0	54.9%
FCN3	O75636	model 0 ~ pair+id	2	0	0	0	0	0	18.1%	7.7%	0	0	0	74.2%

F2	P00734	model 0 ~ pair+id	2	0	0	0	0	0	22.4%	9.3%	0	0	0	68.3%
C1R	P00736	model 0 ~ pair+id	2	0	0	0	0	0	23.5%	8.5%	0	0	0	67.9%
F9	P00740	model 0 ~ pair+id	2	0	0	0	0	0	43.6%	1.1%	0	0	0	55.4%
F10	P00742	model 0 ~ pair+id	2	0	0	0	0	0	50.3%	13.1%	0	0	0	36.6%
SERPINC1	P01008	model 0 ~ pair+id	2	0	0	0	0	0	48.3%	3.6%	0	0	0	48.0%
SERPINA1	P01009	model 0 ~ pair+id	2	0	0	0	0	0	53.7%	7.1%	0	0	0	39.1%
C3	P01024	model 0 ~ pair+id	2	0	0	0	0	0	29.1%	6.8%	0	0	0	64.0%
C5	P01031	model 0 ~ pair+id	2	0	0	0	0	0	51.7%	1.2%	0	0	0	47.1%
KNG1	P01042	model 0 ~ pair+id	2	0	0	0	0	0	39.0%	3.5%	0	0	0	57.6%
APOC3	P02656	model 0 ~ pair+id	2	0	0	0	0	0	31.6%	1.7%	0	0	0	66.7%
APCS	P02743	model 0 ~ pair+id	2	0	0	0	0	0	39.8%	1.1%	0	0	0	59.2%
LRG1	P02750	model 0 ~ pair+id	2	0	0	0	0	0	29.2%	2.1%	0	0	0	68.7%
RBP4	P02753	model 0 ~ pair+id	2	0	0	0	0	0	50.6%	5.5%	0	0	0	43.8%
AMBP	P02760	model 0 ~ pair+id	2	0	0	0	0	0	65.0%	3.5%	0	0	0	31.6%
TTR	P02766	model 0 ~ pair+id	2	0	0	0	0	0	39.3%	0.9%	0	0	0	59.9%
HPX	P02790	model 0 ~ pair+id	2	0	0	0	0	0	34.5%	10.5%	0	0	0	55.0%
F11	P03951	model 0 ~ pair+id	2	0	0	0	0	0	28.3%	27.5%	0	0	0	44.3%
KLKB1	P03952	model 0 ~ pair+id	2	0	0	0	0	0	37.5%	5.2%	0	0	0	57.3%
LCAT	P04180	model 0 ~ pair+id	2	0	0	0	0	0	60.8%	1.4%	0	0	0	37.8%
A1BG	P04217	model 0 ~ pair+id	2	0	0	0	0	0	49.9%	7.7%	0	0	0	42.4%
KRT1	P04264	model 0 ~ pair+id	2	0	0	0	0	0	14.0%	1.4%	0	0	0	84.6%
SERPING1	P05155	model 0 ~ pair+id	2	0	0	0	0	0	43.7%	11.1%	0	0	0	45.1%
F13B	P05160	model 0 ~ pair+id	2	0	0	0	0	0	10.7%	1.0%	0	0	0	88.3%
SERPIND1	P05546	model 0 ~ pair+id	2	0	0	0	0	0	50.1%	8.4%	0	0	0	41.5%
LDHB	P07195	model 0 ~ pair+id	2	0	0	0	0	0	33.0%	21.8%	0	0	0	45.2%
PROS1	P07225	model 0 ~ pair+id	2	0	0	0	0	0	34.6%	10.7%	0	0	0	54.7%
SLC3A2	P08195	model 0 ~ pair+id	2	0	0	0	0	0	62.7%	0.3%	0	0	0	37.0%
CD14	P08571	model 0 ~ pair+id	2	0	0	0	0	0	37.8%	13.4%	0	0	0	48.8%
CFH	P08603	model 0 ~ pair+id	2	0	0	0	0	0	56.1%	1.3%	0	0	0	42.5%
C1S	P09871	model 0 ~ pair+id	2	0	0	0	0	0	44.2%	4.4%	0	0	0	51.5%
C4A	P0C0L4	model 0 ~ pair+id	2	0	0	0	0	0	47.2%	8.1%	0	0	0	44.7%

C7	P10643	model 0 ~ pair+id	2	0	0	0	0	0	34.4%	43.2%	0	0	0	22.4%
CLU	P10909	model 0 ~ pair+id	2	0	0	0	0	0	30.6%	1.5%	0	0	0	67.9%
HSPA5	P11021	model 0 ~ pair+id	2	0	0	0	0	0	31.3%	3.3%	0	0	0	65.3%
ICAM2	P13598	model 0 ~ pair+id	2	0	0	0	0	0	22.1%	0.9%	0	0	0	77.0%
LBP	P18428	model 0 ~ pair+id	2	0	0	0	0	0	22.8%	4.2%	0	0	0	73.1%
ITIH2	P19823	model 0 ~ pair+id	2	0	0	0	0	0	38.4%	10.2%	0	0	0	51.4%
ITIH1	P19827	model 0 ~ pair+id	2	0	0	0	0	0	47.1%	8.0%	0	0	0	44.9%
AZGP1	P25311	model 0 ~ pair+id	2	0	0	0	0	0	51.8%	6.3%	0	0	0	41.9%
MAN1A1	P33908	model 0 ~ pair+id	2	0	0	0	0	0	39.5%	2.9%	0	0	0	57.5%
MDH2	P40926	model 0 ~ pair+id	2	0	0	0	0	0	68.5%	4.6%	0	0	0	26.8%
BTD	P43251	model 0 ~ pair+id	2	0	0	0	0	0	37.0%	39.0%	0	0	0	24.0%
LUM	P51884	model 0 ~ pair+id	2	0	0	0	0	0	35.3%	13.1%	0	0	0	51.5%
EFEMP1	Q12805	model 0 ~ pair+id	2	0	0	0	0	0	34.9%	9.4%	0	0	0	55.7%
APOF	Q13790	model 0 ~ pair+id	2	0	0	0	0	0	52.9%	1.8%	0	0	0	45.3%
PLXDC2	Q6UX71	model 0 ~ pair+id	2	0	0	0	0	0	47.2%	5.5%	0	0	0	47.3%
ABI3BP	Q7Z7G0	model 0 ~ pair+id	2	0	0	0	0	0	24.7%	14.1%	0	0	0	61.2%
LILRA3	Q8N6C8	model 0 ~ pair+id	2	0	0	0	0	0	34.4%	11.9%	0	0	0	53.7%
MINPP1	Q9UNW1	model 0 ~ pair+id	2	0	0	0	0	0	51.8%	2.5%	0	0	0	45.7%
F12	P00748	model 0 ~ sero+id	2	0	1.2%	0	0	0	0	91.7%	0	0	0	7.1%
FGG	P02679	model 0 ~ sero+id	2	0	0.2%	0	0	0	0	4.6%	0	0	0	95.2%
GC	P02774	model 0 ~ sero+id	2	0	4.4%	0	0	0	0	42.1%	0	0	0	53.5%
ALDOA	P04075	model 0 ~ sero+id	2	0	3.4%	0	0	0	0	34.9%	0	0	0	61.7%
C4BPB	P20851	model 0 ~ sero+id	2	0	6.0%	0	0	0	0	51.1%	0	0	0	42.9%
THBS4	P35443	model 0 ~ sero+id	2	0	4.9%	0	0	0	0	25.4%	0	0	0	69.7%
COMP	P49747	model 0 ~ sero+id	2	0	2.0%	0	0	0	0	54.6%	0	0	0	43.4%
LRP1	Q07954	model 0 ~ sero+id	2	0	21.4%	0	0	0	0	43.8%	0	0	0	34.7%
IGJ	P01591	model 0 ~ sero+pair+id	2	0	16.1%	0	0	0	18.8%	5.7%	0	0	0	59.3%
FGB	P02675	model 0 ~ sero+pair+id	2	0	1.0%	0	0	0	10.3%	1.5%	0	0	0	87.2%
AHSG	P02765	model 0 ~ sero+pair+id	2	0	2.3%	0	0	0	53.7%	7.0%	0	0	0	37.0%
TF	P02787	model 0 ~ sero+pair+id	2	0	1.4%	0	0	0	70.7%	4.0%	0	0	0	23.9%
APOA4	P06727	model 0 ~ sero+pair+id	2	0	15.2%	0	0	0	15.6%	3.7%	0	0	0	65.4%

MCAM	P43121	model 0 ~ sero+pair+id	2	0	2.9%	0	0	0	44.1%	2.1%	0	0	0	50.9%
CRISP3	P54108	model 0 ~ t1d+id	2	0	0	3.9%	0	0	0	31.9%	0	0	0	64.2%
APOC1	P02654	model 0 ~ t1d+pair+id	2	0	0	2.1%	0	0	51.2%	6.5%	0	0	0	40.2%

Supplementary Table S3. The 115 proteins that showed age-associated changes in the discovery results using LonGP modelling. The last four columns indicate whether the protein was also reported with age-associated changes (regardless of the trend) in previous pediatric serum/plasma studies from Liu et al., Bjelosevic et al., Lietzén et al., or in Mikus et al., respectively.

Gene name	Majority Protein ID	Liu et al. J Proteomics. 2017	Bjelosevic et al. MCP. 2017	Lietzén et al. Sci Rep. 2018	Mikus M et al. Proteomics Clin Appl. 2021
APOA1	P02647	x			x
APOB	P04114	x			
DBH	P09172	x		x	
CNDP1	Q96KN2	x			
CPB2	Q96IY4	x	x	x	
C8B	P07358	x	x	x	
SERPINA6	P08185	x	x	x	
PEPD	P12955				
ITIH4	Q14624		x	x	
FETUB	Q9UGM5	x		x	x
APOC4	P55056			x	
IGFALS	P35858	x	x	x	
IGHA1;IGHA2	P01876;P01877		x		
PROZ	P22891				
GPX3	P22352	x		x	
HABP2	Q14520	x		x	
C8G	P07360		x	x	
SEPP1	P49908				
CFI	P05156		x	x	
PLG	P00747	x	x	x	
CFP	P27918	x		x	
CP	P00450	x	x	x	
SERPINA10	Q9UK55			x	
C6	P13671	x	x		
CD163	Q86VB7	x			x
PRG4	Q92954			x	
LPA	P08519	x			x
CFHR4	Q92496	x		x	
C9	P02748	x	x	x	x
IGHG2	P01859		x	x	
IGHM	P01871		x		
CD5L	O43866	x	x		
C1RL	Q9NZP8			x	
VTN	P04004	x	x		
APOD	P05090		x		
C1QC	P02747	x			
C1QA	P02745	x			
APOA2	P02652	x	x		
IGHG4	P01861		x	x	

IGFBP3	P17936	x			
C8A	P07357		x		
C4BPA	P04003	x	x	x	
PCYOX1	Q9UHG3	x		x	
PON1	P27169	x	x	x	
CFB	P00751		x		
IL1RAP	Q9NPH3	x			
APOL1	O14791	x		x	
PROC	P04070				
SELL	P14151	x			
IGHG3	P01860		x		
COL1A2	P08123	x			
POSTN	Q15063			x	
PI16	Q6UXB8	x		x	
COL1A1	P02452	x	x	x	
TNC	P24821	x		x	
GGH	Q92820	x		x	
VASN	Q6EMK4	x		x	
AGT	P01019		x	x	
DSG2	Q14126	x			
COL2A1	P02458	x			
ACAN	P16112	x		x	
COL11A2	P13942	x			
FGL2	Q14314	x			
CTSD	P07339	x			
SERPINA7	P05543		x	x	
ADIPOQ	Q15848	x		x	
PTPRG	P23470	x		x	
HSPG2	P98160			x	
AFM	P43652	x		x	
C2	P06681			x	
FBLN1	P23142	x		x	
CST3	P01034	x		x	
SPP1	P10451				x
BASP1	P80723	x			
OLFM1	Q99784	x		x	
IGFBP2	P18065	x		x	
COL6A3	P12111	x		x	
COL6A1	P12109	x		x	
CFD	P00746	x		x	
PTGDS	P41222	x		x	
MMP2	P08253	x		x	
LGALS3BP	Q08380	x		x	
VCAM1	P19320	x		x	
FAP	Q12884				
CDH5	P33151		x	x	
CD93	Q9NPY3				

CNTN1	Q12860	x			
TNXB	P22105	x		x	
TGFBI	Q15582	x		x	
GPLD1	P80108	x		x	
CDH1	P12830	x		x	
NRP1	O14786	x			
CPN2	P22792	x	x	x	
SPP2	Q13103	x		x	
PZP	P20742	x			
CD109	Q6YHK3	x			
LYZ	P61626			x	
F13A1	P00488		x		
PCOLCE	Q15113			x	
PGLYRP2	Q96PD5	x	x	x	
AOC3	Q16853	x			
VNN1	O95497	x			
F5	P12259	x		x	
ITGB1	P05556	x			
CPN1	P15169	x		x	
ANPEP	P15144	x		x	
ATRN	O75882			x	
PROCR	Q9UNN8	x		x	
NAGLU	P54802	x			
SERPINF1	P36955	x	x	x	
CD44	P16070	x		x	
B2M	P61769	x		x	
NCAM1	P13591	x		x	
GP1BA	P07359	x		x	
ALB	P02768	x		x	

Supplementary Table S4. Summary and serum sample collection of children selected for the targeted proteomics analysis.

Pair	Status	Sex	HLA risk group	HLA haplotype 1	HLA haplotype 2	First autoantibody	Overall autoantibodies detected	Age at sample collection (days)	Seroconversion age (days)	Diagnosis age (days)	Batch	SRM code
11	AAb-	Male	High risk	(DR3) - DQA1*05 - DQB1*02	DRB1*0404 - DQA1*03 - DQB1*0302	NA	NA	99	NA	NA	Batch 1	11B
11	AAb-	Male	High risk	(DR3) - DQA1*05 - DQB1*02	DRB1*0404 - DQA1*03 - DQB1*0302	NA	NA	197	NA	NA	Batch 1	11Er
11	AAb-	Male	High risk	(DR3) - DQA1*05 - DQB1*02	DRB1*0404 - DQA1*03 - DQB1*0302	NA	NA	307	NA	NA	Batch 1	11Or
11	AAb-	Male	High risk	(DR3) - DQA1*05 - DQB1*02	DRB1*0404 - DQA1*03 - DQB1*0302	NA	NA	393	NA	NA	Batch 1	11Rr
11	AAb-	Male	High risk	(DR3) - DQA1*05 - DQB1*02	DRB1*0404 - DQA1*03 - DQB1*0302	NA	NA	476	NA	NA	Batch 1	11G
11	AAb-	Male	High risk	(DR3) - DQA1*05 - DQB1*02	DRB1*0404 - DQA1*03 - DQB1*0302	NA	NA	573	NA	NA	Batch 1	11Dr
11	AAb-	Male	High risk	(DR3) - DQA1*05 - DQB1*02	DRB1*0404 - DQA1*03 - DQB1*0302	NA	NA	674	NA	NA	Batch 1	11Lr
11	AAb-	Male	High risk	(DR3) - DQA1*05 - DQB1*02	DRB1*0404 - DQA1*03 - DQB1*0302	NA	NA	852	NA	NA	Batch 1	11Cr
11	Progressor	Male	High risk	(DR3) - DQA1*05 - DQB1*02	DRB1*0401 - DQA1*03 - DQB1*0302	GADA, IAA	GADA, IAA, IA2A, ICA	95	520	1110	Batch 1	11P
11	Progressor	Male	High risk	(DR3) - DQA1*05 - DQB1*02	DRB1*0401 - DQA1*03 - DQB1*0302	GADA, IAA	GADA, IAA, IA2A, ICA	190	520	1110	Batch 1	11Sr
11	Progressor	Male	High risk	(DR3) - DQA1*05 - DQB1*02	DRB1*0401 - DQA1*03 - DQB1*0302	GADA, IAA	GADA, IAA, IA2A, ICA	304	520	1110	Batch 1	11K
11	Progressor	Male	High risk	(DR3) - DQA1*05 - DQB1*02	DRB1*0401 - DQA1*03 - DQB1*0302	GADA, IAA	GADA, IAA, IA2A, ICA	407	520	1110	Batch 1	11J1r
11	Progressor	Male	High risk	(DR3) - DQA1*05 - DQB1*02	DRB1*0401 - DQA1*03 - DQB1*0302	GADA, IAA	GADA, IAA, IA2A, ICA	520	520	1110	Batch 1	11H

11	Progressor	Male	High risk	(DR3) - DQA1*05 - DQB1*02	DRB1*0401 - DQA1*03 - DQB1*0302	GADA, IAA	GADA, IAA, IA2A, ICA	618	520	1110	Batch 1	11Nr
11	Progressor	Male	High risk	(DR3) - DQA1*05 - DQB1*02	DRB1*0401 - DQA1*03 - DQB1*0302	GADA, IAA	GADA, IAA, IA2A, ICA	717	520	1110	Batch 1	11F
11	Progressor	Male	High risk	(DR3) - DQA1*05 - DQB1*02	DRB1*0401 - DQA1*03 - DQB1*0302	GADA, IAA	GADA, IAA, IA2A, ICA	914	520	1110	Batch 1	11I
11	Progressor	Male	High risk	(DR3) - DQA1*05 - DQB1*02	DRB1*0401 - DQA1*03 - DQB1*0302	GADA, IAA	GADA, IAA, IA2A, ICA	1012	520	1110	Batch 1	11Mr
11	Progressor	Male	High risk	(DR3) - DQA1*05 - DQB1*02	DRB1*0401 - DQA1*03 - DQB1*0302	GADA, IAA	GADA, IAA, IA2A, ICA	1110	520	1110	Batch 1	11A
15	AAb-	Female	Moderately increased risk	DRB1*0401 - DQA1*03 - DQB1*0302	DRB1*0401 - DQA1*03 - DQB1*0302	NA	NA	198	NA	NA	Batch 1	15K
15	AAb-	Female	Moderately increased risk	DRB1*0401 - DQA1*03 - DQB1*0302	DRB1*0401 - DQA1*03 - DQB1*0302	NA	NA	281	NA	NA	Batch 1	15Lr
15	AAb-	Female	Moderately increased risk	DRB1*0401 - DQA1*03 - DQB1*0302	DRB1*0401 - DQA1*03 - DQB1*0302	NA	NA	377	NA	NA	Batch 1	15Sr
15	AAb-	Female	Moderately increased risk	DRB1*0401 - DQA1*03 - DQB1*0302	DRB1*0401 - DQA1*03 - DQB1*0302	NA	NA	481	NA	NA	Batch 1	15E
15	AAb-	Female	Moderately increased risk	DRB1*0401 - DQA1*03 - DQB1*0302	DRB1*0401 - DQA1*03 - DQB1*0302	NA	NA	565	NA	NA	Batch 1	15I
15	AAb-	Female	Moderately increased risk	DRB1*0401 - DQA1*03 - DQB1*0302	DRB1*0401 - DQA1*03 - DQB1*0302	NA	NA	645	NA	NA	Batch 1	15J
15	AAb-	Female	Moderately increased risk	DRB1*0401 - DQA1*03 - DQB1*0302	DRB1*0401 - DQA1*03 - DQB1*0302	NA	NA	757	NA	NA	Batch 1	15B
15	AAb-	Female	Moderately increased risk	DRB1*0401 - DQA1*03 - DQB1*0302	DRB1*0401 - DQA1*03 - DQB1*0302	NA	NA	894	NA	NA	Batch 1	15D
15	AAb-	Female	Moderately increased risk	DRB1*0401 - DQA1*03 - DQB1*0302	DRB1*0401 - DQA1*03 - DQB1*0302	NA	NA	1091	NA	NA	Batch 1	15P
15	Progressor	Female	Moderately increased risk	DRB1*0401 - DQA1*03 - DQB1*0302	(DR1/10) - DQB1*0501	IA2A, IAA, ICA	IA2A, IAA, ICA	96	379	1302	Batch 1	15G

15	Progressor	Female	Moderately increased risk	DRB1*0401 - DQA1*03 - DQB1*0302	(DR1/10) - DQB1*0501	IA2A, IAA, ICA	IA2A, IAA, ICA	181	379	1302	Batch 1	15F
15	Progressor	Female	Moderately increased risk	DRB1*0401 - DQA1*03 - DQB1*0302	(DR1/10) - DQB1*0501	IA2A, IAA, ICA	IA2A, IAA, ICA	281	379	1302	Batch 1	15Cr
15	Progressor	Female	Moderately increased risk	DRB1*0401 - DQA1*03 - DQB1*0302	(DR1/10) - DQB1*0501	IA2A, IAA, ICA	IA2A, IAA, ICA	379	379	1302	Batch 1	15Q
15	Progressor	Female	Moderately increased risk	DRB1*0401 - DQA1*03 - DQB1*0302	(DR1/10) - DQB1*0501	IA2A, IAA, ICA	IA2A, IAA, ICA	482	379	1302	Batch 1	15N
15	Progressor	Female	Moderately increased risk	DRB1*0401 - DQA1*03 - DQB1*0302	(DR1/10) - DQB1*0501	IA2A, IAA, ICA	IA2A, IAA, ICA	580	379	1302	Batch 1	15M
15	Progressor	Female	Moderately increased risk	DRB1*0401 - DQA1*03 - DQB1*0302	(DR1/10) - DQB1*0501	IA2A, IAA, ICA	IA2A, IAA, ICA	798	379	1302	Batch 1	15H
15	Progressor	Female	Moderately increased risk	DRB1*0401 - DQA1*03 - DQB1*0302	(DR1/10) - DQB1*0501	IA2A, IAA, ICA	IA2A, IAA, ICA	918	379	1302	Batch 1	15O
15	Progressor	Female	Moderately increased risk	DRB1*0401 - DQA1*03 - DQB1*0302	(DR1/10) - DQB1*0501	IA2A, IAA, ICA	IA2A, IAA, ICA	1009	379	1302	Batch 1	15R
15	Progressor	Female	Moderately increased risk	DRB1*0401 - DQA1*03 - DQB1*0302	(DR1/10) - DQB1*0501	IA2A, IAA, ICA	IA2A, IAA, ICA	1104	379	1302	Batch 1	15A
16	Progressor	Male	High risk	(DR3) - DQA1*05 - DQB1*02	DRB1*0401 - DQA1*03 - DQB1*0302	GADA, IAA	GADA, IAA, IA2A, ICA	110	467	876	Batch 1	16E
16	Progressor	Male	High risk	(DR3) - DQA1*05 - DQB1*02	DRB1*0401 - DQA1*03 - DQB1*0302	GADA, IAA	GADA, IAA, IA2A, ICA	193	467	876	Batch 1	16Q
16	Progressor	Male	High risk	(DR3) - DQA1*05 - DQB1*02	DRB1*0401 - DQA1*03 - DQB1*0302	GADA, IAA	GADA, IAA, IA2A, ICA	284	467	876	Batch 1	16H
16	Progressor	Male	High risk	(DR3) - DQA1*05 - DQB1*02	DRB1*0401 - DQA1*03 - DQB1*0302	GADA, IAA	GADA, IAA, IA2A, ICA	375	467	876	Batch 1	16M
16	Progressor	Male	High risk	(DR3) - DQA1*05 - DQB1*02	DRB1*0401 - DQA1*03 - DQB1*0302	GADA, IAA	GADA, IAA, IA2A, ICA	467	467	876	Batch 1	16Br
16	Progressor	Male	High risk	(DR3) - DQA1*05 - DQB1*02	DRB1*0401 - DQA1*03 - DQB1*0302	GADA, IAA	GADA, IAA, IA2A, ICA	584	467	876	Batch 1	16I

16	Progressor	Male	High risk	(DR3) - DQA1*05 - DQB1*02	DRB1*0401 - DQA1*03 - DQB1*0302	GADA, IAA	GADA, IAA, IA2A, ICA	674	467	876	Batch 1	16D
16	Progressor	Male	High risk	(DR3) - DQA1*05 - DQB1*02	DRB1*0401 - DQA1*03 - DQB1*0302	GADA, IAA	GADA, IAA, IA2A, ICA	719	467	876	Batch 1	16R
16	Progressor	Male	High risk	(DR3) - DQA1*05 - DQB1*02	DRB1*0401 - DQA1*03 - DQB1*0302	GADA, IAA	GADA, IAA, IA2A, ICA	807	467	876	Batch 1	16C
16	Progressor	Male	High risk	(DR3) - DQA1*05 - DQB1*02	DRB1*0401 - DQA1*03 - DQB1*0302	GADA, IAA	GADA, IAA, IA2A, ICA	878	467	876	Batch 1	16N
16	AAb-	Male	High risk	(DR3) - DQA1*05 - DQB1*02	DRB1*0401 - DQA1*03 - DQB1*0302	NA	NA	102	NA	NA	Batch 1	16G
16	AAb-	Male	High risk	(DR3) - DQA1*05 - DQB1*02	DRB1*0401 - DQA1*03 - DQB1*0302	NA	NA	187	NA	NA	Batch 1	16A
16	AAb-	Male	High risk	(DR3) - DQA1*05 - DQB1*02	DRB1*0401 - DQA1*03 - DQB1*0302	NA	NA	286	NA	NA	Batch 1	16Fr2
16	AAb-	Male	High risk	(DR3) - DQA1*05 - DQB1*02	DRB1*0401 - DQA1*03 - DQB1*0302	NA	NA	373	NA	NA	Batch 1	16S
16	AAb-	Male	High risk	(DR3) - DQA1*05 - DQB1*02	DRB1*0401 - DQA1*03 - DQB1*0302	NA	NA	460	NA	NA	Batch 1	16K
16	AAb-	Male	High risk	(DR3) - DQA1*05 - DQB1*02	DRB1*0401 - DQA1*03 - DQB1*0302	NA	NA	558	NA	NA	Batch 1	16L
16	AAb-	Male	High risk	(DR3) - DQA1*05 - DQB1*02	DRB1*0401 - DQA1*03 - DQB1*0302	NA	NA	653	NA	NA	Batch 1	16O
16	AAb-	Male	High risk	(DR3) - DQA1*05 - DQB1*02	DRB1*0401 - DQA1*03 - DQB1*0302	NA	NA	823	NA	NA	Batch 1	16P
16	AAb-	Male	High risk	(DR3) - DQA1*05 - DQB1*02	DRB1*0401 - DQA1*03 - DQB1*0302	NA	NA	979	NA	NA	Batch 1	16J
18	AAb-	Male	Moderately increased risk	DRB1*0401 - DQA1*03 - DQB1*0302	DRB1*0401 - DQA1*03 - DQB1*0302	NA	NA	108	NA	NA	Batch 1	18K
18	AAb-	Male	Moderately increased risk	DRB1*0401 - DQA1*03 - DQB1*0302	DRB1*0401 - DQA1*03 - DQB1*0302	NA	NA	189	NA	NA	Batch 1	18H

18	AAb-	Male	Moderately increased risk	DRB1*0401 - DQA1*03 - DQB1*0302	DRB1*0401 - DQA1*03 - DQB1*0302	NA	NA	286	NA	NA	Batch 1	18E
18	AAb-	Male	Moderately increased risk	DRB1*0401 - DQA1*03 - DQB1*0302	DRB1*0401 - DQA1*03 - DQB1*0302	NA	NA	374	NA	NA	Batch 1	18R
18	AAb-	Male	Moderately increased risk	DRB1*0401 - DQA1*03 - DQB1*0302	DRB1*0401 - DQA1*03 - DQB1*0302	NA	NA	467	NA	NA	Batch 1	18C
18	AAb-	Male	Moderately increased risk	DRB1*0401 - DQA1*03 - DQB1*0302	DRB1*0401 - DQA1*03 - DQB1*0302	NA	NA	570	NA	NA	Batch 1	18G
18	AAb-	Male	Moderately increased risk	DRB1*0401 - DQA1*03 - DQB1*0302	DRB1*0401 - DQA1*03 - DQB1*0302	NA	NA	651	NA	NA	Batch 1	18S
18	AAb-	Male	Moderately increased risk	DRB1*0401 - DQA1*03 - DQB1*0302	DRB1*0401 - DQA1*03 - DQB1*0302	NA	NA	739	NA	NA	Batch 1	18M
18	AAb-	Male	Moderately increased risk	DRB1*0401 - DQA1*03 - DQB1*0302	DRB1*0401 - DQA1*03 - DQB1*0302	NA	NA	922	NA	NA	Batch 1	18U
18	AAb-	Male	Moderately increased risk	DRB1*0401 - DQA1*03 - DQB1*0302	DRB1*0401 - DQA1*03 - DQB1*0302	NA	NA	1104	NA	NA	Batch 1	18T
18	Progressor	Male	Moderately increased risk	DRB1*0401 - DQA1*03 - DQB1*0302	(DR9) - DQA1*03 - DQB1*0303	ICA	GADA, IAA, ICA, IA2A	118	564	1000	Batch 1	18D
18	Progressor	Male	Moderately increased risk	DRB1*0401 - DQA1*03 - DQB1*0302	(DR9) - DQA1*03 - DQB1*0303	ICA	GADA, IAA, ICA, IA2A	201	564	1000	Batch 1	18Q
18	Progressor	Male	Moderately increased risk	DRB1*0401 - DQA1*03 - DQB1*0302	(DR9) - DQA1*03 - DQB1*0303	ICA	GADA, IAA, ICA, IA2A	280	564	1000	Batch 1	18N
18	Progressor	Male	Moderately increased risk	DRB1*0401 - DQA1*03 - DQB1*0302	(DR9) - DQA1*03 - DQB1*0303	ICA	GADA, IAA, ICA, IA2A	371	564	1000	Batch 1	18F
18	Progressor	Male	Moderately increased risk	DRB1*0401 - DQA1*03 - DQB1*0302	(DR9) - DQA1*03 - DQB1*0303	ICA	GADA, IAA, ICA, IA2A	469	564	1000	Batch 1	18O
18	Progressor	Male	Moderately increased risk	DRB1*0401 - DQA1*03 - DQB1*0302	(DR9) - DQA1*03 - DQB1*0303	ICA	GADA, IAA, ICA, IA2A	564	564	1000	Batch 1	18L
18	Progressor	Male	Moderately increased risk	DRB1*0401 - DQA1*03 - DQB1*0302	(DR9) - DQA1*03 - DQB1*0303	ICA	GADA, IAA, ICA, IA2A	663	564	1000	Batch 1	18P

18	Progressor	Male	Moderately increased risk	DRB1*0401 - DQA1*03 - DQB1*0302	(DR9) - DQA1*03 - DQB1*0303	ICA	GADA, IAA, ICA, IA2A	731	564	1000	Batch 1	18J
18	Progressor	Male	Moderately increased risk	DRB1*0401 - DQA1*03 - DQB1*0302	(DR9) - DQA1*03 - DQB1*0303	ICA	GADA, IAA, ICA, IA2A	840	564	1000	Batch 1	18B
18	Progressor	Male	Moderately increased risk	DRB1*0401 - DQA1*03 - DQB1*0302	(DR9) - DQA1*03 - DQB1*0303	ICA	GADA, IAA, ICA, IA2A	927	564	1000	Batch 1	18A
18	Progressor	Male	Moderately increased risk	DRB1*0401 - DQA1*03 - DQB1*0302	(DR9) - DQA1*03 - DQB1*0303	ICA	GADA, IAA, ICA, IA2A	1004	564	1000	Batch 1	18I
23	Progressor	Male	Moderately increased risk	DRB1*0401 - DQA1*03 - DQB1*0302	(DR8) - DQB1*04	GADA, IAA, ICA	GADA,IA2A, IAA, ICA	119	489	1372	Batch 1	23O
23	Progressor	Male	Moderately increased risk	DRB1*0401 - DQA1*03 - DQB1*0302	(DR8) - DQB1*04	GADA, IAA, ICA	GADA,IA2A, IAA, ICA	188	489	1372	Batch 1	23Fr
23	Progressor	Male	Moderately increased risk	DRB1*0401 - DQA1*03 - DQB1*0302	(DR8) - DQB1*04	GADA, IAA, ICA	GADA,IA2A, IAA, ICA	307	489	1372	Batch 1	23Gr
23	Progressor	Male	Moderately increased risk	DRB1*0401 - DQA1*03 - DQB1*0302	(DR8) - DQB1*04	GADA, IAA, ICA	GADA,IA2A, IAA, ICA	405	489	1372	Batch 1	23M
23	Progressor	Male	Moderately increased risk	DRB1*0401 - DQA1*03 - DQB1*0302	(DR8) - DQB1*04	GADA, IAA, ICA	GADA,IA2A, IAA, ICA	554	489	1372	Batch 1	23S
23	Progressor	Male	Moderately increased risk	DRB1*0401 - DQA1*03 - DQB1*0302	(DR8) - DQB1*04	GADA, IAA, ICA	GADA,IA2A, IAA, ICA	774	489	1372	Batch 1	23P
23	Progressor	Male	Moderately increased risk	DRB1*0401 - DQA1*03 - DQB1*0302	(DR8) - DQB1*04	GADA, IAA, ICA	GADA,IA2A, IAA, ICA	957	489	1372	Batch 1	23A
23	Progressor	Male	Moderately increased risk	DRB1*0401 - DQA1*03 - DQB1*0302	(DR8) - DQB1*04	GADA, IAA, ICA	GADA,IA2A, IAA, ICA	1153	489	1372	Batch 1	23I
23	Progressor	Male	Moderately increased risk	DRB1*0401 - DQA1*03 - DQB1*0302	(DR8) - DQB1*04	GADA, IAA, ICA	GADA,IA2A, IAA, ICA	1244	489	1372	Batch 1	23B
23	Progressor	Male	Moderately increased risk	DRB1*0401 - DQA1*03 - DQB1*0302	(DR8) - DQB1*04	GADA, IAA, ICA	GADA,IA2A, IAA, ICA	1364	489	1372	Batch 1	23C
23	AAb-	Male	Moderately increased risk	(DR7) - DQA1*0201 - DQB1*02	DRB1*0401 - DQA1*03 - DQB1*0302	NA	NA	89	NA	NA	Batch 1	23K

23	AAb-	Male	Moderately increased risk	(DR7) - DQA1*0201 - DQB1*02	DRB1*0401 - DQA1*03 - DQB1*0302	NA	NA	188	NA	NA	Batch 1	23J
23	AAb-	Male	Moderately increased risk	(DR7) - DQA1*0201 - DQB1*02	DRB1*0401 - DQA1*03 - DQB1*0302	NA	NA	369	NA	NA	Batch 1	23N
23	AAb-	Male	Moderately increased risk	(DR7) - DQA1*0201 - DQB1*02	DRB1*0401 - DQA1*03 - DQB1*0302	NA	NA	475	NA	NA	Batch 1	23E
23	AAb-	Male	Moderately increased risk	(DR7) - DQA1*0201 - DQB1*02	DRB1*0401 - DQA1*03 - DQB1*0302	NA	NA	565	NA	NA	Batch 1	23L
23	AAb-	Male	Moderately increased risk	(DR7) - DQA1*0201 - DQB1*02	DRB1*0401 - DQA1*03 - DQB1*0302	NA	NA	753	NA	NA	Batch 1	23D
23	AAb-	Male	Moderately increased risk	(DR7) - DQA1*0201 - DQB1*02	DRB1*0401 - DQA1*03 - DQB1*0302	NA	NA	935	NA	NA	Batch 1	23R
23	AAb-	Male	Moderately increased risk	(DR7) - DQA1*0201 - DQB1*02	DRB1*0401 - DQA1*03 - DQB1*0302	NA	NA	1287	NA	NA	Batch 1	23Q
23	AAb-	Male	Moderately increased risk	(DR7) - DQA1*0201 - DQB1*02	DRB1*0401 - DQA1*03 - DQB1*0302	NA	NA	1468	NA	NA	Batch 1	23H
34	Progressor	Female	High risk	(DR3) - DQA1*05 - DQB1*02	DRB1*0401 - DQA1*03 - DQB1*0302	GADA	GADA, IA2A, IAA , ICA	183	553	1203	Batch 1	34Q
34	Progressor	Female	High risk	(DR3) - DQA1*05 - DQB1*02	DRB1*0401 - DQA1*03 - DQB1*0302	GADA	GADA, IA2A, IAA , ICA	299	553	1203	Batch 1	34G
34	Progressor	Female	High risk	(DR3) - DQA1*05 - DQB1*02	DRB1*0401 - DQA1*03 - DQB1*0302	GADA	GADA, IA2A, IAA , ICA	453	553	1203	Batch 1	34L
34	Progressor	Female	High risk	(DR3) - DQA1*05 - DQB1*02	DRB1*0401 - DQA1*03 - DQB1*0302	GADA	GADA, IA2A, IAA , ICA	553	553	1203	Batch 1	34P
34	Progressor	Female	High risk	(DR3) - DQA1*05 - DQB1*02	DRB1*0401 - DQA1*03 - DQB1*0302	GADA	GADA, IA2A, IAA , ICA	645	553	1203	Batch 1	34C
34	Progressor	Female	High risk	(DR3) - DQA1*05 - DQB1*02	DRB1*0401 - DQA1*03 - DQB1*0302	GADA	GADA, IA2A, IAA , ICA	810	553	1203	Batch 1	34M
34	Progressor	Female	High risk	(DR3) - DQA1*05 - DQB1*02	DRB1*0401 - DQA1*03 - DQB1*0302	GADA	GADA, IA2A, IAA , ICA	1024	553	1203	Batch 1	34E

34	Progressor	Female	High risk	(DR3) - DQA1*05 - DQB1*02	DRB1*0401 - DQA1*03 - DQB1*0302	GADA	GADA, IA2A, IAA , ICA	1114	553	1203	Batch 1	34F
34	Progressor	Female	High risk	(DR3) - DQA1*05 - DQB1*02	DRB1*0401 - DQA1*03 - DQB1*0302	GADA	GADA, IA2A, IAA , ICA	1199	553	1203	Batch 1	34S
34	AAb-	Female	High risk	(DR3) - DQA1*05 - DQB1*02	DRB1*0404 - DQA1*03 - DQB1*0302	NA	NA	190	NA	NA	Batch 1	34R
34	AAb-	Female	High risk	(DR3) - DQA1*05 - DQB1*02	DRB1*0404 - DQA1*03 - DQB1*0302	NA	NA	274	NA	NA	Batch 1	34K
34	AAb-	Female	High risk	(DR3) - DQA1*05 - DQB1*02	DRB1*0404 - DQA1*03 - DQB1*0302	NA	NA	370	NA	NA	Batch 1	34D
34	AAb-	Female	High risk	(DR3) - DQA1*05 - DQB1*02	DRB1*0404 - DQA1*03 - DQB1*0302	NA	NA	453	NA	NA	Batch 1	34Tr
34	AAb-	Female	High risk	(DR3) - DQA1*05 - DQB1*02	DRB1*0404 - DQA1*03 - DQB1*0302	NA	NA	547	NA	NA	Batch 1	34B
34	AAb-	Female	High risk	(DR3) - DQA1*05 - DQB1*02	DRB1*0404 - DQA1*03 - DQB1*0302	NA	NA	644	NA	NA	Batch 1	34N
34	AAb-	Female	High risk	(DR3) - DQA1*05 - DQB1*02	DRB1*0404 - DQA1*03 - DQB1*0302	NA	NA	730	NA	NA	Batch 1	34I
34	AAb-	Female	High risk	(DR3) - DQA1*05 - DQB1*02	DRB1*0404 - DQA1*03 - DQB1*0302	NA	NA	817	NA	NA	Batch 1	34J
34	AAb-	Female	High risk	(DR3) - DQA1*05 - DQB1*02	DRB1*0404 - DQA1*03 - DQB1*0302	NA	NA	1009	NA	NA	Batch 1	34H
34	AAb-	Female	High risk	(DR3) - DQA1*05 - DQB1*02	DRB1*0404 - DQA1*03 - DQB1*0302	NA	NA	1192	NA	NA	Batch 1	34O
34	AAb-	Female	High risk	(DR3) - DQA1*05 - DQB1*02	DRB1*0404 - DQA1*03 - DQB1*0302	NA	NA	1379	NA	NA	Batch 1	34A
36	Progressor	Female	High risk	(DR3) - DQA1*05 - DQB1*02	DRB1*0401 - DQA1*03 - DQB1*0302	IAA	IAA, ICA, GADA, IA2A	190	402	1426	Batch 1	36V
36	Progressor	Female	High risk	(DR3) - DQA1*05 - DQB1*02	DRB1*0401 - DQA1*03 - DQB1*0302	IAA	IAA, ICA, GADA, IA2A	275	402	1426	Batch 1	36I

36	Progressor	Female	High risk	(DR3) - DQA1*05 - DQB1*02	DRB1*0401 - DQA1*03 - DQB1*0302	IAA	IAA, ICA, GADA, IA2A	402	402	1426	Batch 1	36B
36	Progressor	Female	High risk	(DR3) - DQA1*05 - DQB1*02	DRB1*0401 - DQA1*03 - DQB1*0302	IAA	IAA, ICA, GADA, IA2A	472	402	1426	Batch 1	36Gr
36	Progressor	Female	High risk	(DR3) - DQA1*05 - DQB1*02	DRB1*0401 - DQA1*03 - DQB1*0302	IAA	IAA, ICA, GADA, IA2A	556	402	1426	Batch 1	36S
36	Progressor	Female	High risk	(DR3) - DQA1*05 - DQB1*02	DRB1*0401 - DQA1*03 - DQB1*0302	IAA	IAA, ICA, GADA, IA2A	748	402	1426	Batch 1	36K
36	Progressor	Female	High risk	(DR3) - DQA1*05 - DQB1*02	DRB1*0401 - DQA1*03 - DQB1*0302	IAA	IAA, ICA, GADA, IA2A	934	402	1426	Batch 1	36J
36	Progressor	Female	High risk	(DR3) - DQA1*05 - DQB1*02	DRB1*0401 - DQA1*03 - DQB1*0302	IAA	IAA, ICA, GADA, IA2A	1228	402	1426	Batch 1	36N
36	Progressor	Female	High risk	(DR3) - DQA1*05 - DQB1*02	DRB1*0401 - DQA1*03 - DQB1*0302	IAA	IAA, ICA, GADA, IA2A	1284	402	1426	Batch 1	36E
36	Progressor	Female	High risk	(DR3) - DQA1*05 - DQB1*02	DRB1*0401 - DQA1*03 - DQB1*0302	IAA	IAA, ICA, GADA, IA2A	1389	402	1426	Batch 1	36U
36	Progressor	Female	High risk	(DR3) - DQA1*05 - DQB1*02	DRB1*0401 - DQA1*03 - DQB1*0302	IAA	IAA, ICA, GADA, IA2A	1429	402	1426	Batch 1	36A
36	AAb-	Female	High risk	(DR3) - DQA1*05 - DQB1*02	DRB1*0404 - DQA1*03 - DQB1*0302	NA	NA	182	NA	NA	Batch 1	36H
36	AAb-	Female	High risk	(DR3) - DQA1*05 - DQB1*02	DRB1*0404 - DQA1*03 - DQB1*0302	NA	NA	259	NA	NA	Batch 1	36R
36	AAb-	Female	High risk	(DR3) - DQA1*05 - DQB1*02	DRB1*0404 - DQA1*03 - DQB1*0302	NA	NA	372	NA	NA	Batch 1	36P
36	AAb-	Female	High risk	(DR3) - DQA1*05 - DQB1*02	DRB1*0404 - DQA1*03 - DQB1*0302	NA	NA	443	NA	NA	Batch 1	36T
36	AAb-	Female	High risk	(DR3) - DQA1*05 - DQB1*02	DRB1*0404 - DQA1*03 - DQB1*0302	NA	NA	538	NA	NA	Batch 1	36C
36	AAb-	Female	High risk	(DR3) - DQA1*05 - DQB1*02	DRB1*0404 - DQA1*03 - DQB1*0302	NA	NA	637	NA	NA	Batch 1	36D

36	AAb-	Female	High risk	(DR3) - DQA1*05 - DQB1*02	DRB1*0404 - DQA1*03 - DQB1*0302	NA	NA	724	NA	NA	Batch 1	36L
36	AAb-	Female	High risk	(DR3) - DQA1*05 - DQB1*02	DRB1*0404 - DQA1*03 - DQB1*0302	NA	NA	918	NA	NA	Batch 1	36M
36	AAb-	Female	High risk	(DR3) - DQA1*05 - DQB1*02	DRB1*0404 - DQA1*03 - DQB1*0302	NA	NA	1105	NA	NA	Batch 1	36F
36	AAb-	Female	High risk	(DR3) - DQA1*05 - DQB1*02	DRB1*0404 - DQA1*03 - DQB1*0302	NA	NA	1282	NA	NA	Batch 1	36O
36	AAb-	Female	High risk	(DR3) - DQA1*05 - DQB1*02	DRB1*0404 - DQA1*03 - DQB1*0302	NA	NA	1485	NA	NA	Batch 1	36Q
32	AAb-	Female	High risk	(DR3) - DQA1*05 - DQB1*02	DRB1*0404 - DQA1*03 - DQB1*0302	NA	NA	92	NA	NA	Batch 1	32C
32	AAb-	Female	High risk	(DR3) - DQA1*05 - DQB1*02	DRB1*0404 - DQA1*03 - DQB1*0302	NA	NA	182	NA	NA	Batch 1	32G
32	AAb-	Female	High risk	(DR3) - DQA1*05 - DQB1*02	DRB1*0404 - DQA1*03 - DQB1*0302	NA	NA	294	NA	NA	Batch 1	32F
32	AAb-	Female	High risk	(DR3) - DQA1*05 - DQB1*02	DRB1*0404 - DQA1*03 - DQB1*0302	NA	NA	379	NA	NA	Batch 1	32I
32	AAb-	Female	High risk	(DR3) - DQA1*05 - DQB1*02	DRB1*0404 - DQA1*03 - DQB1*0302	NA	NA	462	NA	NA	Batch 1	32Qr
32	AAb-	Female	High risk	(DR3) - DQA1*05 - DQB1*02	DRB1*0404 - DQA1*03 - DQB1*0302	NA	NA	575	NA	NA	Batch 1	32N
32	AAb-	Female	High risk	(DR3) - DQA1*05 - DQB1*02	DRB1*0404 - DQA1*03 - DQB1*0302	NA	NA	664	NA	NA	Batch 1	32Pr
32	AAb-	Female	High risk	(DR3) - DQA1*05 - DQB1*02	DRB1*0404 - DQA1*03 - DQB1*0302	NA	NA	739	NA	NA	Batch 1	32M
32	AAb-	Female	High risk	(DR3) - DQA1*05 - DQB1*02	DRB1*0404 - DQA1*03 - DQB1*0302	NA	NA	921	NA	NA	Batch 1	32B
32	Progressor	Female	High risk	(DR3) - DQA1*05 - DQB1*02	DRB1*0401 - DQA1*03 - DQB1*0302	IA2A, IAA, ICA	IA2A, IAA, ICA	90	736	882	Batch 1	32J

32	Progressor	Female	High risk	(DR3) - DQA1*05 - DQB1*02	DRB1*0401 - DQA1*03 - DQB1*0302	IA2A, IAA, ICA	IA2A, IAA, ICA	190	736	882	Batch 1	32D
32	Progressor	Female	High risk	(DR3) - DQA1*05 - DQB1*02	DRB1*0401 - DQA1*03 - DQB1*0302	IA2A, IAA, ICA	IA2A, IAA, ICA	384	736	882	Batch 1	32H
32	Progressor	Female	High risk	(DR3) - DQA1*05 - DQB1*02	DRB1*0401 - DQA1*03 - DQB1*0302	IA2A, IAA, ICA	IA2A, IAA, ICA	476	736	882	Batch 1	32E
32	Progressor	Female	High risk	(DR3) - DQA1*05 - DQB1*02	DRB1*0401 - DQA1*03 - DQB1*0302	IA2A, IAA, ICA	IA2A, IAA, ICA	736	736	882	Batch 1	32O
32	Progressor	Female	High risk	(DR3) - DQA1*05 - DQB1*02	DRB1*0401 - DQA1*03 - DQB1*0302	IA2A, IAA, ICA	IA2A, IAA, ICA	785	736	882	Batch 1	32K
32	Progressor	Female	High risk	(DR3) - DQA1*05 - DQB1*02	DRB1*0401 - DQA1*03 - DQB1*0302	IA2A, IAA, ICA	IA2A, IAA, ICA	864	736	882	Batch 1	32L
20	AAb-	Male	Moderately increased risk	DRB1*0401 - DQA1*03 - DQB1*0302	(DR8) - DQB1*04	NA	NA	123	NA	NA	Batch 2	20G
20	AAb-	Male	Moderately increased risk	DRB1*0401 - DQA1*03 - DQB1*0302	(DR8) - DQB1*04	NA	NA	291	NA	NA	Batch 2	20M
20	AAb-	Male	Moderately increased risk	DRB1*0401 - DQA1*03 - DQB1*0302	(DR8) - DQB1*04	NA	NA	375	NA	NA	Batch 2	20B
20	AAb-	Male	Moderately increased risk	DRB1*0401 - DQA1*03 - DQB1*0302	(DR8) - DQB1*04	NA	NA	488	NA	NA	Batch 2	20J
20	AAb-	Male	Moderately increased risk	DRB1*0401 - DQA1*03 - DQB1*0302	(DR8) - DQB1*04	NA	NA	572	NA	NA	Batch 2	20F
20	AAb-	Male	Moderately increased risk	DRB1*0401 - DQA1*03 - DQB1*0302	(DR8) - DQB1*04	NA	NA	684	NA	NA	Batch 2	20E
20	AAb-	Male	Moderately increased risk	DRB1*0401 - DQA1*03 - DQB1*0302	(DR8) - DQB1*04	NA	NA	796	NA	NA	Batch 2	20H
20	AAb-	Male	Moderately increased risk	DRB1*0401 - DQA1*03 - DQB1*0302	(DR8) - DQB1*04	NA	NA	1006	NA	NA	Batch 2	20A
20	AAb-	Male	Moderately increased risk	DRB1*0401 - DQA1*03 - DQB1*0302	(DR8) - DQB1*04	NA	NA	1218	NA	NA	Batch 2	20Q

20	Progressor	Male	Moderately increased risk	DRB1*0401 - DQA1*03 - DQB1*0302	(DR1/10) - DQB1*0501	IAA, ICA	IAA, ICA, IA2A, GADA	174	460	1184	Batch 2	20I
20	Progressor	Male	Moderately increased risk	DRB1*0401 - DQA1*03 - DQB1*0302	(DR1/10) - DQB1*0501	IAA, ICA	IAA, ICA, IA2A, GADA	289	460	1184	Batch 2	20R
20	Progressor	Male	Moderately increased risk	DRB1*0401 - DQA1*03 - DQB1*0302	(DR1/10) - DQB1*0501	IAA, ICA	IAA, ICA, IA2A, GADA	460	460	1184	Batch 2	20D
20	Progressor	Male	Moderately increased risk	DRB1*0401 - DQA1*03 - DQB1*0302	(DR1/10) - DQB1*0501	IAA, ICA	IAA, ICA, IA2A, GADA	558	460	1184	Batch 2	20N
20	Progressor	Male	Moderately increased risk	DRB1*0401 - DQA1*03 - DQB1*0302	(DR1/10) - DQB1*0501	IAA, ICA	IAA, ICA, IA2A, GADA	664	460	1184	Batch 2	20P
20	Progressor	Male	Moderately increased risk	DRB1*0401 - DQA1*03 - DQB1*0302	(DR1/10) - DQB1*0501	IAA, ICA	IAA, ICA, IA2A, GADA	778	460	1184	Batch 2	20O
20	Progressor	Male	Moderately increased risk	DRB1*0401 - DQA1*03 - DQB1*0302	(DR1/10) - DQB1*0501	IAA, ICA	IAA, ICA, IA2A, GADA	978	460	1184	Batch 2	20K
20	Progressor	Male	Moderately increased risk	DRB1*0401 - DQA1*03 - DQB1*0302	(DR1/10) - DQB1*0501	IAA, ICA	IAA, ICA, IA2A, GADA	1069	460	1184	Batch 2	20L
20	Progressor	Male	Moderately increased risk	DRB1*0401 - DQA1*03 - DQB1*0302	(DR1/10) - DQB1*0501	IAA, ICA	IAA, ICA, IA2A, GADA	1178	460	1184	Batch 2	20C
24	Progressor	Male	High risk	(DR3) - DQA1*05 - DQB1*02	DRB1*0404 - DQA1*03 - DQB1*0302	IAA	IAA, GADA, ICA	102	897	1308	Batch 2	24W
24	Progressor	Male	High risk	(DR3) - DQA1*05 - DQB1*02	DRB1*0404 - DQA1*03 - DQB1*0302	IAA	IAA, GADA, ICA	233	897	1308	Batch 2	24L_B14r
24	Progressor	Male	High risk	(DR3) - DQA1*05 - DQB1*02	DRB1*0404 - DQA1*03 - DQB1*0302	IAA	IAA, GADA, ICA	311	897	1308	Batch 2	24C
24	Progressor	Male	High risk	(DR3) - DQA1*05 - DQB1*02	DRB1*0404 - DQA1*03 - DQB1*0302	IAA	IAA, GADA, ICA	392	897	1308	Batch 2	24D
24	Progressor	Male	High risk	(DR3) - DQA1*05 - DQB1*02	DRB1*0404 - DQA1*03 - DQB1*0302	IAA	IAA, GADA, ICA	477	897	1308	Batch 2	24E_B14r
24	Progressor	Male	High risk	(DR3) - DQA1*05 - DQB1*02	DRB1*0404 - DQA1*03 - DQB1*0302	IAA	IAA, GADA, ICA	567	897	1308	Batch 2	24R

24	Progressor	Male	High risk	(DR3) - DQA1*05 - DQB1*02	DRB1*0404 - DQA1*03 - DQB1*0302	IAA	IAA, GADA, ICA	654	897	1308	Batch 2	24I
24	Progressor	Male	High risk	(DR3) - DQA1*05 - DQB1*02	DRB1*0404 - DQA1*03 - DQB1*0302	IAA	IAA, GADA, ICA	897	897	1308	Batch 2	24U_B14r
24	Progressor	Male	High risk	(DR3) - DQA1*05 - DQB1*02	DRB1*0404 - DQA1*03 - DQB1*0302	IAA	IAA, GADA, ICA	1183	897	1308	Batch 2	24S
24	Progressor	Male	High risk	(DR3) - DQA1*05 - DQB1*02	DRB1*0404 - DQA1*03 - DQB1*0302	IAA	IAA, GADA, ICA	1310	897	1308	Batch 2	24V
24	AAb-	Male	High risk	(DR3) - DQA1*05 - DQB1*02	DRB1*0404 - DQA1*03 - DQB1*0302	NA	NA	86	NA	NA	Batch 2	24M
24	AAb-	Male	High risk	(DR3) - DQA1*05 - DQB1*02	DRB1*0404 - DQA1*03 - DQB1*0302	NA	NA	190	NA	NA	Batch 2	24A
24	AAb-	Male	High risk	(DR3) - DQA1*05 - DQB1*02	DRB1*0404 - DQA1*03 - DQB1*0302	NA	NA	278	NA	NA	Batch 2	24J
24	AAb-	Male	High risk	(DR3) - DQA1*05 - DQB1*02	DRB1*0404 - DQA1*03 - DQB1*0302	NA	NA	358	NA	NA	Batch 2	24F
24	AAb-	Male	High risk	(DR3) - DQA1*05 - DQB1*02	DRB1*0404 - DQA1*03 - DQB1*0302	NA	NA	533	NA	NA	Batch 2	24B_B14r
24	AAb-	Male	High risk	(DR3) - DQA1*05 - DQB1*02	DRB1*0404 - DQA1*03 - DQB1*0302	NA	NA	625	NA	NA	Batch 2	24H
24	AAb-	Male	High risk	(DR3) - DQA1*05 - DQB1*02	DRB1*0404 - DQA1*03 - DQB1*0302	NA	NA	722	NA	NA	Batch 2	24P
24	AAb-	Male	High risk	(DR3) - DQA1*05 - DQB1*02	DRB1*0404 - DQA1*03 - DQB1*0302	NA	NA	912	NA	NA	Batch 2	24K
24	AAb-	Male	High risk	(DR3) - DQA1*05 - DQB1*02	DRB1*0404 - DQA1*03 - DQB1*0302	NA	NA	1087	NA	NA	Batch 2	24O
24	AAb-	Male	High risk	(DR3) - DQA1*05 - DQB1*02	DRB1*0404 - DQA1*03 - DQB1*0302	NA	NA	1277	NA	NA	Batch 2	24Q
24	AAb-	Male	High risk	(DR3) - DQA1*05 - DQB1*02	DRB1*0404 - DQA1*03 - DQB1*0302	NA	NA	1465	NA	NA	Batch 2	24G

119	Progressor	Female	Slightly increased risk	(DR3) - DQA1*05 - DQB1*02	(DR9) - DQA1*03 - DQB1*0303	GADA, IAA, ICA	GADA, IAA, ICA, IA2A	238	728	1398	Batch 2	119L
119	Progressor	Female	Slightly increased risk	(DR3) - DQA1*05 - DQB1*02	(DR9) - DQA1*03 - DQB1*0303	GADA, IAA, ICA	GADA, IAA, ICA, IA2A	337	728	1398	Batch 2	119M
119	Progressor	Female	Slightly increased risk	(DR3) - DQA1*05 - DQB1*02	(DR9) - DQA1*03 - DQB1*0303	GADA, IAA, ICA	GADA, IAA, ICA, IA2A	512	728	1398	Batch 2	119N
119	Progressor	Female	Slightly increased risk	(DR3) - DQA1*05 - DQB1*02	(DR9) - DQA1*03 - DQB1*0303	GADA, IAA, ICA	GADA, IAA, ICA, IA2A	728	728	1398	Batch 2	119G_B112r
119	Progressor	Female	Slightly increased risk	(DR3) - DQA1*05 - DQB1*02	(DR9) - DQA1*03 - DQB1*0303	GADA, IAA, ICA	GADA, IAA, ICA, IA2A	818	728	1398	Batch 2	119O
119	Progressor	Female	Slightly increased risk	(DR3) - DQA1*05 - DQB1*02	(DR9) - DQA1*03 - DQB1*0303	GADA, IAA, ICA	GADA, IAA, ICA, IA2A	911	728	1398	Batch 2	119P
119	Progressor	Female	Slightly increased risk	(DR3) - DQA1*05 - DQB1*02	(DR9) - DQA1*03 - DQB1*0303	GADA, IAA, ICA	GADA, IAA, ICA, IA2A	1113	728	1398	Batch 2	119D
119	Progressor	Female	Slightly increased risk	(DR3) - DQA1*05 - DQB1*02	(DR9) - DQA1*03 - DQB1*0303	GADA, IAA, ICA	GADA, IAA, ICA, IA2A	1303	728	1398	Batch 2	119Q
119	Progressor	Female	Slightly increased risk	(DR3) - DQA1*05 - DQB1*02	(DR9) - DQA1*03 - DQB1*0303	GADA, IAA, ICA	GADA, IAA, ICA, IA2A	1398	728	1398	Batch 2	119F
119	AAb-	Female	Slightly increased risk	(DR3) - DQA1*05 - DQB1*02	(DR9) - DQA1*03 - DQB1*0303	NA	NA	127	NA	NA	Batch 2	119J
119	AAb-	Female	Slightly increased risk	(DR3) - DQA1*05 - DQB1*02	(DR9) - DQA1*03 - DQB1*0303	NA	NA	219	NA	NA	Batch 2	119A
119	AAb-	Female	Slightly increased risk	(DR3) - DQA1*05 - DQB1*02	(DR9) - DQA1*03 - DQB1*0303	NA	NA	308	NA	NA	Batch 2	119H
119	AAb-	Female	Slightly increased risk	(DR3) - DQA1*05 - DQB1*02	(DR9) - DQA1*03 - DQB1*0303	NA	NA	398	NA	NA	Batch 2	119B_B112r
119	AAb-	Female	Slightly increased risk	(DR3) - DQA1*05 - DQB1*02	(DR9) - DQA1*03 - DQB1*0303	NA	NA	580	NA	NA	Batch 2	119E
119	AAb-	Female	Slightly increased risk	(DR3) - DQA1*05 - DQB1*02	(DR9) - DQA1*03 - DQB1*0303	NA	NA	763	NA	NA	Batch 2	119I

119	AAb-	Female	Slightly increased risk	(DR3) - DQA1*05 - DQB1*02	(DR9) - DQA1*03 - DQB1*0303	NA	NA	1106	NA	NA	Batch 2	119C
119	AAb-	Female	Slightly increased risk	(DR3) - DQA1*05 - DQB1*02	(DR9) - DQA1*03 - DQB1*0303	NA	NA	1416	NA	NA	Batch 2	119K
133	Progressor	Male	High risk	(DR3) - DQA1*05 - DQB1*02	DRB1*0401 - DQA1*03 - DQB1*0302	GADA, IAA, ICA	GADA, IAA, ICA, IA2A	96	557	920	Batch 2	133D
133	Progressor	Male	High risk	(DR3) - DQA1*05 - DQB1*02	DRB1*0401 - DQA1*03 - DQB1*0302	GADA, IAA, ICA	GADA, IAA, ICA, IA2A	187	557	920	Batch 2	133J
133	Progressor	Male	High risk	(DR3) - DQA1*05 - DQB1*02	DRB1*0401 - DQA1*03 - DQB1*0302	GADA, IAA, ICA	GADA, IAA, ICA, IA2A	389	557	920	Batch 2	133F
133	Progressor	Male	High risk	(DR3) - DQA1*05 - DQB1*02	DRB1*0401 - DQA1*03 - DQB1*0302	GADA, IAA, ICA	GADA, IAA, ICA, IA2A	557	557	920	Batch 2	133A
133	Progressor	Male	High risk	(DR3) - DQA1*05 - DQB1*02	DRB1*0401 - DQA1*03 - DQB1*0302	GADA, IAA, ICA	GADA, IAA, ICA, IA2A	650	557	920	Batch 2	133E
133	Progressor	Male	High risk	(DR3) - DQA1*05 - DQB1*02	DRB1*0401 - DQA1*03 - DQB1*0302	GADA, IAA, ICA	GADA, IAA, ICA, IA2A	732	557	920	Batch 2	133H
133	Progressor	Male	High risk	(DR3) - DQA1*05 - DQB1*02	DRB1*0401 - DQA1*03 - DQB1*0302	GADA, IAA, ICA	GADA, IAA, ICA, IA2A	823	557	920	Batch 2	133I
133	AAb-	Male	High risk	(DR3) - DQA1*05 - DQB1*02	DRB1*0404 - DQA1*03 - DQB1*0302	NA	NA	96	NA	NA	Batch 2	133M
133	AAb-	Male	High risk	(DR3) - DQA1*05 - DQB1*02	DRB1*0404 - DQA1*03 - DQB1*0302	NA	NA	187	NA	NA	Batch 2	133K
133	AAb-	Male	High risk	(DR3) - DQA1*05 - DQB1*02	DRB1*0404 - DQA1*03 - DQB1*0302	NA	NA	286	NA	NA	Batch 2	133C
133	AAb-	Male	High risk	(DR3) - DQA1*05 - DQB1*02	DRB1*0404 - DQA1*03 - DQB1*0302	NA	NA	385	NA	NA	Batch 2	133G
133	AAb-	Male	High risk	(DR3) - DQA1*05 - DQB1*02	DRB1*0404 - DQA1*03 - DQB1*0302	NA	NA	553	NA	NA	Batch 2	133B
133	AAb-	Male	High risk	(DR3) - DQA1*05 - DQB1*02	DRB1*0404 - DQA1*03 - DQB1*0302	NA	NA	735	NA	NA	Batch 2	133L

21	Progressor	Male	Slightly increased risk	(DR3) - DQA1*05 - DQB1*02	(DR13) - DQB1*0604	IAA, ICA	IAA, ICA	101	1091	1547	Batch 2	21Q
21	Progressor	Male	Slightly increased risk	(DR3) - DQA1*05 - DQB1*02	(DR13) - DQB1*0604	IAA, ICA	IAA, ICA	269	1091	1547	Batch 2	21A
21	Progressor	Male	Slightly increased risk	(DR3) - DQA1*05 - DQB1*02	(DR13) - DQB1*0604	IAA, ICA	IAA, ICA	373	1091	1547	Batch 2	21C
21	Progressor	Male	Slightly increased risk	(DR3) - DQA1*05 - DQB1*02	(DR13) - DQB1*0604	IAA, ICA	IAA, ICA	450	1091	1547	Batch 2	21E
21	Progressor	Male	Slightly increased risk	(DR3) - DQA1*05 - DQB1*02	(DR13) - DQB1*0604	IAA, ICA	IAA, ICA	572	1091	1547	Batch 2	21W
21	Progressor	Male	Slightly increased risk	(DR3) - DQA1*05 - DQB1*02	(DR13) - DQB1*0604	IAA, ICA	IAA, ICA	646	1091	1547	Batch 2	21M
21	Progressor	Male	Slightly increased risk	(DR3) - DQA1*05 - DQB1*02	(DR13) - DQB1*0604	IAA, ICA	IAA, ICA	738	1091	1547	Batch 2	21X
21	Progressor	Male	Slightly increased risk	(DR3) - DQA1*05 - DQB1*02	(DR13) - DQB1*0604	IAA, ICA	IAA, ICA	901	1091	1547	Batch 2	21T
21	Progressor	Male	Slightly increased risk	(DR3) - DQA1*05 - DQB1*02	(DR13) - DQB1*0604	IAA, ICA	IAA, ICA	1091	1091	1547	Batch 2	21K
21	Progressor	Male	Slightly increased risk	(DR3) - DQA1*05 - DQB1*02	(DR13) - DQB1*0604	IAA, ICA	IAA, ICA	1194	1091	1547	Batch 2	21R
21	Progressor	Male	Slightly increased risk	(DR3) - DQA1*05 - DQB1*02	(DR13) - DQB1*0604	IAA, ICA	IAA, ICA	1369	1091	1547	Batch 2	21J
21	Progressor	Male	Slightly increased risk	(DR3) - DQA1*05 - DQB1*02	(DR13) - DQB1*0604	IAA, ICA	IAA, ICA	1465	1091	1547	Batch 2	21H
21	AAb-	Male	Slightly increased risk	(DR3) - DQA1*05 - DQB1*02	(DR8) - DQB1*04	NA	NA	115	NA	NA	Batch 2	21L
21	AAb-	Male	Slightly increased risk	(DR3) - DQA1*05 - DQB1*02	(DR8) - DQB1*04	NA	NA	203	NA	NA	Batch 2	21I
21	AAb-	Male	Slightly increased risk	(DR3) - DQA1*05 - DQB1*02	(DR8) - DQB1*04	NA	NA	296	NA	NA	Batch 2	21D

21	AAb-	Male	Slightly increased risk	(DR3) - DQA1*05 - DQB1*02	(DR8) - DQB1*04	NA	NA	406	NA	NA	Batch 2	21B
21	AAb-	Male	Slightly increased risk	(DR3) - DQA1*05 - DQB1*02	(DR8) - DQB1*04	NA	NA	514	NA	NA	Batch 2	21F
21	AAb-	Male	Slightly increased risk	(DR3) - DQA1*05 - DQB1*02	(DR8) - DQB1*04	NA	NA	591	NA	NA	Batch 2	21O
21	AAb-	Male	Slightly increased risk	(DR3) - DQA1*05 - DQB1*02	(DR8) - DQB1*04	NA	NA	666	NA	NA	Batch 2	21G
21	AAb-	Male	Slightly increased risk	(DR3) - DQA1*05 - DQB1*02	(DR8) - DQB1*04	NA	NA	764	NA	NA	Batch 2	21U
21	AAb-	Male	Slightly increased risk	(DR3) - DQA1*05 - DQB1*02	(DR8) - DQB1*04	NA	NA	940	NA	NA	Batch 2	21P
21	AAb-	Male	Slightly increased risk	(DR3) - DQA1*05 - DQB1*02	(DR8) - DQB1*04	NA	NA	1151	NA	NA	Batch 2	21S
21	AAb-	Male	Slightly increased risk	(DR3) - DQA1*05 - DQB1*02	(DR8) - DQB1*04	NA	NA	1324	NA	NA	Batch 2	21V
21	AAb-	Male	Slightly increased risk	(DR3) - DQA1*05 - DQB1*02	(DR8) - DQB1*04	NA	NA	1486	NA	NA	Batch 2	21N
25	Progressor	Male	Moderately increased risk	DRB1*0401 - DQA1*03 - DQB1*0302	DRB1*0401 - DQA1*03 - DQB1*0302	IAA	IAA, IA2A, ICA	98	461	1470	Batch 2	25I
25	Progressor	Male	Moderately increased risk	DRB1*0401 - DQA1*03 - DQB1*0302	DRB1*0401 - DQA1*03 - DQB1*0302	IAA	IAA, IA2A, ICA	188	461	1470	Batch 2	25A
25	Progressor	Male	Moderately increased risk	DRB1*0401 - DQA1*03 - DQB1*0302	DRB1*0401 - DQA1*03 - DQB1*0302	IAA	IAA, IA2A, ICA	289	461	1470	Batch 2	25E
25	Progressor	Male	Moderately increased risk	DRB1*0401 - DQA1*03 - DQB1*0302	DRB1*0401 - DQA1*03 - DQB1*0302	IAA	IAA, IA2A, ICA	370	461	1470	Batch 2	25F
25	Progressor	Male	Moderately increased risk	DRB1*0401 - DQA1*03 - DQB1*0302	DRB1*0401 - DQA1*03 - DQB1*0302	IAA	IAA, IA2A, ICA	461	461	1470	Batch 2	25S
25	Progressor	Male	Moderately increased risk	DRB1*0401 - DQA1*03 - DQB1*0302	DRB1*0401 - DQA1*03 - DQB1*0302	IAA	IAA, IA2A, ICA	548	461	1470	Batch 2	25M

25	Progressor	Male	Moderately increased risk	DRB1*0401 - DQA1*03 - DQB1*0302	DRB1*0401 - DQA1*03 - DQB1*0302	IAA	IAA, IA2A, ICA	619	461	1470	Batch 2	25P
25	Progressor	Male	Moderately increased risk	DRB1*0401 - DQA1*03 - DQB1*0302	DRB1*0401 - DQA1*03 - DQB1*0302	IAA	IAA, IA2A, ICA	743	461	1470	Batch 2	25V
25	Progressor	Male	Moderately increased risk	DRB1*0401 - DQA1*03 - DQB1*0302	DRB1*0401 - DQA1*03 - DQB1*0302	IAA	IAA, IA2A, ICA	1100	461	1470	Batch 2	25B
25	Progressor	Male	Moderately increased risk	DRB1*0401 - DQA1*03 - DQB1*0302	DRB1*0401 - DQA1*03 - DQB1*0302	IAA	IAA, IA2A, ICA	1261	461	1470	Batch 2	25R
25	Progressor	Male	Moderately increased risk	DRB1*0401 - DQA1*03 - DQB1*0302	DRB1*0401 - DQA1*03 - DQB1*0302	IAA	IAA, IA2A, ICA	1476	461	1470	Batch 2	25L
25	AAb-	Male	Moderately increased risk	DRB1*0401 - DQA1*03 - DQB1*0302	(DR8) - DQB1*04	NA	NA	107	NA	NA	Batch 2	25Q
25	AAb-	Male	Moderately increased risk	DRB1*0401 - DQA1*03 - DQB1*0302	(DR8) - DQB1*04	NA	NA	183	NA	NA	Batch 2	25U
25	AAb-	Male	Moderately increased risk	DRB1*0401 - DQA1*03 - DQB1*0302	(DR8) - DQB1*04	NA	NA	289	NA	NA	Batch 2	25O
25	AAb-	Male	Moderately increased risk	DRB1*0401 - DQA1*03 - DQB1*0302	(DR8) - DQB1*04	NA	NA	375	NA	NA	Batch 2	25N
25	AAb-	Male	Moderately increased risk	DRB1*0401 - DQA1*03 - DQB1*0302	(DR8) - DQB1*04	NA	NA	477	NA	NA	Batch 2	25T
25	AAb-	Male	Moderately increased risk	DRB1*0401 - DQA1*03 - DQB1*0302	(DR8) - DQB1*04	NA	NA	557	NA	NA	Batch 2	25C
25	AAb-	Male	Moderately increased risk	DRB1*0401 - DQA1*03 - DQB1*0302	(DR8) - DQB1*04	NA	NA	655	NA	NA	Batch 2	25J
25	AAb-	Male	Moderately increased risk	DRB1*0401 - DQA1*03 - DQB1*0302	(DR8) - DQB1*04	NA	NA	766	NA	NA	Batch 2	25D
25	AAb-	Male	Moderately increased risk	DRB1*0401 - DQA1*03 - DQB1*0302	(DR8) - DQB1*04	NA	NA	969	NA	NA	Batch 2	25G
25	AAb-	Male	Moderately increased risk	DRB1*0401 - DQA1*03 - DQB1*0302	(DR8) - DQB1*04	NA	NA	1142	NA	NA	Batch 2	25H

25	AAb-	Male	Moderately increased risk	DRB1*0401 - DQA1*03 - DQB1*0302	(DR8) - DQB1*04	NA	NA	1321	NA	NA	Batch 2	25K
30	AAb-	Female	High risk	(DR3) - DQA1*05 - DQB1*02	DRB1*0401 - DQA1*03 - DQB1*0302	NA	NA	176	NA	NA	Batch 2	30O
30	AAb-	Female	High risk	(DR3) - DQA1*05 - DQB1*02	DRB1*0401 - DQA1*03 - DQB1*0302	NA	NA	274	NA	NA	Batch 2	30M
30	AAb-	Female	High risk	(DR3) - DQA1*05 - DQB1*02	DRB1*0401 - DQA1*03 - DQB1*0302	NA	NA	372	NA	NA	Batch 2	30T
30	AAb-	Female	High risk	(DR3) - DQA1*05 - DQB1*02	DRB1*0401 - DQA1*03 - DQB1*0302	NA	NA	456	NA	NA	Batch 2	30K
30	AAb-	Female	High risk	(DR3) - DQA1*05 - DQB1*02	DRB1*0401 - DQA1*03 - DQB1*0302	NA	NA	728	NA	NA	Batch 2	30F
30	AAb-	Female	High risk	(DR3) - DQA1*05 - DQB1*02	DRB1*0401 - DQA1*03 - DQB1*0302	NA	NA	1092	NA	NA	Batch 2	30Q
30	AAb-	Female	High risk	(DR3) - DQA1*05 - DQB1*02	DRB1*0401 - DQA1*03 - DQB1*0302	NA	NA	1288	NA	NA	Batch 2	30B
30	AAb-	Female	High risk	(DR3) - DQA1*05 - DQB1*02	DRB1*0401 - DQA1*03 - DQB1*0302	NA	NA	1456	NA	NA	Batch 2	30R
30	AAb-	Female	High risk	(DR3) - DQA1*05 - DQB1*02	DRB1*0401 - DQA1*03 - DQB1*0302	NA	NA	1652	NA	NA	Batch 2	30G
30	Progressor	Female	High risk	(DR3) - DQA1*05 - DQB1*02	DRB1*0401 - DQA1*03 - DQB1*0302	GADA, IAA	GADA, IAA, ICA	96	551	1648	Batch 2	30H
30	Progressor	Female	High risk	(DR3) - DQA1*05 - DQB1*02	DRB1*0401 - DQA1*03 - DQB1*0302	GADA, IAA	GADA, IAA, ICA	185	551	1648	Batch 2	30N
30	Progressor	Female	High risk	(DR3) - DQA1*05 - DQB1*02	DRB1*0401 - DQA1*03 - DQB1*0302	GADA, IAA	GADA, IAA, ICA	275	551	1648	Batch 2	30A
30	Progressor	Female	High risk	(DR3) - DQA1*05 - DQB1*02	DRB1*0401 - DQA1*03 - DQB1*0302	GADA, IAA	GADA, IAA, ICA	367	551	1648	Batch 2	30C
30	Progressor	Female	High risk	(DR3) - DQA1*05 - DQB1*02	DRB1*0401 - DQA1*03 - DQB1*0302	GADA, IAA	GADA, IAA, ICA	458	551	1648	Batch 2	30E_B16

30	Progressor	Female	High risk	(DR3) - DQA1*05 - DQB1*02	DRB1*0401 - DQA1*03 - DQB1*0302	GADA, IAA	GADA, IAA, ICA	642	551	1648	Batch 2	30S
30	Progressor	Female	High risk	(DR3) - DQA1*05 - DQB1*02	DRB1*0401 - DQA1*03 - DQB1*0302	GADA, IAA	GADA, IAA, ICA	764	551	1648	Batch 2	30J
30	Progressor	Female	High risk	(DR3) - DQA1*05 - DQB1*02	DRB1*0401 - DQA1*03 - DQB1*0302	GADA, IAA	GADA, IAA, ICA	928	551	1648	Batch 2	30I
30	Progressor	Female	High risk	(DR3) - DQA1*05 - DQB1*02	DRB1*0401 - DQA1*03 - DQB1*0302	GADA, IAA	GADA, IAA, ICA	1304	551	1648	Batch 2	30P
30	Progressor	Female	High risk	(DR3) - DQA1*05 - DQB1*02	DRB1*0401 - DQA1*03 - DQB1*0302	GADA, IAA	GADA, IAA, ICA	1460	551	1648	Batch 2	30L
30	Progressor	Female	High risk	(DR3) - DQA1*05 - DQB1*02	DRB1*0401 - DQA1*03 - DQB1*0302	GADA, IAA	GADA, IAA, ICA	1646	551	1648	Batch 2	30D
22	Progressor	Male	Moderately increased risk	DRB1*0401 - DQA1*03 - DQB1*0302	(DR13) - DQB1*0604	GADA, IAA	GADA, IAA, ICA	117	180	579	Batch 3	22B
22	Progressor	Male	Moderately increased risk	DRB1*0401 - DQA1*03 - DQB1*0302	(DR13) - DQB1*0604	GADA, IAA	GADA, IAA, ICA	180	180	579	Batch 3	22D
22	Progressor	Male	Moderately increased risk	DRB1*0401 - DQA1*03 - DQB1*0302	(DR13) - DQB1*0604	GADA, IAA	GADA, IAA, ICA	264	180	579	Batch 3	22H
22	Progressor	Male	Moderately increased risk	DRB1*0401 - DQA1*03 - DQB1*0302	(DR13) - DQB1*0604	GADA, IAA	GADA, IAA, ICA	367	180	579	Batch 3	22G
22	Progressor	Male	Moderately increased risk	DRB1*0401 - DQA1*03 - DQB1*0302	(DR13) - DQB1*0604	GADA, IAA	GADA, IAA, ICA	460	180	579	Batch 3	22J
22	Progressor	Male	Moderately increased risk	DRB1*0401 - DQA1*03 - DQB1*0302	(DR13) - DQB1*0604	GADA, IAA	GADA, IAA, ICA	549	180	579	Batch 3	22K
22	AAb-	Male	Moderately increased risk	DRB1*0401 - DQA1*03 - DQB1*0302	(DR13) - DQB1*0604	NA	NA	103	NA	NA	Batch 3	22F
22	AAb-	Male	Moderately increased risk	DRB1*0401 - DQA1*03 - DQB1*0302	(DR13) - DQB1*0604	NA	NA	203	NA	NA	Batch 3	22Ar
22	AAb-	Male	Moderately increased risk	DRB1*0401 - DQA1*03 - DQB1*0302	(DR13) - DQB1*0604	NA	NA	370	NA	NA	Batch 3	22C

22	AAb-	Male	Moderately increased risk	DRB1*0401 - DQA1*03 - DQB1*0302	(DR13) - DQB1*0604	NA	NA	463	NA	NA	Batch 3	22M
22	AAb-	Male	Moderately increased risk	DRB1*0401 - DQA1*03 - DQB1*0302	(DR13) - DQB1*0604	NA	NA	569	NA	NA	Batch 3	22L
22	AAb-	Male	Moderately increased risk	DRB1*0401 - DQA1*03 - DQB1*0302	(DR13) - DQB1*0604	NA	NA	643	NA	NA	Batch 3	22I
22	AAb-	Male	Moderately increased risk	DRB1*0401 - DQA1*03 - DQB1*0302	(DR13) - DQB1*0604	NA	NA	727	NA	NA	Batch 3	22E
10	Progressor	Male	Slightly increased risk	(DR3) - DQA1*05 - DQB1*02	(DR16) - DQB1*0502	IAA	IAA, GADA, ICA	88	193	433	Batch 3	10F
10	Progressor	Male	Slightly increased risk	(DR3) - DQA1*05 - DQB1*02	(DR16) - DQB1*0502	IAA	IAA, GADA, ICA	193	193	433	Batch 3	10Ir
10	Progressor	Male	Slightly increased risk	(DR3) - DQA1*05 - DQB1*02	(DR16) - DQB1*0502	IAA	IAA, GADA, ICA	305	193	433	Batch 3	10Gr
10	Progressor	Male	Slightly increased risk	(DR3) - DQA1*05 - DQB1*02	(DR16) - DQB1*0502	IAA	IAA, GADA, ICA	399	193	433	Batch 3	10A
10	Progressor	Male	Slightly increased risk	(DR3) - DQA1*05 - DQB1*02	(DR16) - DQB1*0502	IAA	IAA, GADA, ICA	420	193	433	Batch 3	10C
10	AAb-	Male	Slightly increased risk	(DR3) - DQA1*05 - DQB1*02	(DR8) - DQB1*04	NA	NA	99	NA	NA	Batch 3	10H
10	AAb-	Male	Slightly increased risk	(DR3) - DQA1*05 - DQB1*02	(DR8) - DQB1*04	NA	NA	255	NA	NA	Batch 3	10B
10	AAb-	Male	Slightly increased risk	(DR3) - DQA1*05 - DQB1*02	(DR8) - DQB1*04	NA	NA	409	NA	NA	Batch 3	10D
10	AAb-	Male	Slightly increased risk	(DR3) - DQA1*05 - DQB1*02	(DR8) - DQB1*04	NA	NA	525	NA	NA	Batch 3	10E
26	Progressor	Female	High risk	(DR3) - DQA1*05 - DQB1*02	DRB1*0401 - DQA1*03 - DQB1*0302	IAA	IAA, GADA, ICA	182	277	376	Batch 3	26E
26	Progressor	Female	High risk	(DR3) - DQA1*05 - DQB1*02	DRB1*0401 - DQA1*03 - DQB1*0302	IAA	IAA, GADA, ICA	277	277	376	Batch 3	26G

26	Progressor	Female	High risk	(DR3) - DQA1*05 - DQB1*02	DRB1*0401 - DQA1*03 - DQB1*0302	IAA	IAA, GADA, ICA	362	277	376	Batch 3	26F
26	AAb-	Female	High risk	(DR3) - DQA1*05 - DQB1*02	DRB1*0401 - DQA1*03 - DQB1*0302	NA	NA	190	NA	NA	Batch 3	26A
26	AAb-	Female	High risk	(DR3) - DQA1*05 - DQB1*02	DRB1*0401 - DQA1*03 - DQB1*0302	NA	NA	277	NA	NA	Batch 3	26B
26	AAb-	Female	High risk	(DR3) - DQA1*05 - DQB1*02	DRB1*0401 - DQA1*03 - DQB1*0302	NA	NA	372	NA	NA	Batch 3	26C
26	AAb-	Female	High risk	(DR3) - DQA1*05 - DQB1*02	DRB1*0401 - DQA1*03 - DQB1*0302	NA	NA	469	NA	NA	Batch 3	26D
28	Progressor	Female	High risk	(DR3) - DQA1*05 - DQB1*02	DRB1*0401 - DQA1*03 - DQB1*0302	IAA	IAA, GADA, ICA,IA2A	89	280	1133	Batch 3	28N
28	Progressor	Female	High risk	(DR3) - DQA1*05 - DQB1*02	DRB1*0401 - DQA1*03 - DQB1*0302	IAA	IAA, GADA, ICA,IA2A	193	280	1133	Batch 3	28F
28	Progressor	Female	High risk	(DR3) - DQA1*05 - DQB1*02	DRB1*0401 - DQA1*03 - DQB1*0302	IAA	IAA, GADA, ICA,IA2A	280	280	1133	Batch 3	28I
28	Progressor	Female	High risk	(DR3) - DQA1*05 - DQB1*02	DRB1*0401 - DQA1*03 - DQB1*0302	IAA	IAA, GADA, ICA,IA2A	383	280	1133	Batch 3	28M
28	Progressor	Female	High risk	(DR3) - DQA1*05 - DQB1*02	DRB1*0401 - DQA1*03 - DQB1*0302	IAA	IAA, GADA, ICA,IA2A	480	280	1133	Batch 3	28G
28	Progressor	Female	High risk	(DR3) - DQA1*05 - DQB1*02	DRB1*0401 - DQA1*03 - DQB1*0302	IAA	IAA, GADA, ICA,IA2A	567	280	1133	Batch 3	28B
28	Progressor	Female	High risk	(DR3) - DQA1*05 - DQB1*02	DRB1*0401 - DQA1*03 - DQB1*0302	IAA	IAA, GADA, ICA,IA2A	917	280	1133	Batch 3	28Q
28	Progressor	Female	High risk	(DR3) - DQA1*05 - DQB1*02	DRB1*0401 - DQA1*03 - DQB1*0302	IAA	IAA, GADA, ICA,IA2A	1126	280	1133	Batch 3	28D
28	AAb-	Female	High risk	(DR3) - DQA1*05 - DQB1*02	DRB1*0404 - DQA1*03 - DQB1*0302	NA	NA	92	NA	NA	Batch 3	28J
28	AAb-	Female	High risk	(DR3) - DQA1*05 - DQB1*02	DRB1*0404 - DQA1*03 - DQB1*0302	NA	NA	285	NA	NA	Batch 3	28E

28	AAb-	Female	High risk	(DR3) - DQA1*05 - DQB1*02	DRB1*0404 - DQA1*03 - DQB1*0302	NA	NA	410	NA	NA	Batch 3	28L
28	AAb-	Female	High risk	(DR3) - DQA1*05 - DQB1*02	DRB1*0404 - DQA1*03 - DQB1*0302	NA	NA	464	NA	NA	Batch 3	28C
28	AAb-	Female	High risk	(DR3) - DQA1*05 - DQB1*02	DRB1*0404 - DQA1*03 - DQB1*0302	NA	NA	561	NA	NA	Batch 3	28K
28	AAb-	Female	High risk	(DR3) - DQA1*05 - DQB1*02	DRB1*0404 - DQA1*03 - DQB1*0302	NA	NA	655	NA	NA	Batch 3	28P
28	AAb-	Female	High risk	(DR3) - DQA1*05 - DQB1*02	DRB1*0404 - DQA1*03 - DQB1*0302	NA	NA	754	NA	NA	Batch 3	28R
28	AAb-	Female	High risk	(DR3) - DQA1*05 - DQB1*02	DRB1*0404 - DQA1*03 - DQB1*0302	NA	NA	928	NA	NA	Batch 3	28Ar
28	AAb-	Female	High risk	(DR3) - DQA1*05 - DQB1*02	DRB1*0404 - DQA1*03 - DQB1*0302	NA	NA	1124	NA	NA	Batch 3	28H
28	AAb-	Female	High risk	(DR3) - DQA1*05 - DQB1*02	DRB1*0404 - DQA1*03 - DQB1*0302	NA	NA	1307	NA	NA	Batch 3	28O
35	Progressor	Male	Slightly increased risk	(DR3) - DQA1*05 - DQB1*02	(DR9) - DQA1*03 - DQB1*0303	GADA	GADA, IAA, ICA	145	267	825	Batch 3	35D
35	Progressor	Male	Slightly increased risk	(DR3) - DQA1*05 - DQB1*02	(DR9) - DQA1*03 - DQB1*0303	GADA	GADA, IAA, ICA	267	267	825	Batch 3	35H
35	Progressor	Male	Slightly increased risk	(DR3) - DQA1*05 - DQB1*02	(DR9) - DQA1*03 - DQB1*0303	GADA	GADA, IAA, ICA	365	267	825	Batch 3	35E
35	Progressor	Male	Slightly increased risk	(DR3) - DQA1*05 - DQB1*02	(DR9) - DQA1*03 - DQB1*0303	GADA	GADA, IAA, ICA	470	267	825	Batch 3	35K
35	Progressor	Male	Slightly increased risk	(DR3) - DQA1*05 - DQB1*02	(DR9) - DQA1*03 - DQB1*0303	GADA	GADA, IAA, ICA	579	267	825	Batch 3	35M
35	Progressor	Male	Slightly increased risk	(DR3) - DQA1*05 - DQB1*02	(DR9) - DQA1*03 - DQB1*0303	GADA	GADA, IAA, ICA	657	267	825	Batch 3	35C
35	Progressor	Male	Slightly increased risk	(DR3) - DQA1*05 - DQB1*02	(DR9) - DQA1*03 - DQB1*0303	GADA	GADA, IAA, ICA	746	267	825	Batch 3	35A

35	AAb-	Male	Slightly increased risk	(DR3) - DQA1*05 - DQB1*02	(DR1/10) - DQB1*0501	NA	NA	104	NA	NA	Batch 3	35L
35	AAb-	Male	Slightly increased risk	(DR3) - DQA1*05 - DQB1*02	(DR1/10) - DQB1*0501	NA	NA	181	NA	NA	Batch 3	35I
35	AAb-	Male	Slightly increased risk	(DR3) - DQA1*05 - DQB1*02	(DR1/10) - DQB1*0501	NA	NA	279	NA	NA	Batch 3	35F
35	AAb-	Male	Slightly increased risk	(DR3) - DQA1*05 - DQB1*02	(DR1/10) - DQB1*0501	NA	NA	350	NA	NA	Batch 3	35O
35	AAb-	Male	Slightly increased risk	(DR3) - DQA1*05 - DQB1*02	(DR1/10) - DQB1*0501	NA	NA	476	NA	NA	Batch 3	35G
35	AAb-	Male	Slightly increased risk	(DR3) - DQA1*05 - DQB1*02	(DR1/10) - DQB1*0501	NA	NA	620	NA	NA	Batch 3	35N
35	AAb-	Male	Slightly increased risk	(DR3) - DQA1*05 - DQB1*02	(DR1/10) - DQB1*0501	NA	NA	761	NA	NA	Batch 3	35J
35	AAb-	Male	Slightly increased risk	(DR3) - DQA1*05 - DQB1*02	(DR1/10) - DQB1*0501	NA	NA	894	NA	NA	Batch 3	35B
126	Progressor	Male	High risk	(DR3) - DQA1*05 - DQB1*02	DRB1*0401 - DQA1*03 - DQB1*0302	IAA, ICA	IAA, ICA	97	277	325	Batch 3	126A
126	Progressor	Male	High risk	(DR3) - DQA1*05 - DQB1*02	DRB1*0401 - DQA1*03 - DQB1*0302	IAA, ICA	IAA, ICA	186	277	325	Batch 3	126F
126	Progressor	Male	High risk	(DR3) - DQA1*05 - DQB1*02	DRB1*0401 - DQA1*03 - DQB1*0302	IAA, ICA	IAA, ICA	277	277	325	Batch 3	126C
126	AAb-	Male	High risk	(DR3) - DQA1*05 - DQB1*02	DRB1*0401 - DQA1*03 - DQB1*0302	NA	NA	94	NA	NA	Batch 3	126D
126	AAb-	Male	High risk	(DR3) - DQA1*05 - DQB1*02	DRB1*0401 - DQA1*03 - DQB1*0302	NA	NA	186	NA	NA	Batch 3	126G
126	AAb-	Male	High risk	(DR3) - DQA1*05 - DQB1*02	DRB1*0401 - DQA1*03 - DQB1*0302	NA	NA	277	NA	NA	Batch 3	126E
126	AAb-	Male	High risk	(DR3) - DQA1*05 - DQB1*02	DRB1*0401 - DQA1*03 - DQB1*0302	NA	NA	367	NA	NA	Batch 3	126B

129	Progressor	Female	Moderately increased risk	(DR3) - DQA1*05 - DQB1*02	(DR3) - DQA1*05 - DQB1*02	IAA,GADA, ICA	IAA,GADA, ICA	100	780	879	Batch 3	129H
129	Progressor	Female	Moderately increased risk	(DR3) - DQA1*05 - DQB1*02	(DR3) - DQA1*05 - DQB1*02	IAA,GADA, ICA	IAA,GADA, ICA	186	780	879	Batch 3	129C
129	Progressor	Female	Moderately increased risk	(DR3) - DQA1*05 - DQB1*02	(DR3) - DQA1*05 - DQB1*02	IAA,GADA, ICA	IAA,GADA, ICA	277	780	879	Batch 3	129I
129	Progressor	Female	Moderately increased risk	(DR3) - DQA1*05 - DQB1*02	(DR3) - DQA1*05 - DQB1*02	IAA,GADA, ICA	IAA,GADA, ICA	375	780	879	Batch 3	129A
129	Progressor	Female	Moderately increased risk	(DR3) - DQA1*05 - DQB1*02	(DR3) - DQA1*05 - DQB1*02	IAA,GADA, ICA	IAA,GADA, ICA	551	780	879	Batch 3	129E
129	Progressor	Female	Moderately increased risk	(DR3) - DQA1*05 - DQB1*02	(DR3) - DQA1*05 - DQB1*02	IAA,GADA, ICA	IAA,GADA, ICA	780	780	879	Batch 3	129J
129	AAb-	Female	Moderately increased risk	(DR3) - DQA1*05 - DQB1*02	(DR3) - DQA1*05 - DQB1*02	NA	NA	85	NA	NA	Batch 3	129G
129	AAb-	Female	Moderately increased risk	(DR3) - DQA1*05 - DQB1*02	(DR3) - DQA1*05 - DQB1*02	NA	NA	197	NA	NA	Batch 3	129K
129	AAb-	Female	Moderately increased risk	(DR3) - DQA1*05 - DQB1*02	(DR3) - DQA1*05 - DQB1*02	NA	NA	273	NA	NA	Batch 3	129D
129	AAb-	Female	Moderately increased risk	(DR3) - DQA1*05 - DQB1*02	(DR3) - DQA1*05 - DQB1*02	NA	NA	358	NA	NA	Batch 3	129F
129	AAb-	Female	Moderately increased risk	(DR3) - DQA1*05 - DQB1*02	(DR3) - DQA1*05 - DQB1*02	NA	NA	576	NA	NA	Batch 3	129B
129	AAb-	Female	Moderately increased risk	(DR3) - DQA1*05 - DQB1*02	(DR3) - DQA1*05 - DQB1*02	NA	NA	736	NA	NA	Batch 3	129L
136	Progressor	Female	Moderately increased risk	DRB1*0401 - DQA1*03 - DQB1*0302	(DR8) - DQB1*04	GADA, IA2A, ICA	GADA, IA2A, ICA, IAA	56	1095	1345	Batch 3	136M
136	Progressor	Female	Moderately increased risk	DRB1*0401 - DQA1*03 - DQB1*0302	(DR8) - DQB1*04	GADA, IA2A, ICA	GADA, IA2A, ICA, IAA	143	1095	1345	Batch 3	136E
136	Progressor	Female	Moderately increased risk	DRB1*0401 - DQA1*03 - DQB1*0302	(DR8) - DQB1*04	GADA, IA2A, ICA	GADA, IA2A, ICA, IAA	234	1095	1345	Batch 3	136L

136	Progressor	Female	Moderately increased risk	DRB1*0401 - DQA1*03 - DQB1*0302	(DR8) - DQB1*04	GADA, IA2A, ICA	GADA, IA2A, ICA, IAA	331	1095	1345	Batch 3	136H
136	Progressor	Female	Moderately increased risk	DRB1*0401 - DQA1*03 - DQB1*0302	(DR8) - DQB1*04	GADA, IA2A, ICA	GADA, IA2A, ICA, IAA	552	1095	1345	Batch 3	136J
136	Progressor	Female	Moderately increased risk	DRB1*0401 - DQA1*03 - DQB1*0302	(DR8) - DQB1*04	GADA, IA2A, ICA	GADA, IA2A, ICA, IAA	729	1095	1345	Batch 3	136C_is
136	Progressor	Female	Moderately increased risk	DRB1*0401 - DQA1*03 - DQB1*0302	(DR8) - DQB1*04	GADA, IA2A, ICA	GADA, IA2A, ICA, IAA	1095	1095	1345	Batch 3	136F
136	Progressor	Female	Moderately increased risk	DRB1*0401 - DQA1*03 - DQB1*0302	(DR8) - DQB1*04	GADA, IA2A, ICA	GADA, IA2A, ICA, IAA	1162	1095	1345	Batch 3	136B
136	Progressor	Female	Moderately increased risk	DRB1*0401 - DQA1*03 - DQB1*0302	(DR8) - DQB1*04	GADA, IA2A, ICA	GADA, IA2A, ICA, IAA	1240	1095	1345	Batch 3	136AcorN
136	Progressor	Female	Moderately increased risk	DRB1*0401 - DQA1*03 - DQB1*0302	(DR8) - DQB1*04	GADA, IA2A, ICA	GADA, IA2A, ICA, IAA	1344	1095	1345	Batch 3	136R
136	AAb-	Female	Moderately increased risk	DRB1*0401 - DQA1*03 - DQB1*0302	(DR1/10) - DQB1*0501	NA	NA	124	NA	NA	Batch 3	136KrExtend
136	AAb-	Female	Moderately increased risk	DRB1*0401 - DQA1*03 - DQB1*0302	(DR1/10) - DQB1*0501	NA	NA	248	NA	NA	Batch 3	136D
136	AAb-	Female	Moderately increased risk	DRB1*0401 - DQA1*03 - DQB1*0302	(DR1/10) - DQB1*0501	NA	NA	336	NA	NA	Batch 3	136P
136	AAb-	Female	Moderately increased risk	DRB1*0401 - DQA1*03 - DQB1*0302	(DR1/10) - DQB1*0501	NA	NA	517	NA	NA	Batch 3	136I
136	AAb-	Female	Moderately increased risk	DRB1*0401 - DQA1*03 - DQB1*0302	(DR1/10) - DQB1*0501	NA	NA	689	NA	NA	Batch 3	136G
136	AAb-	Female	Moderately increased risk	DRB1*0401 - DQA1*03 - DQB1*0302	(DR1/10) - DQB1*0501	NA	NA	889	NA	NA	Batch 3	136Q
136	AAb-	Female	Moderately increased risk	DRB1*0401 - DQA1*03 - DQB1*0302	(DR1/10) - DQB1*0501	NA	NA	1218	NA	NA	Batch 3	136N
136	AAb-	Female	Moderately increased risk	DRB1*0401 - DQA1*03 - DQB1*0302	(DR1/10) - DQB1*0501	NA	NA	1404	NA	NA	Batch 3	136O

14	Progressor	Male	Moderately increased risk	(DR7) - DQA1*0201 - DQB1*02	DRB1*0401 - DQA1*03 - DQB1*0302	GADA	GADA,IA2A, IAA, ICA	189	531	912	Batch 3	14B
14	Progressor	Male	Moderately increased risk	(DR7) - DQA1*0201 - DQB1*02	DRB1*0401 - DQA1*03 - DQB1*0302	GADA	GADA,IA2A, IAA, ICA	279	531	912	Batch 3	14J
14	Progressor	Male	Moderately increased risk	(DR7) - DQA1*0201 - DQB1*02	DRB1*0401 - DQA1*03 - DQB1*0302	GADA	GADA,IA2A, IAA, ICA	366	531	912	Batch 3	14E
14	Progressor	Male	Moderately increased risk	(DR7) - DQA1*0201 - DQB1*02	DRB1*0401 - DQA1*03 - DQB1*0302	GADA	GADA,IA2A, IAA, ICA	713	531	912	Batch 3	14A
14	Progressor	Male	Moderately increased risk	(DR7) - DQA1*0201 - DQB1*02	DRB1*0401 - DQA1*03 - DQB1*0302	GADA	GADA,IA2A, IAA, ICA	804	531	912	Batch 3	14D
14	Progressor	Male	Moderately increased risk	(DR7) - DQA1*0201 - DQB1*02	DRB1*0401 - DQA1*03 - DQB1*0302	GADA	GADA,IA2A, IAA, ICA	905	531	912	Batch 3	14K
14	AAb-	Male	Moderately increased risk	DRB1*0401 - DQA1*03 - DQB1*0302	(DR1/10) - DQB1*0501	NA	NA	268	NA	NA	Batch 3	14C
14	AAb-	Male	Moderately increased risk	DRB1*0401 - DQA1*03 - DQB1*0302	(DR1/10) - DQB1*0501	NA	NA	448	NA	NA	Batch 3	14I
14	AAb-	Male	Moderately increased risk	DRB1*0401 - DQA1*03 - DQB1*0302	(DR1/10) - DQB1*0501	NA	NA	631	NA	NA	Batch 3	14F
14	AAb-	Male	Moderately increased risk	DRB1*0401 - DQA1*03 - DQB1*0302	(DR1/10) - DQB1*0501	NA	NA	848	NA	NA	Batch 3	14G
14	AAb-	Male	Moderately increased risk	DRB1*0401 - DQA1*03 - DQB1*0302	(DR1/10) - DQB1*0501	NA	NA	995	NA	NA	Batch 3	14H
113	Progressor	Male	Moderately increased risk	(DR7) - DQA1*0201 - DQB1*02	DRB1*0401 - DQA1*03 - DQB1*0302	IAA,ICA	GADA,IA2A,IAA, ICA	117	521	923	Batch 3	113H
113	Progressor	Male	Moderately increased risk	(DR7) - DQA1*0201 - DQB1*02	DRB1*0401 - DQA1*03 - DQB1*0302	IAA,ICA	GADA,IA2A,IAA, ICA	236	521	923	Batch 3	113M
113	Progressor	Male	Moderately increased risk	(DR7) - DQA1*0201 - DQB1*02	DRB1*0401 - DQA1*03 - DQB1*0302	IAA,ICA	GADA,IA2A,IAA, ICA	340	521	923	Batch 3	113A
113	Progressor	Male	Moderately increased risk	(DR7) - DQA1*0201 - DQB1*02	DRB1*0401 - DQA1*03 - DQB1*0302	IAA,ICA	GADA,IA2A,IAA, ICA	617	521	923	Batch 3	113Gr

113	Progressor	Male	Moderately increased risk	(DR7) - DQA1*0201 - DQB1*02	DRB1*0401 - DQA1*03 - DQB1*0302	IAA,ICA	GADA,IA2A,IAA, ICA	703	521	923	Batch 3	113L
113	Progressor	Male	Moderately increased risk	(DR7) - DQA1*0201 - DQB1*02	DRB1*0401 - DQA1*03 - DQB1*0302	IAA,ICA	GADA,IA2A,IAA, ICA	801	521	923	Batch 3	113C
113	Progressor	Male	Moderately increased risk	(DR7) - DQA1*0201 - DQB1*02	DRB1*0401 - DQA1*03 - DQB1*0302	IAA,ICA	GADA,IA2A,IAA, ICA	897	521	923	Batch 3	113K
113	AAb-	Male	Moderately increased risk	DRB1*0401 - DQA1*03 - DQB1*0302	(DR8) - DQB1*04	NA	NA	121	NA	NA	Batch 3	113F
113	AAb-	Male	Moderately increased risk	DRB1*0401 - DQA1*03 - DQB1*0302	(DR8) - DQB1*04	NA	NA	218	NA	NA	Batch 3	113B
113	AAb-	Male	Moderately increased risk	DRB1*0401 - DQA1*03 - DQB1*0302	(DR8) - DQB1*04	NA	NA	307	NA	NA	Batch 3	113E
113	AAb-	Male	Moderately increased risk	DRB1*0401 - DQA1*03 - DQB1*0302	(DR8) - DQB1*04	NA	NA	398	NA	NA	Batch 3	113J
113	AAb-	Male	Moderately increased risk	DRB1*0401 - DQA1*03 - DQB1*0302	(DR8) - DQB1*04	NA	NA	583	NA	NA	Batch 3	113I
113	AAb-	Male	Moderately increased risk	DRB1*0401 - DQA1*03 - DQB1*0302	(DR8) - DQB1*04	NA	NA	757	NA	NA	Batch 3	113D
116	Progressor	Male	Moderately increased risk	DRB1*0401 - DQA1*03 - DQB1*0302	DRB1*0401 - DQA1*03 - DQB1*0302	IAA, GADA, ICA	GADA,IAA, ICA, IA2A	197	547	897	Batch 3	116D
116	Progressor	Male	Moderately increased risk	DRB1*0401 - DQA1*03 - DQB1*0302	DRB1*0401 - DQA1*03 - DQB1*0302	IAA, GADA, ICA	GADA,IAA, ICA, IA2A	281	547	897	Batch 3	116J
116	Progressor	Male	Moderately increased risk	DRB1*0401 - DQA1*03 - DQB1*0302	DRB1*0401 - DQA1*03 - DQB1*0302	IAA, GADA, ICA	GADA,IAA, ICA, IA2A	386	547	897	Batch 3	116M
116	Progressor	Male	Moderately increased risk	DRB1*0401 - DQA1*03 - DQB1*0302	DRB1*0401 - DQA1*03 - DQB1*0302	IAA, GADA, ICA	GADA,IAA, ICA, IA2A	547	547	897	Batch 3	116E
116	Progressor	Male	Moderately increased risk	DRB1*0401 - DQA1*03 - DQB1*0302	DRB1*0401 - DQA1*03 - DQB1*0302	IAA, GADA, ICA	GADA,IAA, ICA, IA2A	624	547	897	Batch 3	116C
116	Progressor	Male	Moderately increased risk	DRB1*0401 - DQA1*03 - DQB1*0302	DRB1*0401 - DQA1*03 - DQB1*0302	IAA, GADA, ICA	GADA,IAA, ICA, IA2A	757	547	897	Batch 3	116A

116	Progressor	Male	Moderately increased risk	DRB1*0401 - DQA1*03 - DQB1*0302	DRB1*0401 - DQA1*03 - DQB1*0302	IAA, GADA, ICA	GADA,IAA, ICA, IA2A	848	547	897	Batch 3	116B
116	AAb-	Male	Moderately increased risk	DRB1*0401 - DQA1*03 - DQB1*0302	DRB1*0401 - DQA1*03 - DQB1*0302	NA	NA	91	NA	NA	Batch 3	116H
116	AAb-	Male	Moderately increased risk	DRB1*0401 - DQA1*03 - DQB1*0302	DRB1*0401 - DQA1*03 - DQB1*0302	NA	NA	185	NA	NA	Batch 3	116L
116	AAb-	Male	Moderately increased risk	DRB1*0401 - DQA1*03 - DQB1*0302	DRB1*0401 - DQA1*03 - DQB1*0302	NA	NA	273	NA	NA	Batch 3	116G
116	AAb-	Male	Moderately increased risk	DRB1*0401 - DQA1*03 - DQB1*0302	DRB1*0401 - DQA1*03 - DQB1*0302	NA	NA	367	NA	NA	Batch 3	116I
116	AAb-	Male	Moderately increased risk	DRB1*0401 - DQA1*03 - DQB1*0302	DRB1*0401 - DQA1*03 - DQB1*0302	NA	NA	567	NA	NA	Batch 3	116K
116	AAb-	Male	Moderately increased risk	DRB1*0401 - DQA1*03 - DQB1*0302	DRB1*0401 - DQA1*03 - DQB1*0302	NA	NA	735	NA	NA	Batch 3	116F
12	Progressor	Male	High risk	(DR3) - DQA1*05 - DQB1*02	DRB1*0401 - DQA1*03 - DQB1*0302	IAA, ICA	IAA, ICA, IA2A	101	371	1488	Batch 3	12F
12	Progressor	Male	High risk	(DR3) - DQA1*05 - DQB1*02	DRB1*0401 - DQA1*03 - DQB1*0302	IAA, ICA	IAA, ICA, IA2A	226	371	1488	Batch 3	12A
12	Progressor	Male	High risk	(DR3) - DQA1*05 - DQB1*02	DRB1*0401 - DQA1*03 - DQB1*0302	IAA, ICA	IAA, ICA, IA2A	371	371	1488	Batch 3	12R
12	Progressor	Male	High risk	(DR3) - DQA1*05 - DQB1*02	DRB1*0401 - DQA1*03 - DQB1*0302	IAA, ICA	IAA, ICA, IA2A	471	371	1488	Batch 3	12J
12	Progressor	Male	High risk	(DR3) - DQA1*05 - DQB1*02	DRB1*0401 - DQA1*03 - DQB1*0302	IAA, ICA	IAA, ICA, IA2A	627	371	1488	Batch 3	12G
12	Progressor	Male	High risk	(DR3) - DQA1*05 - DQB1*02	DRB1*0401 - DQA1*03 - DQB1*0302	IAA, ICA	IAA, ICA, IA2A	735	371	1488	Batch 3	12C
12	Progressor	Male	High risk	(DR3) - DQA1*05 - DQB1*02	DRB1*0401 - DQA1*03 - DQB1*0302	IAA, ICA	IAA, ICA, IA2A	912	371	1488	Batch 3	12E
12	Progressor	Male	High risk	(DR3) - DQA1*05 - DQB1*02	DRB1*0401 - DQA1*03 - DQB1*0302	IAA, ICA	IAA, ICA, IA2A	1263	371	1488	Batch 3	12O

12	AAb-	Male	High risk	(DR3) - DQA1*05 - DQB1*02	DRB1*0401 - DQA1*03 - DQB1*0302	NA	NA	111	NA	NA	Batch 3	12K
12	AAb-	Male	High risk	(DR3) - DQA1*05 - DQB1*02	DRB1*0401 - DQA1*03 - DQB1*0302	NA	NA	182	NA	NA	Batch 3	12N
12	AAb-	Male	High risk	(DR3) - DQA1*05 - DQB1*02	DRB1*0401 - DQA1*03 - DQB1*0302	NA	NA	279	NA	NA	Batch 3	12L
12	AAb-	Male	High risk	(DR3) - DQA1*05 - DQB1*02	DRB1*0401 - DQA1*03 - DQB1*0302	NA	NA	456	NA	NA	Batch 3	12I
12	AAb-	Male	High risk	(DR3) - DQA1*05 - DQB1*02	DRB1*0401 - DQA1*03 - DQB1*0302	NA	NA	547	NA	NA	Batch 3	12H
12	AAb-	Male	High risk	(DR3) - DQA1*05 - DQB1*02	DRB1*0401 - DQA1*03 - DQB1*0302	NA	NA	645	NA	NA	Batch 3	12D
12	AAb-	Male	High risk	(DR3) - DQA1*05 - DQB1*02	DRB1*0401 - DQA1*03 - DQB1*0302	NA	NA	735	NA	NA	Batch 3	12B
12	AAb-	Male	High risk	(DR3) - DQA1*05 - DQB1*02	DRB1*0401 - DQA1*03 - DQB1*0302	NA	NA	925	NA	NA	Batch 3	12M
12	AAb-	Male	High risk	(DR3) - DQA1*05 - DQB1*02	DRB1*0401 - DQA1*03 - DQB1*0302	NA	NA	1099	NA	NA	Batch 3	12P
12	AAb-	Male	High risk	(DR3) - DQA1*05 - DQB1*02	DRB1*0401 - DQA1*03 - DQB1*0302	NA	NA	1338	NA	NA	Batch 3	12Q
17	Progressor	Female	Moderately increased risk	DRB1*0401 - DQA1*03 - DQB1*0302	DRB1*0401 - DQA1*03 - DQB1*0302	GADA, IAA	GADA, IAA, ICA, IA2A	464	654	1519	Batch 3	17J
17	Progressor	Female	Moderately increased risk	DRB1*0401 - DQA1*03 - DQB1*0302	DRB1*0401 - DQA1*03 - DQB1*0302	GADA, IAA	GADA, IAA, ICA, IA2A	654	654	1519	Batch 3	17N
17	Progressor	Female	Moderately increased risk	DRB1*0401 - DQA1*03 - DQB1*0302	DRB1*0401 - DQA1*03 - DQB1*0302	GADA, IAA	GADA, IAA, ICA, IA2A	898	654	1519	Batch 3	17M
17	Progressor	Female	Moderately increased risk	DRB1*0401 - DQA1*03 - DQB1*0302	DRB1*0401 - DQA1*03 - DQB1*0302	GADA, IAA	GADA, IAA, ICA, IA2A	1087	654	1519	Batch 3	17S
17	Progressor	Female	Moderately increased risk	DRB1*0401 - DQA1*03 - DQB1*0302	DRB1*0401 - DQA1*03 - DQB1*0302	GADA, IAA	GADA, IAA, ICA, IA2A	1267	654	1519	Batch 3	17A

17	Progressor	Female	Moderately increased risk	DRB1*0401 - DQA1*03 - DQB1*0302	DRB1*0401 - DQA1*03 - DQB1*0302	GADA, IAA	GADA, IAA, ICA, IA2A	1375	654	1519	Batch 3	17I
17	Progressor	Female	Moderately increased risk	DRB1*0401 - DQA1*03 - DQB1*0302	DRB1*0401 - DQA1*03 - DQB1*0302	GADA, IAA	GADA, IAA, ICA, IA2A	1466	654	1519	Batch 3	17O
17	Progressor	Female	Moderately increased risk	DRB1*0401 - DQA1*03 - DQB1*0302	DRB1*0401 - DQA1*03 - DQB1*0302	GADA, IAA	GADA, IAA, ICA, IA2A	1519	654	1519	Batch 3	17R
17	AAb-	Female	Moderately increased risk	DRB1*0401 - DQA1*03 - DQB1*0302	DRB1*0404 - DQA1*03 - DQB1*0302	NA	NA	101	NA	NA	Batch 3	17B
17	AAb-	Female	Moderately increased risk	DRB1*0401 - DQA1*03 - DQB1*0302	DRB1*0404 - DQA1*03 - DQB1*0302	NA	NA	192	NA	NA	Batch 3	17G
17	AAb-	Female	Moderately increased risk	DRB1*0401 - DQA1*03 - DQB1*0302	DRB1*0404 - DQA1*03 - DQB1*0302	NA	NA	368	NA	NA	Batch 3	17P
17	AAb-	Female	Moderately increased risk	DRB1*0401 - DQA1*03 - DQB1*0302	DRB1*0404 - DQA1*03 - DQB1*0302	NA	NA	444	NA	NA	Batch 3	17Q
17	AAb-	Female	Moderately increased risk	DRB1*0401 - DQA1*03 - DQB1*0302	DRB1*0404 - DQA1*03 - DQB1*0302	NA	NA	542	NA	NA	Batch 3	17L
17	AAb-	Female	Moderately increased risk	DRB1*0401 - DQA1*03 - DQB1*0302	DRB1*0404 - DQA1*03 - DQB1*0302	NA	NA	632	NA	NA	Batch 3	17D
17	AAb-	Female	Moderately increased risk	DRB1*0401 - DQA1*03 - DQB1*0302	DRB1*0404 - DQA1*03 - DQB1*0302	NA	NA	722	NA	NA	Batch 3	17C
17	AAb-	Female	Moderately increased risk	DRB1*0401 - DQA1*03 - DQB1*0302	DRB1*0404 - DQA1*03 - DQB1*0302	NA	NA	925	NA	NA	Batch 3	17T
17	AAb-	Female	Moderately increased risk	DRB1*0401 - DQA1*03 - DQB1*0302	DRB1*0404 - DQA1*03 - DQB1*0302	NA	NA	1092	NA	NA	Batch 3	17F
17	AAb-	Female	Moderately increased risk	DRB1*0401 - DQA1*03 - DQB1*0302	DRB1*0404 - DQA1*03 - DQB1*0302	NA	NA	1289	NA	NA	Batch 3	17K
17	AAb-	Female	Moderately increased risk	DRB1*0401 - DQA1*03 - DQB1*0302	DRB1*0404 - DQA1*03 - DQB1*0302	NA	NA	1464	NA	NA	Batch 3	17H
17	AAb-	Female	Moderately increased risk	DRB1*0401 - DQA1*03 - DQB1*0302	DRB1*0404 - DQA1*03 - DQB1*0302	NA	NA	1646	NA	NA	Batch 3	17E

29	Progressor	Male	High risk	(DR3) - DQA1*05 - DQB1*02	DRB1*0401 - DQA1*03 - DQB1*0302	GADA, IAA, ICA	GADA, IAA, ICA, IA2A	122	529	1518	Batch 3	29K
29	Progressor	Male	High risk	(DR3) - DQA1*05 - DQB1*02	DRB1*0401 - DQA1*03 - DQB1*0302	GADA, IAA, ICA	GADA, IAA, ICA, IA2A	211	529	1518	Batch 3	29R
29	Progressor	Male	High risk	(DR3) - DQA1*05 - DQB1*02	DRB1*0401 - DQA1*03 - DQB1*0302	GADA, IAA, ICA	GADA, IAA, ICA, IA2A	529	529	1518	Batch 3	29D
29	Progressor	Male	High risk	(DR3) - DQA1*05 - DQB1*02	DRB1*0401 - DQA1*03 - DQB1*0302	GADA, IAA, ICA	GADA, IAA, ICA, IA2A	633	529	1518	Batch 3	29P
29	Progressor	Male	High risk	(DR3) - DQA1*05 - DQB1*02	DRB1*0401 - DQA1*03 - DQB1*0302	GADA, IAA, ICA	GADA, IAA, ICA, IA2A	724	529	1518	Batch 3	29Q
29	Progressor	Male	High risk	(DR3) - DQA1*05 - DQB1*02	DRB1*0401 - DQA1*03 - DQB1*0302	GADA, IAA, ICA	GADA, IAA, ICA, IA2A	1143	529	1518	Batch 3	29M
29	Progressor	Male	High risk	(DR3) - DQA1*05 - DQB1*02	DRB1*0401 - DQA1*03 - DQB1*0302	GADA, IAA, ICA	GADA, IAA, ICA, IA2A	1340	529	1518	Batch 3	29B
29	Progressor	Male	High risk	(DR3) - DQA1*05 - DQB1*02	DRB1*0401 - DQA1*03 - DQB1*0302	GADA, IAA, ICA	GADA, IAA, ICA, IA2A	1446	529	1518	Batch 3	29U
29	Progressor	Male	High risk	(DR3) - DQA1*05 - DQB1*02	DRB1*0401 - DQA1*03 - DQB1*0302	GADA, IAA, ICA	GADA, IAA, ICA, IA2A	1520	529	1518	Batch 3	29G
29	AAb-	Male	High risk	(DR3) - DQA1*05 - DQB1*02	DRB1*0401 - DQA1*03 - DQB1*0302	NA	NA	98	NA	NA	Batch 3	29L
29	AAb-	Male	High risk	(DR3) - DQA1*05 - DQB1*02	DRB1*0401 - DQA1*03 - DQB1*0302	NA	NA	181	NA	NA	Batch 3	29E
29	AAb-	Male	High risk	(DR3) - DQA1*05 - DQB1*02	DRB1*0401 - DQA1*03 - DQB1*0302	NA	NA	273	NA	NA	Batch 3	29F
29	AAb-	Male	High risk	(DR3) - DQA1*05 - DQB1*02	DRB1*0401 - DQA1*03 - DQB1*0302	NA	NA	363	NA	NA	Batch 3	29J
29	AAb-	Male	High risk	(DR3) - DQA1*05 - DQB1*02	DRB1*0401 - DQA1*03 - DQB1*0302	NA	NA	464	NA	NA	Batch 3	29T
29	AAb-	Male	High risk	(DR3) - DQA1*05 - DQB1*02	DRB1*0401 - DQA1*03 - DQB1*0302	NA	NA	562	NA	NA	Batch 3	29I

29	AAb-	Male	High risk	(DR3) - DQA1*05 - DQB1*02	DRB1*0401 - DQA1*03 - DQB1*0302	NA	NA	652	NA	NA	Batch 3	29H
29	AAb-	Male	High risk	(DR3) - DQA1*05 - DQB1*02	DRB1*0401 - DQA1*03 - DQB1*0302	NA	NA	750	NA	NA	Batch 3	29N
29	AAb-	Male	High risk	(DR3) - DQA1*05 - DQB1*02	DRB1*0401 - DQA1*03 - DQB1*0302	NA	NA	939	NA	NA	Batch 3	29C
29	AAb-	Male	High risk	(DR3) - DQA1*05 - DQB1*02	DRB1*0401 - DQA1*03 - DQB1*0302	NA	NA	1106	NA	NA	Batch 3	29S
29	AAb-	Male	High risk	(DR3) - DQA1*05 - DQB1*02	DRB1*0401 - DQA1*03 - DQB1*0302	NA	NA	1309	NA	NA	Batch 3	29V
29	AAb-	Male	High risk	(DR3) - DQA1*05 - DQB1*02	DRB1*0401 - DQA1*03 - DQB1*0302	NA	NA	1456	NA	NA	Batch 3	29O
29	AAb-	Male	High risk	(DR3) - DQA1*05 - DQB1*02	DRB1*0401 - DQA1*03 - DQB1*0302	NA	NA	1666	NA	NA	Batch 3	29A
31	Progressor	Female	High risk	(DR3) - DQA1*05 - DQB1*02	DRB1*0401 - DQA1*03 - DQB1*0302	GADA	GADA, IA2A, ICA	565	972	1811	Batch 3	31I
31	Progressor	Female	High risk	(DR3) - DQA1*05 - DQB1*02	DRB1*0401 - DQA1*03 - DQB1*0302	GADA	GADA, IA2A, ICA	972	972	1811	Batch 3	31F
31	Progressor	Female	High risk	(DR3) - DQA1*05 - DQB1*02	DRB1*0401 - DQA1*03 - DQB1*0302	GADA	GADA, IA2A, ICA	1677	972	1811	Batch 3	31E
31	AAb-	Female	High risk	(DR3) - DQA1*05 - DQB1*02	DRB1*0404 - DQA1*03 - DQB1*0302	NA	NA	108	NA	NA	Batch 3	31M
31	AAb-	Female	High risk	(DR3) - DQA1*05 - DQB1*02	DRB1*0404 - DQA1*03 - DQB1*0302	NA	NA	173	NA	NA	Batch 3	31L
31	AAb-	Female	High risk	(DR3) - DQA1*05 - DQB1*02	DRB1*0404 - DQA1*03 - DQB1*0302	NA	NA	278	NA	NA	Batch 3	31Q
31	AAb-	Female	High risk	(DR3) - DQA1*05 - DQB1*02	DRB1*0404 - DQA1*03 - DQB1*0302	NA	NA	355	NA	NA	Batch 3	31C
31	AAb-	Female	High risk	(DR3) - DQA1*05 - DQB1*02	DRB1*0404 - DQA1*03 - DQB1*0302	NA	NA	460	NA	NA	Batch 3	31J

31	AAb-	Female	High risk	(DR3) - DQA1*05 - DQB1*02	DRB1*0404 - DQA1*03 - DQB1*0302	NA	NA	564	NA	NA	Batch 3	31K
31	AAb-	Female	High risk	(DR3) - DQA1*05 - DQB1*02	DRB1*0404 - DQA1*03 - DQB1*0302	NA	NA	633	NA	NA	Batch 3	31H
31	AAb-	Female	High risk	(DR3) - DQA1*05 - DQB1*02	DRB1*0404 - DQA1*03 - DQB1*0302	NA	NA	719	NA	NA	Batch 3	31D
31	AAb-	Female	High risk	(DR3) - DQA1*05 - DQB1*02	DRB1*0404 - DQA1*03 - DQB1*0302	NA	NA	880	NA	NA	Batch 3	31B
31	AAb-	Female	High risk	(DR3) - DQA1*05 - DQB1*02	DRB1*0404 - DQA1*03 - DQB1*0302	NA	NA	1083	NA	NA	Batch 3	31A
31	AAb-	Female	High risk	(DR3) - DQA1*05 - DQB1*02	DRB1*0404 - DQA1*03 - DQB1*0302	NA	NA	1264	NA	NA	Batch 3	31N
31	AAb-	Female	High risk	(DR3) - DQA1*05 - DQB1*02	DRB1*0404 - DQA1*03 - DQB1*0302	NA	NA	1448	NA	NA	Batch 3	31G
31	AAb-	Female	High risk	(DR3) - DQA1*05 - DQB1*02	DRB1*0404 - DQA1*03 - DQB1*0302	NA	NA	1615	NA	NA	Batch 3	31O
31	AAb-	Female	High risk	(DR3) - DQA1*05 - DQB1*02	DRB1*0404 - DQA1*03 - DQB1*0302	NA	NA	1840	NA	NA	Batch 3	31P
33	Progressor	Male	Moderately increased risk	DRB1*0401 - DQA1*03 - DQB1*0302	(DR1/10) - DQB1*0501	IAA	IAA, GADA, IA2A, ICA	110	188	1785	Batch 3	33C
33	Progressor	Male	Moderately increased risk	DRB1*0401 - DQA1*03 - DQB1*0302	(DR1/10) - DQB1*0501	IAA	IAA, GADA, IA2A, ICA	188	188	1785	Batch 3	33A
33	Progressor	Male	Moderately increased risk	DRB1*0401 - DQA1*03 - DQB1*0302	(DR1/10) - DQB1*0501	IAA	IAA, GADA, IA2A, ICA	272	188	1785	Batch 3	33L
33	Progressor	Male	Moderately increased risk	DRB1*0401 - DQA1*03 - DQB1*0302	(DR1/10) - DQB1*0501	IAA	IAA, GADA, IA2A, ICA	371	188	1785	Batch 3	33F
33	Progressor	Male	Moderately increased risk	DRB1*0401 - DQA1*03 - DQB1*0302	(DR1/10) - DQB1*0501	IAA	IAA, GADA, IA2A, ICA	466	188	1785	Batch 3	33S
33	Progressor	Male	Moderately increased risk	DRB1*0401 - DQA1*03 - DQB1*0302	(DR1/10) - DQB1*0501	IAA	IAA, GADA, IA2A, ICA	557	188	1785	Batch 3	33E

33	Progressor	Male	Moderately increased risk	DRB1*0401 - DQA1*03 - DQB1*0302	(DR1/10) - DQB1*0501	IAA	IAA, GADA, IA2A, ICA	875	188	1785	Batch 3	33K
33	Progressor	Male	Moderately increased risk	DRB1*0401 - DQA1*03 - DQB1*0302	(DR1/10) - DQB1*0501	IAA	IAA, GADA, IA2A, ICA	1487	188	1785	Batch 3	33Q
33	Progressor	Male	Moderately increased risk	DRB1*0401 - DQA1*03 - DQB1*0302	(DR1/10) - DQB1*0501	IAA	IAA, GADA, IA2A, ICA	1571	188	1785	Batch 3	33O
33	Progressor	Male	Moderately increased risk	DRB1*0401 - DQA1*03 - DQB1*0302	(DR1/10) - DQB1*0501	IAA	IAA, GADA, IA2A, ICA	1685	188	1785	Batch 3	33R
33	Progressor	Male	Moderately increased risk	DRB1*0401 - DQA1*03 - DQB1*0302	(DR1/10) - DQB1*0501	IAA	IAA, GADA, IA2A, ICA	1774	188	1785	Batch 3	33T
33	AAb-	Male	Moderately increased risk	DRB1*0401 - DQA1*03 - DQB1*0302	(DR1/10) - DQB1*0501	NA	NA	138	NA	NA	Batch 3	33P
33	AAb-	Male	Moderately increased risk	DRB1*0401 - DQA1*03 - DQB1*0302	(DR1/10) - DQB1*0501	NA	NA	249	NA	NA	Batch 3	33V
33	AAb-	Male	Moderately increased risk	DRB1*0401 - DQA1*03 - DQB1*0302	(DR1/10) - DQB1*0501	NA	NA	367	NA	NA	Batch 3	33D
33	AAb-	Male	Moderately increased risk	DRB1*0401 - DQA1*03 - DQB1*0302	(DR1/10) - DQB1*0501	NA	NA	473	NA	NA	Batch 3	33N
33	AAb-	Male	Moderately increased risk	DRB1*0401 - DQA1*03 - DQB1*0302	(DR1/10) - DQB1*0501	NA	NA	565	NA	NA	Batch 3	33J
33	AAb-	Male	Moderately increased risk	DRB1*0401 - DQA1*03 - DQB1*0302	(DR1/10) - DQB1*0501	NA	NA	642	NA	NA	Batch 3	33H
33	AAb-	Male	Moderately increased risk	DRB1*0401 - DQA1*03 - DQB1*0302	(DR1/10) - DQB1*0501	NA	NA	752	NA	NA	Batch 3	33I
33	AAb-	Male	Moderately increased risk	DRB1*0401 - DQA1*03 - DQB1*0302	(DR1/10) - DQB1*0501	NA	NA	920	NA	NA	Batch 3	33M
33	AAb-	Male	Moderately increased risk	DRB1*0401 - DQA1*03 - DQB1*0302	(DR1/10) - DQB1*0501	NA	NA	1109	NA	NA	Batch 3	33U
33	AAb-	Male	Moderately increased risk	DRB1*0401 - DQA1*03 - DQB1*0302	(DR1/10) - DQB1*0501	NA	NA	1452	NA	NA	Batch 3	33G

33	AAb-	Male	Moderately increased risk	DRB1*0401 - DQA1*03 - DQB1*0302	(DR1/10) - DQB1*0501	NA	NA	1662	NA	NA	Batch 3	33B

Supplementary Table S5. LonGP results of the 22 peptides representing the 11 proteins selected for the targeted proteomics analysis.

Gene name	Peptide sequence	Model name	Converge flag	age	sero	t1d	gender	group	pair	batch	id	age* batch	age*id	noise
IGJ	FVYHLSDLCK	model 0 ~ age+batch+id+ age*batch+age*id	2	11.8%	0	0	0	0	0	14.7%	5.3%	1.2%	43.8%	23.2%
IGJ	SSEDPNEDIVER	model 0 ~ age+id+age*id	2	18.2%	0	0	0	0	0	0	8.1%	0	45.6%	28.0%
APOA4	LGEVNTYAGDLQK	model 0 ~ age+batch+id+ age*batch+age*id	2	52.4%	0	0	0	0	0	4.7%	9.1%	0.5%	2.8%	30.6%
APOA4	ISASAEELR	model 0 ~ age+batch+id+ age*batch+age*id	2	47.0%	0	0	0	0	0	6.4%	6.1%	0.3%	1.8%	38.3%
APOA4	LAPLAEDVR	model 0 ~ age+batch+id+ age*batch+age*id	2	49.0%	0	0	0	0	0	4.2%	8.4%	0.1%	1.7%	36.6%
GC	HLSLLTTLSNR	model 0 ~ age+batch+id+ age*batch+age*id	2	0.2%	0	0	0	0	0	91.3%	3.7%	1.7%	0.5%	2.7%
GC	VLEPTLK	model 0 ~ age+t1d+id+age*id	2	4.3%	0	1.0%	0	0	0	0	43.7%	0	44.1%	6.9%
LPA	GTYSTTVTGR	model 0 ~ age+id+age*id	2	2.1%	0	0	0	0	0	0	89.6%	0	8.0%	0.4%
LPA	NPDAVAAPYCYTR	model 0 ~ age+id+age*id	2	2.0%	0	0	0	0	0	0	89.4%	0	8.1%	0.4%
IGFBP2	LEGEACGVYTPR	model 0 ~ age+batch+id+ age*batch+age*id	2	33.9%	0	0	0	0	0	23.6%	6.6%	0.5%	8.4%	27.0%
IGFBP2	LIQGAPTIR	model 0 ~ age+pair+id+age*id	2	56.7%	0	0	0	0	2.2%	0	1.3%	0	10.4%	29.4%
TF	DGAGDVAFVK	model 0 ~ age+id+age*id	2	4.7%	0	0	0	0	0	0	49.3%	0	45.0%	1.0%
TF	EDPQTFYYAVAVVK	model 0 ~ age+sero+batch+ id+age*batch+age*id	2	3.2%	0.4%	0	0	0	0	22.6%	30.7%	2.1%	0.7%	40.2%
F12	NGPLSCGQR	model 0 ~ age+batch+id+ age*batch+age*id	2	0.6%	0	0	0	0	0	9.7%	69.7%	0.3%	1.3%	18.5%
F12	CLEVEGHR	model 0 ~ age+id+age*id	2	0.9%	0	0	0	0	0	0	74.4%	0	1.4%	23.4%

CRISP3	YEDLYSNCK	model 0 ~ age+batch+id+ age*batch+age*id	2	0.6%	0	0	0	0	0	16.9%	24.0%	0.3%	3.3%	54.8%
APOC1	EFGNTLEDK	model 0 ~ age+sero+batch +id+age*batch+age*id	2	3.9%	4.6%	0	0	0	0	23.1%	19.4%	1.0%	3.8%	44.3%
APOC1	EWFSETFQK	model 0 ~ age+sero+pair+ batch+id+age*batch+age*id	2	2.5%	4.9%	0	0	0	13.3%	18.3%	9.5%	0.6%	12.7%	38.2%
IGHM	YAATSQVLLPSK	model 0 ~ age+batch+id+ age*batch+age*id	2	10.1%	0	0	0	0	0	9.5%	9.1%	0.5%	50.5%	20.2%
IGHM	QVGSGVTTDQVQAEAK	model 0 ~ age+batch+id+ age*batch+age*id	2	11.0%	0	0	0	0	0	14.8%	5.9%	0.7%	49.0%	18.7%
CD5L	IWLDNVR	model 0 ~ age+batch+id+ age*batch+age*id	2	11.1%	0	0	0	0	0	5.1%	3.9%	0.1%	63.1%	16.5%
CD5L	EATLQDCPSGPWGK	model 0 ~ age+id+age*id	2	14.4%	0	0	0	0	0	0	11.3%	0	54.3%	20.0%

Supplementary Table S6. Peptides and transitions selected for the targeted proteomics analysis.

Gene name	Protein Accession	Peptide	Precursor	Fragments	Fragment type	Isotope
A1BG	P04217	SGLSTGWTQLSK	632.8302	1007.516, 920.4836, 819.4359	y9, y8, y7	light
A1BG	P04217	ATWSGAVLAGR	544.796	916.4999, 730.4206, 643.3886	y9, y8, y7	light
IGHM	P01871	YAATSQVLLPSK	639.3586	1043.61, 444.2817, 331.1976	y10, y4, y3	light
IGHM	P01871	YAATSQVLLPSK	643.3657	1051.624, 452.2959, 339.2118	y10, y4, y3	heavy
IGHM	P01871	QVGSGVTTDQVQAEAK	809.4076	1090.537, 546.2882, 418.2296, 218.1499	y10, y5, y4, y2	light
IGHM	P01871	QVGSGVTTDQVQAEAK	813.4147	1098.552, 554.3024, 426.2438, 226.1641	y10, y5, y4, y2	heavy
CRISP3	P54108	YEDLYSNC[+57.021464]K	596.253	899.3927, 784.3658, 671.2817, 508.2184	y7, y6, y5, y4	light
CRISP3	P54108	YEDLYSNC[+57.021464]K	600.2601	907.4069, 792.38, 679.2959, 516.2326	y7, y6, y5, y4	heavy
CD5L	O43866	EATLQDC[+57.021464]PSGPWGK	773.3537	1003.43, 888.4032, 728.3726	y9, y8, y7	light
CD5L	O43866	EATLQDC[+57.021464]PSGPWGK	777.3608	1011.444, 896.4174, 736.3868	y9, y8, y7	heavy
CD5L	O43866	IWLDNVR	458.256	802.4206, 616.3413, 503.2572	y6, y5, y4	light
CD5L	O43866	IWLDNVR	463.2601	812.4289, 626.3496, 513.2655	y6, y5, y4	heavy
APOA4	P06727	LGEVNTYAGDLQK	704.3594	1009.495, 794.4043, 631.341	y9, y7, y6	light
APOA4	P06727	LGEVNTYAGDLQK	708.3665	1017.509, 802.4185, 639.3552	y9, y7, y6	heavy
APOA4	P06727	ISASAEELR	488.2589	862.4265, 775.3945, 704.3573	y8, y7, y6	light
APOA4	P06727	ISASAEELR	493.263	872.4348, 785.4027, 714.3656	y8, y7, y6	heavy
APOA4	P06727	LAPLAEDVR	492.2796	799.4308, 589.294, 274.1874	y7, y5, y2	light
APOA4	P06727	LAPLAEDVR	497.2838	809.4391, 599.3023, 284.1956	y7, y5, y2	heavy
IGJ	P01591	SSEDPNEDIVER	695.3101	1086.506, 971.4793, 486.2433	y9, y8, y8	light
IGJ	P01591	SSEDPNEDIVER	700.3142	1096.514, 981.4875, 491.2474	y9, y8, y8	heavy
IGJ	P01591	FVYHLSDLC[+57.021464]K	427.8814	735.3706, 622.2865, 436.7184	y6, y5, y7	light
IGJ	P01591	FVYHLSDLC[+57.021464]K	430.5528	743.3848, 630.3007, 440.7255	y6, y5, y7	heavy
APOC1	P02654	EFGNTLEDK	526.7484	776.3785, 605.3141, 391.1823, 262.1397	y7, y5, y3, y2	light
APOC1	P02654	EFGNTLEDK	530.7555	784.3927, 613.3283, 399.1965, 270.1539	y7, y5, y3, y2	heavy
APOC1	P02654	EWFSETFQK	601.2798	886.4305, 739.3621, 523.2875	y7, y6, y4	light
APOC1	P02654	EWFSETFQK	605.2869	894.4447, 747.3763, 531.3017	y7, y6, y4	heavy
IGFBP2	P18065	LEGEAC[+57.021464]GVYTPR	676.3192	1109.504, 923.4404, 852.4032, 692.3726	y10, y8, y7, y6	light
IGFBP2	P18065	LEGEAC[+57.021464]GVYTPR	681.3233	1119.513, 933.4486, 862.4115, 702.3809	y10, y8, y7, y6	heavy
IGFBP2	P18065	LIQGAPTIR	484.798	742.4206, 614.362, 486.3035	y7, y6, y4	light
IGFBP2	P18065	LIQGAPTIR	489.8021	752.4289, 624.3703, 496.3117	y7, y6, y4	heavy
LPA	P08519	GTYSTTVTGR	521.7618	884.4472, 721.3839, 634.3519, 533.3042	y8, y7, y6, y5	light
LPA	P08519	GTYSTTVTGR	526.766	894.4555, 731.3922, 644.3601, 543.3125	y8, y7, y6, y5	heavy
LPA	P08519	NPDAVAAPYC[+57.021464]YTR	749.3432	1171.556, 1100.519, 1001.451, 930.4138	y10, y9, y8, y7	light
LPA	P08519	NPDAVAAPYC[+57.021464]YTR	754.3473	1181.565, 1110.528, 1011.459, 940.4221	y10, y9, y8, y7	heavy

TF	P02787	EDPQTFYYAVAVVK	815.4116	1160.635, 1059.587, 912.5189	y10, y9, y8	light
TF	P02787	EDPQTFYYAVAVVK	819.4187	1168.649, 1067.602, 920.5331	y10, y9, y8	heavy
TF	P02787	DGAGDVAFVK	489.7482	735.4036, 563.3552, 464.2867	y7, y5, y4	light
TF	P02787	DGAGDVAFVK	493.7553	743.4178, 571.3694, 472.3009	y7, y5, y4	heavy
F12	P00748	C[+57.021464]LEVEGHR	333.8274	597.3103, 498.2419, 369.1993	y5, y4, y3	light
F12	P00748	C[+57.021464]LEVEGHR	337.1635	607.3186, 508.2502, 379.2076	y5, y4, y3	heavy
F12	P00748	NGPLSC[+57.021464]GQR	494.7351	817.3985, 720.3457, 607.2617, 520.2296	y7, y6, y5, y4	light
F12	P00748	NGPLSC[+57.021464]GQR	499.7392	827.4068, 730.354, 617.2699, 530.2379	y7, y6, y5, y4	heavy
GC	P02774	HLSLLTTLSNR	627.8619	1117.658, 691.3733, 251.1503	y10, y6, b2	light
GC	P02774	HLSLLTTLSNR	632.866	1127.666, 701.3816, 251.1503	y10, y6, b2	heavy
GC	P02774	VLEPTLK	400.2498	700.424, 587.3399, 458.2973	y6, y5, y4	light
GC	P02774	VLEPTLK	404.2569	708.4382, 595.3541, 466.3115	y6, y5, y4	heavy
MSRT1	MSRT1	LGGNETQVR	492.2608	870.4303, 813.4089, 513.3019	y8, y7, y4	heavy
MSRT1	MSRT1	AEFAEVSK	444.7313	688.3756, 541.3072, 201.087	y6, y5, b2	heavy
MSRT1	MSRT1	SGFSSVSVSR	511.7607	731.3922, 644.3601, 458.2597	y7, y6, y4	heavy
MSRT1	MSRT1	ADEGISFR	452.7236	718.3758, 589.3332, 419.2277	y6, y5, y3	heavy
MSRT1	MSRT1	DISLSDYK	474.7418	720.3654, 520.2493, 318.1903	y6, y4, y2	heavy
MSRT1	MSRT1	DQGGELLSLR	549.2949	854.497, 611.4114, 244.0928	y8, y5, b2	heavy
MSRT1	MSRT1	GLFIIDDK	464.7651	758.4174, 498.265, 270.1539	y6, y4, y2	heavy
MSRT1	MSRT1	YWGVASFLQK	603.8235	857.4971, 701.4072, 350.1499	y8, y6, b2	heavy
MSRT1	MSRT1	TDELFQIEGLKEELAYLR	726.3836	859.9712, 217.0819, 346.1245	y14, b2, b3	heavy
MSRT1	MSRT1	AVQQPDGLAVLGIFLK	838.9949	1250.76, 171.1128, 299.1714	y12, b2, b3	heavy
MSRT1	MSRT1	LGEYGFQNAL	559.7831	210.1562, 139.1191, 300.1667	y2, y1, y5	heavy

Supplementary Table S7. The relative standard deviation (%RSD) of the raw peak intensities of a pooled serum sampled that served as a quality control (QC). Batch 1 included 24 QC analyses, Batch 2 included 8 QC analyses and Batch 3 included 15 QC analyses.

			Batch 1			Batch 2			Batch3			Median across assays		
Gene name	Protein Accession	Peptide Sequence	LIGHT %RSD	HEAVY %RSD	L/H %RSD	LIGHT %RSD	HEAVY %RSD	L/H %RSD	LIGHT %RSD	HEAVY %RSD	L/H %RSD	LIGHT %RSD	HEAVY %RSD	L/H %RSD
A1BG	P04217	SGLSTGWTQLSK	1.8 (0.9-3.0)	5.9 (2.9-7.5)	5.4 (2.6-9.4)	4.2 (3.9-4.5)	11 (6.0-16)	3.5 (2.5-4.4)	3.6 (1.5-9.7)	9.9 (9.5-15)	6.6 (1.7-16)	3.6	9.9	5.5
A1BG	P04217	ATWSGAVLAGR	3.6 (1.6-5.0)	6.7 (5.4-11)	7 (3.1-12)	7.2 (2.9-11)	11 (7.2-16)	2.6 (2.4-2.8)	4.9 (3.7-5.2)	4.0 (2.8-10)	3.9 (2.5-14)	4.9	6.7	3.9
IGHM	P01871	YAATSQVLLPSK	1.6 (0.9-4.0)	5.9 (1.7-9.7)	6.5 (2.7-11)	4.1 (1.6-6.6)	3.5 (2.7-4.3)	1.7 (1.2-2.3)	6.9 (6.6-21)	9.6 (9.3-17)	13 (3.6-16)	4.1	5.9	6.5
IGHM	P01871	QVGSGVTTDQVQAEAK	6.2 (1.9-10.)	9.4 (6.4-10)	11.0 (5.1-17)	5.6 (2.9-8.4)	7.1 (3.8-10)	5.3 (4.7-5.9)	3.9 (2.4-8.3)	12 (10.-14)	12 (6.1-13)	5.6	9.4	11
CRISP3	P54108	YEDLYSNCK	5.6 (2.6-8.6)	6.4 (3.3-10.)	8.7 (5.4-16)	6.9 (5.0-8.8)	3.4 (2.0-4.7)	7.1 (6.8-7.3)	6.7 (5.7-8.3)	13 (9.6-20)	5.8 (4.0-8.5)	6.7	6.4	7.1
CD5L	O43866	EATLQDCPSGPWGK	4.3 (3.4-4.8)	5.4 (2.7-7.2)	4.7 (2.1-11)	8.7 (5.7-11)	13 (13-13)	7.9 (3.5-12)	8.6 (5.6-11)	12 (2.5-13)	11 (7.6-15)	8.6	12	7.9
CD5L	O43866	IWLDNVR	3.2 (2.4-4.6)	5.6 (2.6-8.2)	5.1 (2.1-12)	5.5 (2.6-8.4)	5.1 (1.0-9.2)	3.2 (2.0-4.4)	5.9 (5.6-18)	17 (10-26)	7.9 (2.7-14)	5.5	5.6	5.1
APOA4	P06727	LGEVNTYAGDLQK	2.5 (1.9-4.6)	5.0 (3.4-7.3)	6.4 (2.6-10)	4.5 (0.8-8.3)	3.9 (2.4-5.4)	3.2 (3.1-3.3)	5.3 (5.0-18)	13 (11-13)	14. (6.9-20)	4.5	5	6.4
APOA4	P06727	ISASAEELR	3.3 (2.7-6.2)	6.7 (1.8-8.8)	6.1 (2.2-13)	7.1 (5.4-8.9)	7.2 (5.3-9.0)	0.7 (0.4-1.1)	7.2 (4.2-7.8)	8.2 (3.6-13)	5.9 (1.5-20)	7.1	7.2	5.9
APOA4	P06727	LAPLAEDVR	5.0 (2.4-5.9)	8.1 (4.9-10.)	6.6 (2.6-13)	3.6 (1.5-5.8)	6.8 (6.6-7.0)	3.6 (1.2-6.0)	10. (3.7-14)	11 (8.6-14)	7.3 (4.6-14)	5	8.1	6.6
IGJ	P01591	SSEDPNEDIVER	3.8 (1.8-5.1)	6.1 (3.3-9.0)	6.5 (1.9-12)	10 (10-10)	8.8 (8.4-9.3)	2.2 (2.2-2.3)	6.5 (5.9-10)	7.9 (7.8-11)	5.2 (2.0-17)	6.5	7.9	5.2
IGJ	P01591	FVYHLSDLCK	1.8 (1.3-4.5)	4.0 (2.5-5.8)	4.5 (1.3-10)	10 (6.1-15)	12 (7.9-17)	4.5 (3.2-5.7)	10. (5.7-13)	7.2 (7.0-9.6)	14. (4.9-16)	10	7.2	4.5
APOC1	P02654	EFGNTLEDK	3.0 (0.7-6.3)	6.2 (1.8-9.9)	6.9 (1.1-13)	6.8 (2.2-11)	5.2 (3.0-7.4)	3.4 (2.2-4.5)	7.3 (5.9-11)	9.8 (7.0-15)	5.9 (1.0-13)	6.8	6.2	5.9
APOC1	P02654	EWFSETFQK	4.1 (2.8-5.2)	7.2 (3.5-9.7)	5.1 (2.2-9.4)	10 (7.4-12)	9.2 (5.5-12)	1.9 (1.5-2.3)	3.6 (1.8-11)	4.1 (3.5-11)	13 (2.8-13)	4.1	7.2	5.1
IGFBP2	P18065	LEGEACGVYTPR	8.8 (4.9-12.)	5.4 (0.7-10.)	7.9 (5.1-11)	5.7 (1.5-9.9)	4.6 (2.7-6.4)	9.7 (3.4-15)	23 (17-23)	10 (9.0-18)	21 (5.7-26)	8.8	5.4	9.7
IGFBP2	P18065	LIQGAPTIR	4.7 (1.4-5.5)	6.8 (3.2-8.4)	7.9 (6.1-11)	10 (9.2-11.)	6.8 (5.1-8.5)	12. (11-13)	10 (10-19)	10 (10.-13)	12 (10-30)	10	6.8	12
LPA	P08519	GTYSTTVTGR	2.8 (2.4-7.5)	5.8 (1.2-10)	6.7 (1.5-14)	6.3 (2.2-10.)	4.6 (1.1-8.0)	2.3 (2.1-2.6)	7.7 (6.8-11)	11 (9.1-15)	9.1 (2.5-17)	6.3	5.8	6.7

LPA	P08519	NPDAVAAPYCYTR	4.7 (1.9-5.8)	6.6 (4.1-10)	5.7 (2.6-11)	2.5 (1.5-3.6)	2.3 (1.2-3.4)	3.0 (1.5-4.4)	8.5 (3.6-15)	13 (9.7-14)	10 (3.6-18)	4.7	6.6	5.7
TF	P02787	EDPQTFYYAVAVVK	7.1 (5.9-9.3)	7.3 (5.1-12)	12 (9.8-15)	2.8 (2.7-3.0)	2.0 (0.6-3.3)	1.7 (1.0-2.4)	5.2 (2.3-8.9)	10 (9.8-14)	7.5 (1.1-13)	5.2	7.3	7.5
TF	P02787	DGAGDVAFVK	2.1 (1.6-4.8)	5.5 (2.1-9.5)	7.0 (1.8-13)	3.6 (3.4-3.7)	2.5 (1.6-3.4)	1.3 (0.3-2.3)	7.2 (6.5-7.7)	9.9 (9.6-13)	5.3 (3.1-15)	3.6	5.5	5.3
F12	P00748	CLEVEGHR	8.2 (4.2-16)	13 (12-14)	14 (10-19)	9.7 (4.1-15.)	23 (22-23)	21 (16-26)	142 (93-165)	19 (16-44)	149 (76-151)	9.7	19	21
F12	P00748	NGPLSCGQR	4.2 (2.7-14)	9.8 (2.1-11)	7.8 (2.0-19)	3.1 (2.6-3.7)	0.2 (0.2-0.2)	3.1 (2.5-3.7)	7.5 (4.1-12)	13 (8.1-15)	7.0 (4.4-20)	4.2	9.8	7
GC	P02774	HLSLLTTLSNR	5.2 (3.5-7.8)	6.5 (4.0-13)	4.5 (2.1-15)	10 (8.1-12)	13 (10-15)	5.0 (2.4-7.7)	15 (11-19)	20 (4.4-28)	13 (9.5-20)	10	13	5
GC	P02774	VLEPTLK	6.2 (1.2-9.6)	8.7 (3.2-13)	6.5 (2.0-12)	8.9 (8.1-9.6)	8.6 (7.0-10)	0.9 (0.5-1.4)	5.4 (2.9-7.2)	8.6 (5.5-11)	5.0 (4.7-15)	6.2	8.6	5

SUPPLEMENTARY FIGURES

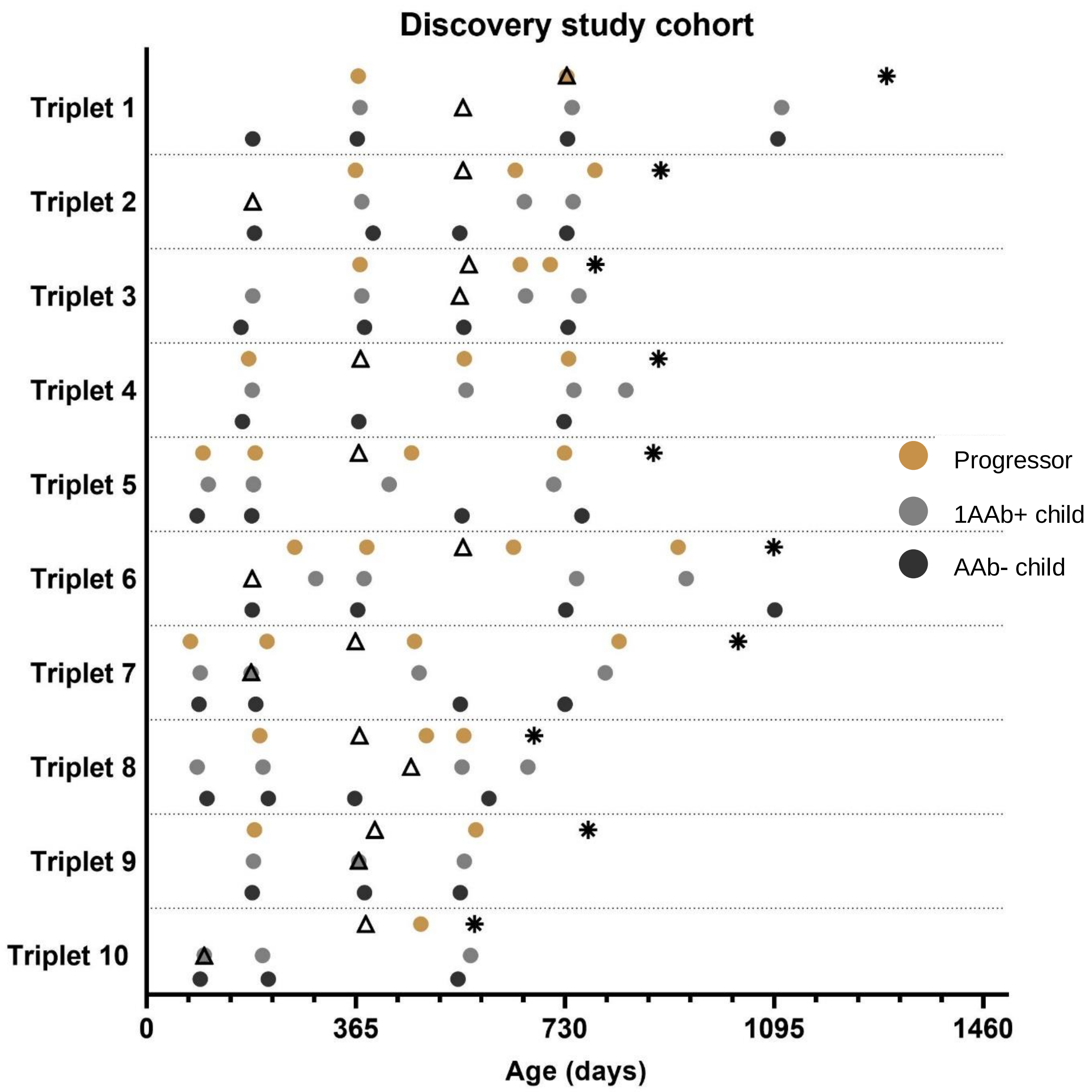

Supplementary Fig. S1. Longitudinal plasma sample collection times of the discovery proteomics study participants. Triangle indicates the date of the appearance of the first autoantibody. Star indicates the date of type 1 diabetes diagnosis. Progressor = child who has developed multiple autoantibodies and progressed to type 1 diabetes before 4 years of age. 1AAb+ child = single autoantibody positive child without progression to type 1 diabetes. AAb-child = autoantibody negative child

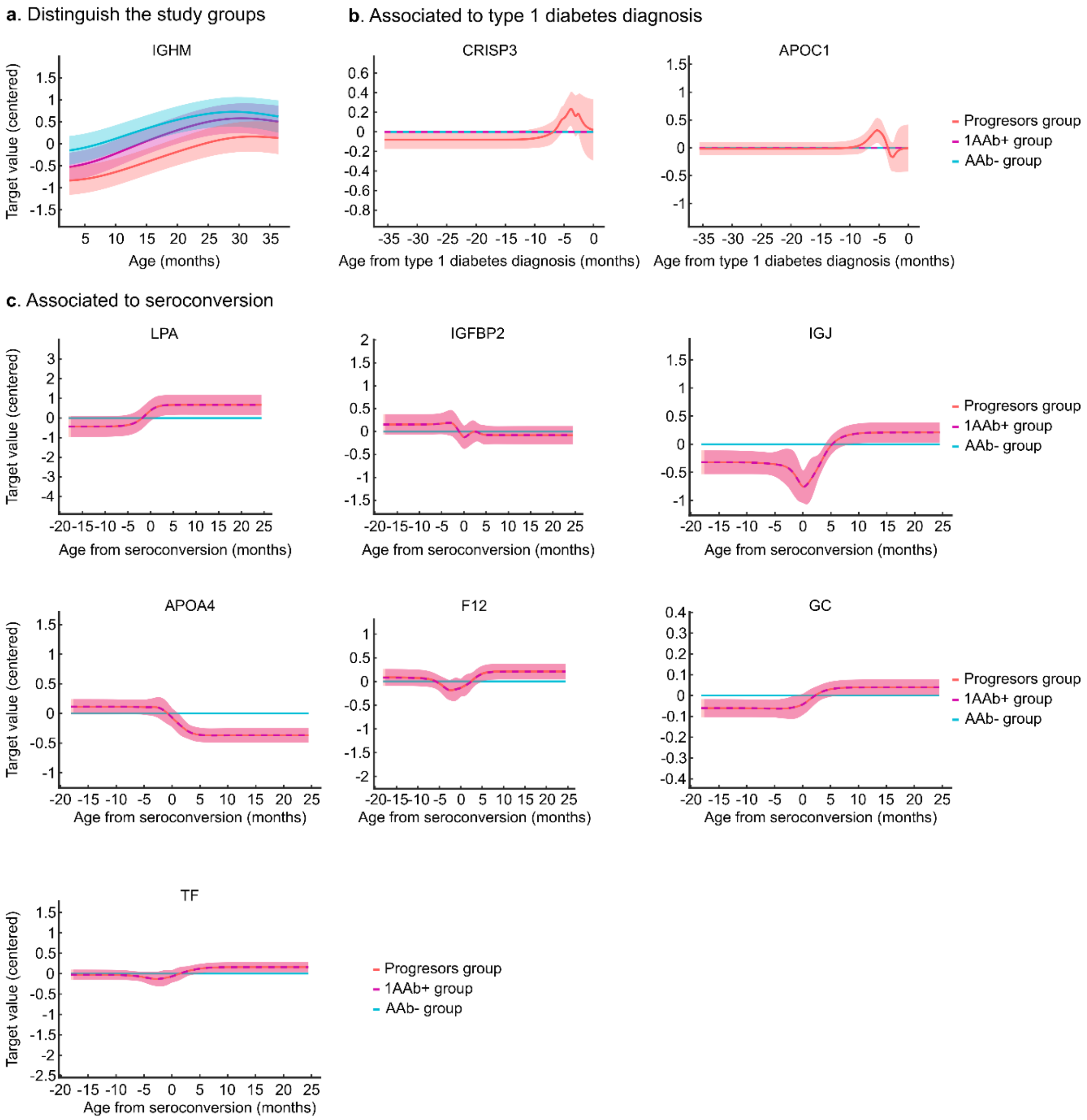

Supplementary Fig. S2. Discovery proteomics results of the disease-associated proteins selected for the verification study using targeted proteomics. (a) We first selected proteins that showed differences between the three study groups (with "group" and "group*age" terms in LonGP). (b) Then we selected the proteins that exhibited changes associated with the disease diagnosis ("t1d" term in LonGP). (c) Lastly, the selection was made among proteins that showed changes associated with the seroconversion ("sero" term in LonGP). The shaded areas are 95% confidence intervals. Progressors group = children who have developed multiple autoantibodies and progressed to type 1 diabetes before 4 years of age, 1AAb+ group = single autoantibody positive children without progression to type 1 diabetes. AAb- group = autoantibody negative children.

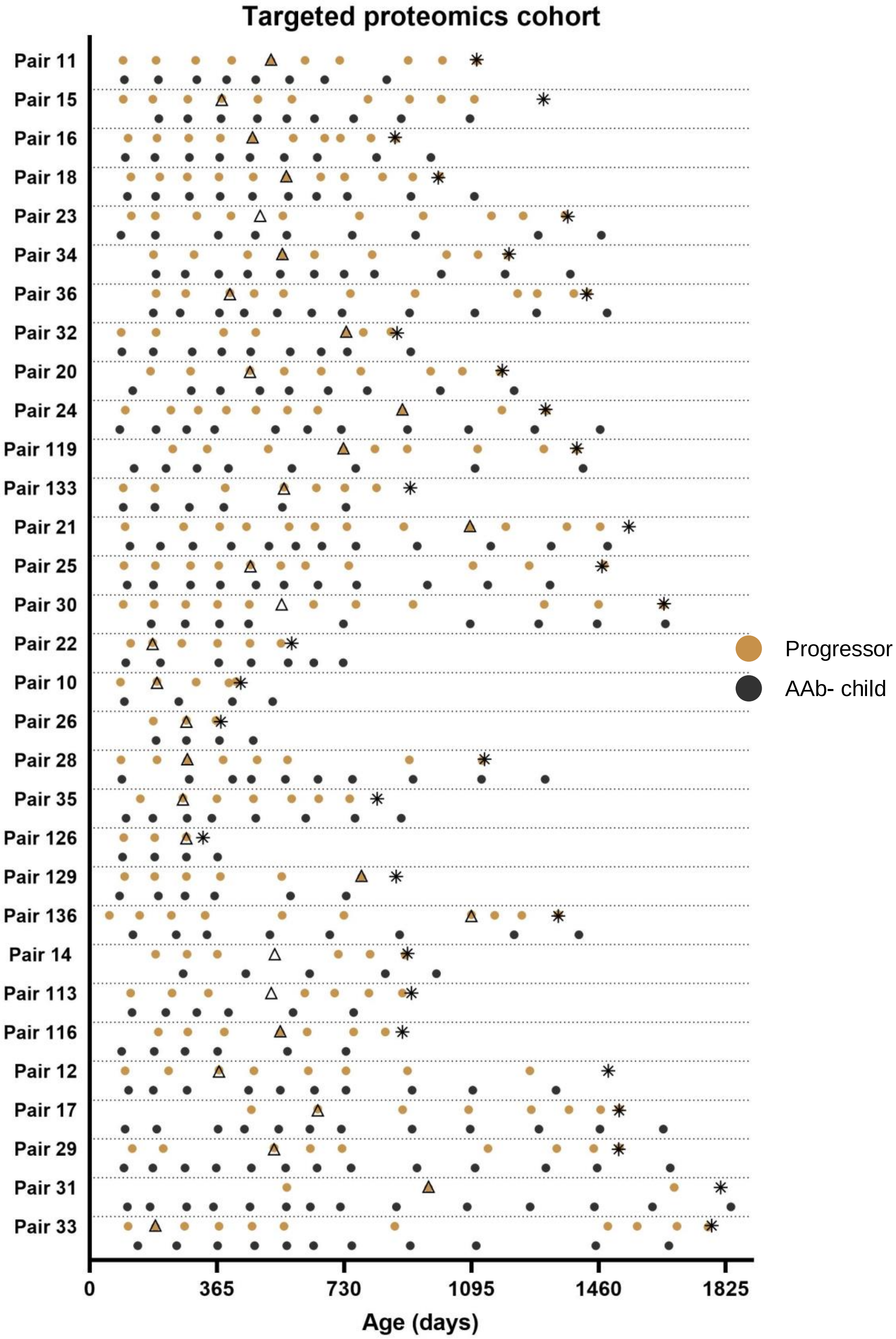

Supplementary Fig. S3. Longitudinal serum sample collection times of the targeted proteomics study participants. Triangle indicates the date of the appearance of the first autoantibody. Star indicates the date of type 1 diabetes diagnosis. Progressor = child who has progressed to type 1 diabetes before 5 years of age. AAb- child = autoantibody negative child.

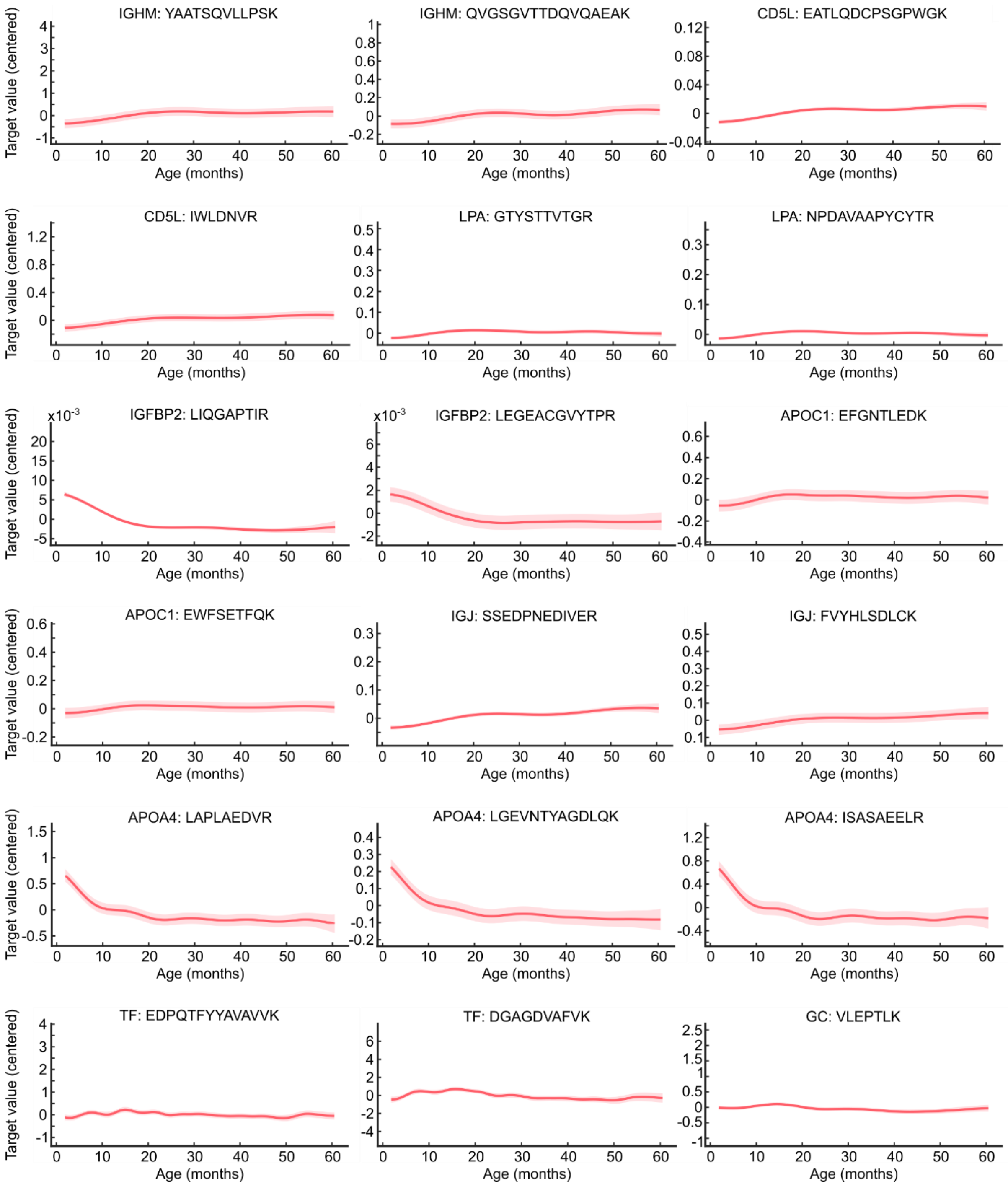

Supplementary Fig. S4. Peptides with age associated trends in the targeted proteomics data (“age” term in LonGP). The shaded areas are 95% confidence intervals.

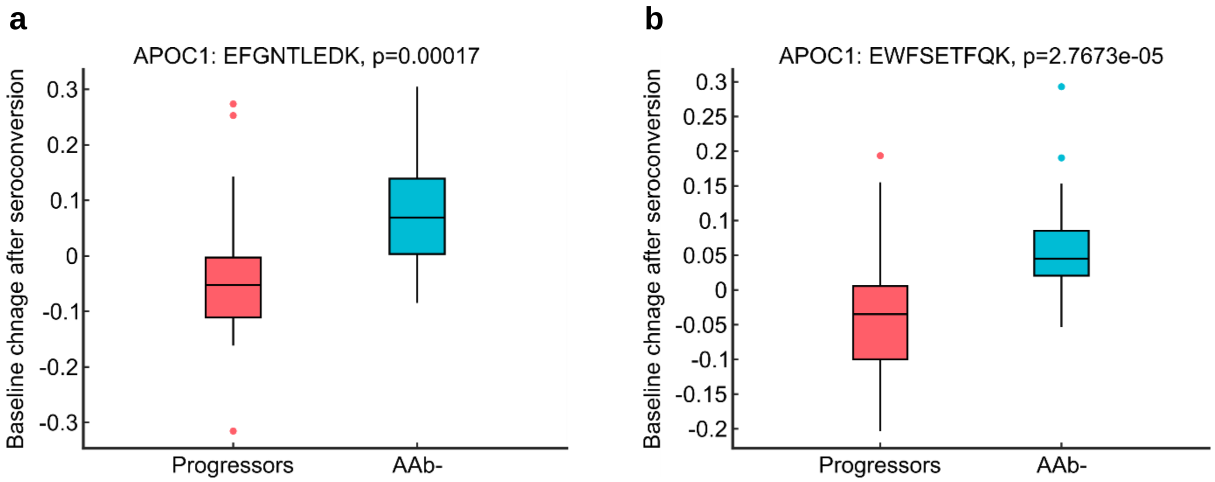

Supplementary Fig. S5. Baseline change after seroconversion (baseline_after – baseline_before) of two APOC1 peptides: (a) EFGNTLEDK and (b) EWFSETFQK in the validation data. For each individual, we calculated the baseline peptide levels before and after the seroconversion by computing the average of data points before and after seroconversion. The seroconversion age of an AAb- is taken from its matched progressor. Progressors (red) = children who have progressed to type 1 diabetes before 5 years of age. AAb- (blue) = autoantibody negative children. Two sample t-test is perform to check the difference between progressors and AAb- children.

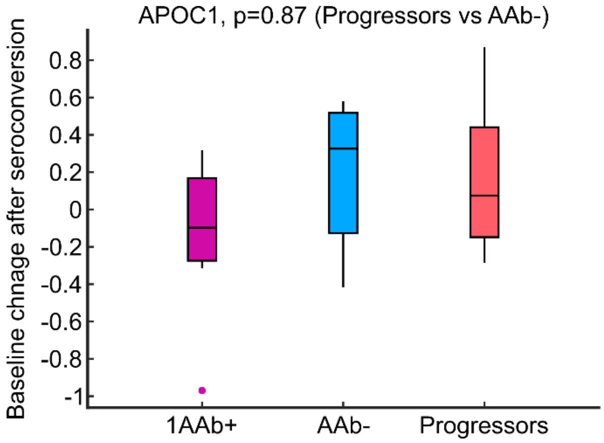

Supplementary Fig. S6: Baseline change after seroconversion (baseline_after – baseline_before) for APOC1 in the discovery data. For each individual, we calculated the baseline protein levels before and after the seroconversion by computing the average of data points before and after seroconversion. The seroconversion age of an AAb- is taken from its matched progressor. Progressors (red) = children who have developed multiple autoantibodies and progressed to type 1 diabetes before 4 years of age. 1AAb+ (purple) = single autoantibody positive children without progression to type 1 diabetes. AAb- (blue) = autoantibody negative children. Two sample t-test is perform to check the difference between progressors and AAb- children.

a

b

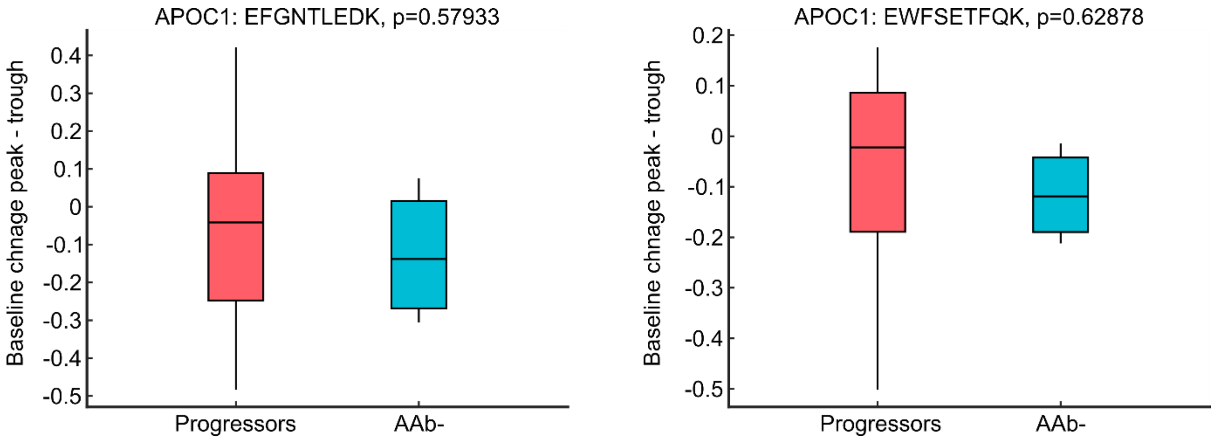

Supplementary Fig. S7. t1d related baseline change (peak - trough) of two APOC1 peptides: (a) EFGNTLEDK and (b) EWFSETFQK in the validation data. Peak region is defined as [-7, -5], i.e. 5 to 7 months before type 1 diabetes diagnosis. Trough region is defined as [-4, -2], i.e. 2 to 4 months before type 1 diabetes diagnosis. For each individual, we calculated the baseline peptide levels for the peak or trough regions by computing the average of data points that fall into the corresponding regions, where only individuals with data points in both regions are included. Progressors (red) = children who have progressed to type 1 diabetes before 5 years of age. AAb- (blue) = autoantibody negative children.

a b

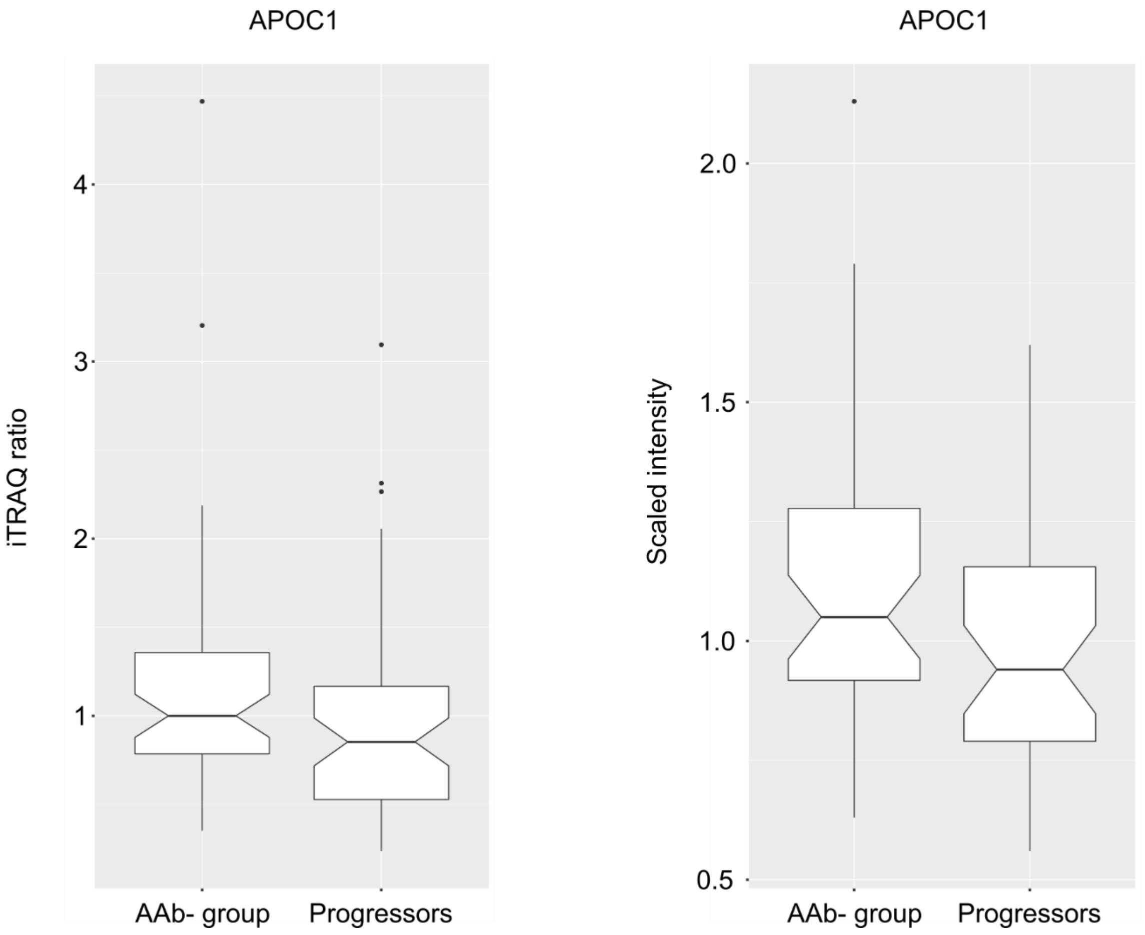

Supplementary Fig. S8. APOC1 intensities in the study of Moulder et al. 2015. (a) iTRAQ ratios of APOC1 scaled to a common reference from eight matched pairs. In addition, there were five pairs with missing APOC1 values who were excluded from the analysis. (b) Scaled label-free intensities from the six matched case-control pairs. Progressors = progressed to type 1 diabetes between 2.2 and 12.1 years of age. AAb- group = autoantibody negative children.
